# Novel *N*-Arylsulfonylindoles Targeted as Ligands of the 5-HT_6_ Receptor. Insights on the Influence of C-5 Substitution on Ligand Affinity

**DOI:** 10.3390/ph14060528

**Published:** 2021-06-01

**Authors:** Loreto Arrieta-Rodríguez, Daniela Espinoza-Rosales, Gonzalo Vera, Young Hwa Cho, David Cabezas, David Vásquez-Velásquez, Jaime Mella-Raipán, Carlos F. Lagos, Gonzalo Recabarren-Gajardo

**Affiliations:** 1 Bioactive Heterocycles Synthesis Laboratory (BHSL), Departamento de Farmacia, Facultad de Química y de Farmacia, Pontificia Universidad Católica de Chile, Avenida Vicuña Mackenna 4860, Macul, Santiago 7820436, Chile; liarriet@uc.cl (L.A.-R.); dmespino@uc.cl (D.E.-R.); glvera@uc.cl (G.V.); aycho@uc.cl (Y.H.C.); 2 Instituto de Química y Bioquímica, Facultad de Ciencias, Universidad de Valparaíso, Avenida Gran Bretaña 1111, Valparaíso 2360102, Chile; david.cabezas@postgrado.uv.cl (D.C.); jaime.mella@uv.cl (J.M.-R.); 3 Centro de Investigación Farmacopea Chilena (CIFAR), Universidad de Valparaíso, Santa Marta 183, Valparaíso 2360134, Chile; 4 Drug Development Laboratory, Faculty of Chemical and Pharmaceutical Sciences, Universidad de Chile, Sergio Livingstone 1007, Santiago 8380492, Chile; dvasquez@ciq.uchile.cl; 5 Chemical Biology & Drug Discovery Lab, Escuela de Química y Farmacia, Facultad de Medicina y Ciencia, Universidad San Sebastián, Lota 2465, Providencia, Santiago 7510157, Chile; carlos.lagos@uss.cl; 6 Centro Interdisciplinario de Neurociencias, Pontificia Universidad Católica de Chile, Marcoleta 391, Santiago 8330024, Chile

**Keywords:** arylsulfonylindoles, 5-HT_6_ receptor, serotonergic ligands, docking, CoMFA, CoMSIA

## Abstract

A new series of twenty-two C-5 substituted *N*-arylsulfonylindoles was prepared with the aim of exploring the influence of C-5 substitution on 5-HT_6_ receptor affinity. Eleven compounds showed moderate to high affinity at the receptor (*K*_i_ = 58–403 nM), with compound **4d** being identified as the most potent ligand. However, regarding C-5 substitution, both methoxy and fluorine were detrimental for receptor affinity compared to our previously published unsubstituted compounds. In order to shed light on these observations, we performed docking and molecular dynamics simulations with the most potent compounds of each series (**4d** and **4l**) and **PUC-10**, a highly active ligand previously reported by our group. The comparison brings about deeper insight about the influence of the C-5 substitution on the binding mode of the ligands, suggesting that these replacements are detrimental to the affinity due to precluding a ligand from reaching deeper inside the binding site. Additionally, CoMFA/CoMSIA studies were performed to systematize the information of the main structural and physicochemical characteristics of the ligands, which are responsible for their biological activity. The CoMFA and CoMSIA models presented high values of q^2^ (0.653; 0.692) and r^2^ (0.879; 0.970), respectively. Although the biological activity of the ligands can be explained in terms of the steric and electronic properties, it depends mainly on the electronic nature.

## 1. Introduction

The serotonergic system modulates a diverse variety of physiological functions, such as thermoregulation, sexual and aggressive behavior, learning and memory, endocrine and gastrointestinal functions, and food intake, among others [1]. In order to perform these different tasks, the neurotransmitter 5-hydroxytryptamine (5-HT) interacts with various receptor subtypes among which the 5-HT_6_ receptor (5-HT_6_R) is one of the most recently discovered, about a quarter of a century ago [2]. The 5-HT_6_R is involved in several physiological functions, such as learning, memory, and neurodevelopment, and diverse pathological states, such as eating disorders, anxiety, depression, addictive behavior, schizophrenia, epilepsy and Alzheimer’s disease [3,4,5,6,7,8]. 5-HT_6_ receptor ligands (specially antagonists) have been shown to have procognitive behavioral effects, making them potential cognitive enhancers in conditions associated with cognitive impairment, such as Alzheimer’s disease, schizophrenia, and autism spectrum disorder, among others [9,10,11,12,13]. 

**Figure 1:**
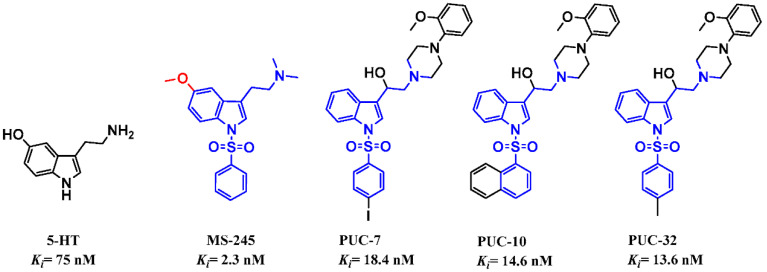
Structures and inhibition constants (K*_i_*) for 5-HT [18], MS-245 [19,20], and examples of our previously reported *N*-arylsulfonylindole antagonists over the 5-HT_6_ receptor [17].

To date, an overwhelming majority of 5-HT_6_ ligands, both agonists and antagonists, share the following structural features: a basic ionizable amine group (PI), a sulfonamide moiety as a hydrogen bond acceptor group (HBA) connected to a hydrophobic site (HYD) and a π-electron donor aromatic or heterocyclic ring (AR) [9,14]. However, a few reports claim that the PI pharmacophoric feature is not essential for receptor affinity [15,16]. In this context, and given our interest in exploring the limits of the -HT_6_ ligand pharmacophore, we have previously reported the design, synthesis and pharmacological evaluation of a number of extended and weakly basic *N*-arylsulfonylindole derivatives with moderate to high affinity and antagonistic profile toward the 5-HT_6_ receptor [17], where our structural scaffold could be regarded as a masked and extended *N*-arylsulfonyltryptamine, similar to MS-245 (Figure 1). In this work, the activity of the ligands was attributed to an additional hydrogen bond between the alcohol group and Asp106 (3.32) and to the interaction of the aromatic ring linked to piperazine with a second hydrophobic pocket, proposed from molecular docking studies.

Considering the excellent affinity presented by several 5-methoxy arylsulfonyltryptamines derivatives, similar to MS-245 [19,20], we decided to explore the influence of this substitution pattern on our structural scaffold. To have a comparative insight regarding the substitution on C-5 of the indole ring and ligand affinity, we also proposed the synthesis of analogs bearing fluorine as an electron-withdrawing atom. Therefore, in this work, we report the synthesis, affinity evaluation over the 5-HT_6_ receptor, molecular modeling of receptor–ligand interactions through molecular dynamic simulations, and 3DQSAR studies of twenty-two new *N*-arylsulfonylindole compounds with C-5 substitution. 

## 2. Results and Discussion

### 2.1. Synthesis 

**Scheme 1:**
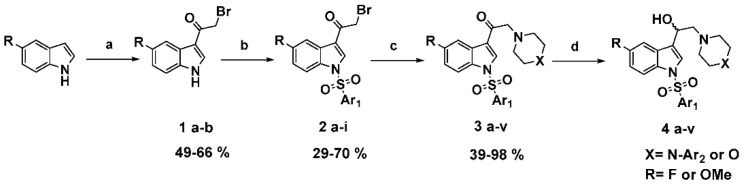
Synthesis of derivatives **4a**–**v**, reagents and conditions: **a**: (i) Anhydrous ZnCl_2_, dry CH_2_Cl_2_, CH_3_MgBr, rt, 2 h; (ii) BrCH_2_COCl, 12 h; (iii) NH_4_Cl sat.; **b**: ArSO_2_Cl, Et_3_N, DMAP, CH_2_Cl_2_, rt.; **c**: Arylpiperazines or morpholine, K_2_CO_3_, acetone, rt.; **d**: NaBH_4_/MeOH.

The synthesis of the new *N*-arylsulfonylindoles targeting the 5-HT_6_ receptor (**4a**–**v**) was performed as is briefly described in Scheme 1 (for details, see the experimental section). First, the synthesis of C-5 substituted 3-bromoacetylindoles **1a**–**b** was performed following a previously reported method for benzenesulfonyltriptamines, which has already been used by our group with slight modifications [17,21,22]. Acylation was performed in a one-pot, two-step sequence, starting from adequately substituted commercial indoles (5-methoxyindole and 5-fluoroindole) and methyl magnesium bromide to provide the corresponding magnesium salts, followed by a transmetallation reaction with anhydrous zinc chloride. Then, the zinc salts were acylated using bromoacetyl chloride under an inert atmosphere to afford bromoacetylindoles **1a**–**b** in good yields. 

The previously obtained 5-fluoro and 5-methoxy bromoacetylindoles **1a**–**b** were further protected with the appropriate aromatic sulfonyl chlorides under basic conditions to afford the corresponding C-5 substituted *N*-arylsulfonyl-3-bromoacetylindoles **2a**–**i** with moderate to good yields [17,23]. In the next step, the prepared haloketones were subjected to bromide displacement in a basic medium at room temperature with various arylpiperazines or morpholines to obtain the respective functionalized ethanones **3a**–**v** in moderate to excellent yields [17,24]. Ketones obtained after the *N*-alkylation reaction described above were subsequently reduced with sodium borohydride in methanol to obtain the corresponding alcohols **4a**–**v** as racemic mixtures. The structures and purity of the new compounds were confirmed through spectral (^1^H-NMR, ^13^C-NMR, IR, HRMS) and chromatographic (TLC) methods. The assignment of the signals from the NMR spectra of the synthesized compounds was carried out by analyzing the multiplicity of the signals and their corresponding coupling constants, and by comparison with the NMR spectra of the unsubstituted C-5 compounds previously reported by us.

### 2.2. Radioligand Binding Assays

Synthesized C-5 substituted *N*-arylsulfonylindole derivatives were tested in standard radioligand competition binding assays, using HEK-293 cell membranes expressing a recombinant human 5-HT_6_ receptor. The compounds were assayed as free bases at eight different concentrations in triplicate in order to obtain the dose response curves, determine the half maximal inhibitory concentration (IC_50_) values, and calculate the inhibitory constant (*K*_i_) values for each one of them (Table 1). Additionally, the structures and *K*_i_ values of some unsubstituted derivatives previously reported by our research group are shown.

All tested compounds exhibited inhibition of [^125^I]-SB-258585 [26] binding to 5-HT_6_ receptors (radioligand displacement). Eleven of the twenty-two compounds showed an important binding affinity for the 5-HT_6_ receptor (equal or better than 400 nM of *K*_i_). However, C-5 substitution, regardless of the substituent, was detrimental for affinity compared to our previous set of ligands within this structural framework [17].

In general, the series with a 5-methoxy substituent exhibited better affinity than the series with 5-fluorine (eight compounds with *K*_i_ values below 400 nM including four below 200 nM), even though the best compound of all series was the 5-fluoro derivative **4d** with a *K*_i_ value of 58 nM. This ligand was the only compound that was better than the endogenous ligand 5-HT and it showed a *K*_i_ in the same order of magnitude as the most potent unsubstituted compounds **PUC-7**, **PUC-10**, and **PUC-32** (See Figure 1 and Table 1). However, this result was not completely surprising given the substitution pattern of **4d**, which is like **PUC-10**. The analogous 5-methoxy derivative **4l** also exhibited a good affinity (*K*_i_ 160 nM), but lower than that of **4d** and **PUC-10**. Both **4d** and **4l** ligands have a 2-methoxyphenyl group on Ar_2_, as do five other compounds (**4a**, **4i**, **4j**, **4r** and **4u**). All these seven ligands are included within the eleven most potent compounds. Thus, the 2-methoxyphenyl substitution in the piperazine ring was revealed as the best-performing group. These results are in agreement with our previous report of unsubstituted compounds [17]. A more specific comparison between both series of C-5 substituted ligands indicated that 5-methoxy derivatives were superior to 5-fluoro derivatives as is shown for the following pairs of ligands (except for the mentioned pair of molecules **4d** v/s **4l**): **4j** v/s **4a** (*K*_i_: 289 nM v/s 390 nM); **4k** v/s **4c** (*K*_i_: 403 nM v/s 12,515 nM); **4n** v/s **4e** (*K*_i_: 153 nM v/s 801 nM); **4m** v/s **4f** (*K*_i_: 247 nM v/s 683 nM); **4u** v/s **4h** (*K*_i_: 270 nM v/s 850 nM); and **4r** v/s **4i** (*K*_i_: 167 nM v/s 381 nM). 

Regarding the Ar_1_ substitution pattern, in this work we only employed electron-rich rings, given that our previous results indicated that poor electron density rings were detrimental for affinity [17]. In this way, we confirmed our previous observations that the 1-naphthyl group is the best ring to be used as a hydrophobic region, given that three of the five best compounds bear this substitution in Ar_1_ (**4d**, **4l** and **4n**). Regarding the substitution in Ar_1_, we also observed that two of the five best compounds bear a 5-bromo-2-thiophenyl group (**4r** and **4s** with *K*_i_ 167 nM and 130 nM respectively). This group follows the structural guidelines formulated in our previous work on the requirement of electron-rich aromatic or heteroaromatic groups in that position [27]. The affinities showed by derivatives bearing a 5-bromo-2-thiophenyl substitution at Ar_1_ are similar to those which possess the *p*-iodophenyl groups, although the first ones proved to be slightly potent compared to *p*-iodophenyl derivatives (**4i** v/s **4a**; **4r** v/s **4j** and **4s** v/s **4k**). This result could suggest the existence of a halogen bond between these ligands and the active site of the receptor. It is noteworthy that a similar halogen bond interaction was previously described by López-Rodríguez in a series of dimethylaminoethyl-1*H*-benzimidazol-yl-aryl-sulfonamides in an analogous position (i.e., the halogen group is pending from an aryl group of the sulfonamide) [9]. It is worth mentioning that in our previous work on the matter, a derivative containing a *p*-iodophenyl group presented high affinity (**PUC-7**), equipotent with **PUC-10**, the most active compound of the series (see Figure 1) [17].

An interesting observation could be made by comparing these C-5 substituted derivatives with the corresponding unsubstituted derivatives that were previously reported (see Table 1). In addition to the lower activity found for compounds **4d** and **4l** when compared with **PUC-10**, both **4a** and **4j** exhibited decreased affinity at the 5-HT_6_ receptor of more than one order of magnitude compared to **PUC-7**. In addition, the fluorine derivative **4b** exhibited ten times less affinity than its unsubstituted derivative **PUC-9**. Similarly, compound **4g** showed half the affinity of its unsubstituted derivative **PUC-12**. On the other hand, the methoxylated derivative **4t** had approximately eight times less affinity than **PUC-22**, the unsubstituted derivative. The ligand **4p** (*K*_i_ = 2195 nM) was approximately four times less active than the unsubstituted **PUC-24** (*K*_i_ = 479 nM). When comparing derivatives **4o** (*K*_i_ = 972 nM) and **PUC-32** (*K*_i_ = 13.6 nM), the same trend could be observed. Perhaps the only anomalous result in this trend was presented by the trio **4e**, **4n** and **PUC-11**. In this case, the fluorine-containing derivative was less potent than the unsubstituted derivative PUC-11 (*K*_i_ = 801 nM vs. 148 nM), but the methoxylated ligand **4n** (*K*_i_ = 153 nM) was equipotent with **PUC-11**. Overall, the results seemed to indicate that whatever C-5 substitution is used, this is detrimental for affinity, and in the best case, it is not better than its absence. With the aim of attaining better knowledge about this behavior, we performed molecular docking studies and molecular dynamic simulations over the most potent ligand of this new series **4d**, its methoxylated analog **4l** and the best overall compound **PUC-10**. 

### 2.3. Docking Studies

Previously, we reported **PUC-10** as a high affinity antagonist of the 5-HT_6_ receptor (*K*_i_ = 14.6 nM) [17]. Compounds **4l** and **4d** correspond to the C-5 substituted methoxylated and fluorinated analogues of **PUC-10**. Although the affinity of both compounds was not completely abolished, **4d** diminished its affinity by 4-fold (*K*_i_ = 58 nM) compared to **PUC-10**, whereas **4l** diminished its affinity by a factor of 11 (*K*_i_ = 160 nM). 

**Figure 2:**
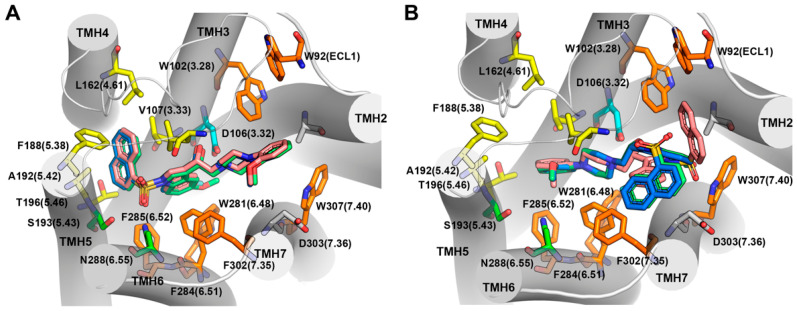
FRED-obtained binding modes for compounds **PUC-10** (sky-blue), **4d** (deep salmon) and **4l** (lemon) in (**A**) *S* conformation and (**B**) *R* conformation. Residues in orange depict the hydrophobic cluster in TMH-6 and TMH7 regions; yellow depicts residues in TMH-3 and TMH-5 surrounding the binding pocket, cyan depicts Asp106 (3.32) implicated in Scheme 288. (6.55) and Ser193 (5.43) implicated in hydrogen bond interactions (residues are also indicated under Ballesteros–Weinstein nomenclature [28]).

To gain further molecular insights, these compounds were docked into the 5-HT_6_ receptor model, and the *R*/*S* stereoisomers of each compound were analyzed separately. The results presented in Figure 2 show that molecules with the hydroxy group in the *S* configuration allowed the placement of the naphthalene ring into a cavity created between TMH-3 and 5, and the phenylpiperazinyl moiety oriented toward TMH-2 and 7 (Figure 2A). On the other hand, molecules in *R* conformation flipped their binding mode by 180 degrees, with the naphthalene ring extending toward TMH-2 and 7 regions and the phenylpiperazinyl moiety fitting within the cavity between TMH-3 and 5 (Figure 2B).

Although the crystal structure of the 5-HT_6_ receptor was still not available, efforts to describe the orthosteric binding pocket indicated that the key residues included: Asp106 (3.32), which assists in ligand recognition by forming a salt-bridge with charged ligands; Asn288 (6.55), which forms hydrogen bond interactions with HBAs in the ligand; Val107 (3.33), Cys110 (3.36), Ala192 (5.42), Ser193 (5.43) that surround the orthosteric binding pocket; and the hydrophobic cluster on TMH-6 comprised of Trp281 (6.48), Phe284 (6.51) and Phe285 (6.52) [29]. Arylindoles are reported to bind into the described pocket by inserting the indole nucleus into the hydrophobic cluster on TMH-6 with the aryl group extending between TMH-3 and TMH-5 and interacting with the surrounding residues Val107 (3.33) and Ala192 (5.42) [14]. Based on this literature evidence, we reasoned that *S* stereoisomers better fit the reported binding model of arylindoles in the 5-HT_6_ receptor. Moreover, considering the hydrophobic nature of the naphthalene ring, it is more reasonable for this moiety to plunge deeper inside the receptor rather than face toward the extracellular region (as in the case of *R* stereoisomers), where the binding pocket becomes more hydrophilic. 

**Figure 3:**
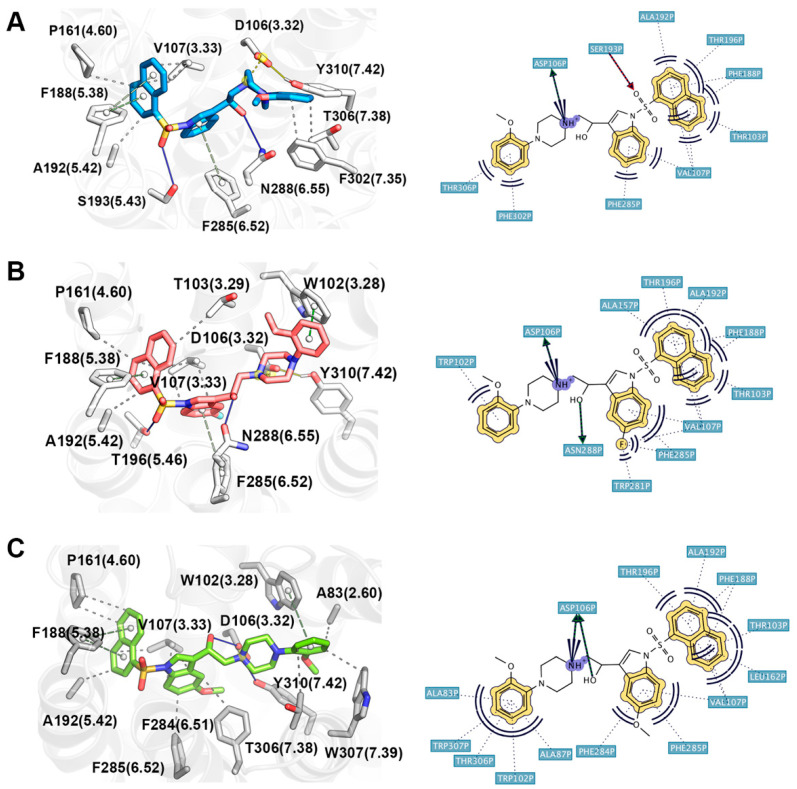
Binding modes obtained after MD simulation of compounds (**A**) **PUC-10** (sky-blue), (**B**) **4d** (deep salmon) and (**C**) **4l** (lemon) depicted from the extracellular view. The 2D interaction plots were obtained with LigandScout v4.2 (Inte:Ligand GmbH).

Molecules in the *S* configuration were further accommodated within the binding pocket by running short MD simulations. The trajectory was then clustered using cpptraj [30], and the final interactions of the ligand–receptor complex were analyzed through the PLIP web server (Figure 3) [31]. The key salt bridge between Asp106 (3.32) and the piperazinium cation was maintained for all three compounds. In addition, a hydrogen bond was established between Asp106 (3.32) and Tyr310 (7.42), which aided in the stabilization of the ligand. This interaction network between Asp106 (3.32), Tyr310 (7.42) and the ligand is present in many crystal structures of aminergic receptors and is thought to facilitate the interaction of the amine group of the ligand with the receptor [32]. Another common binding feature for all three compounds was the insertion of the naphthalene ring into the hydrophobic cavity between TMH-3 and TMH-5, thereby interacting with hydrophobic and aromatic side chains that included P161 (4.60), F188 (5.39), V107 (3.33) and A192 (5.42). 

A difference in the established interactions involved the hydrogen bonding with residue Asn288 (6.55), which has been implicated as an important residue for 5-HT_6_ antagonist recognition [33]. In the case of **PUC-10** and **4d** (Figure 3A,B), the -OH group, which is in an alpha position to the indole ring, flipped its orientation to establish a hydrogen bond with Asn288 (6.55). However, in **4l** (Figure 3C), this -OH group maintained an interaction with Asp106 (3.32) and did not interact directly with Asn288 (6.55). Regarding the indole ring, it is worth noting the depth that it reaches in each compound within the hydrophobic cluster of TMH-6. **PUC-10** plunged the deepest into the pocket, followed by **4d** and then by **4l** (Figure 3); the magnitude was in accordance with *Ki* values. 

The GPCRs activation mechanism involves the upward movement of TMH-6 toward the extracellular compartment; therefore, the interaction of antagonists with residues in this helix would be pivotal to stabilize the receptor in its inactive form [34]. That said, **PUC-10** and **4d** lowered the indole ring sufficiently to establish an edge-to-face π interaction with Phe285 (6.52) from TMH-6. This residue has been identified as important for antagonist binding through site-directed mutagenesis [33]. However, in the case of **4l**, the methoxy substituent imposed steric hindrance, preventing sufficient lowering of the indole ring to establish a π-stacking with Phe285 (6.52). Instead, **4l** interacted with the aromatic residue through the energetically weaker hydrophobic interaction. As for the case of **4d**, electron withdrawing groups, such as -F, decreased the binding energy of the T-shape aromatic interactions when the group was substituted on the facial aromatic system [35]. Therefore, is it suggested that the interaction of **4d** with Phe285 (6.52) should be weaker than that established by **PUC-10** (Table 2, Table 3 and Table 4).

### 2.4. CoMFA and CoMSIA Studies

Quantitative structure–activity relationship (QSAR) studies allow systematizing the information of the main structural and physicochemical characteristics for a series of compounds, which are responsible for their biological activity. In this way, these studies minimize the error of a qualitative SAR analysis and allow the design of new compounds based on the information obtained.

In order to find the best models, a sequential search for the best field combinations for comparative molecular field analysis (CoMFA) and comparative molecular similarity indices analysis (CoMSIA) was carried out (34 combinations of steric + electrostatic + hydrophobic + hydrogen-bond donor + hydrogen-bond acceptor; Table S1 in Supplementary Materials). The best models obtained are presented in Table 5.

As is shown in Table 5, both final models have a value of q^2^ > 0.5 and high r^2^_test_ values (0.786 and 0.726 for CoMFA and CoMSIA respectively). An equilibrium can be seen in terms of the steric and electrostatic contributions to biological affinity. However, in both cases, the electrostatic contribution is higher. Thus, these properties can play a more important role in the affinity. To validate both models, a test set study was carried out for both models. In Table S2 of Supplementary Materials, we report the values of the external validation. In both cases, both models passed validation. Likewise, the experimental affinity values versus predicted values, in logarithmic scale (p*K*_i_ = −log*K*_i_) for each compound according to the CoMFA and CoMSIA models, are also shown in Table S3 (Supplementary Material).

**Figure 4:**
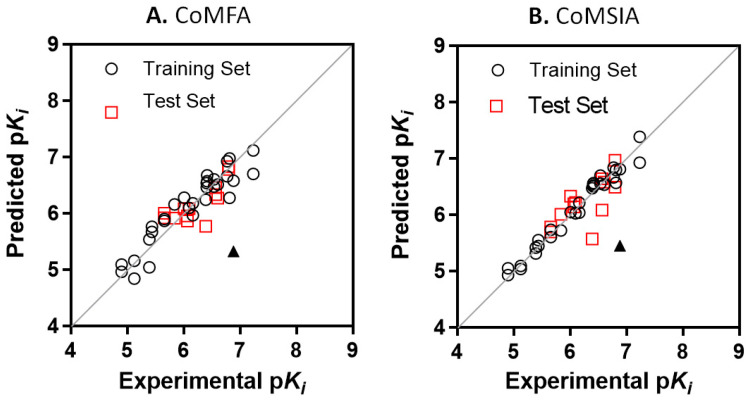
**Figure 4**. Plots of experimental versus predicted p*K*_i_ values for the training and test set molecules of (**A**) CoMFA and (**B**) CoMSIA. The only outlier compound (residual > 1.0) in both cases was compound **4s**, represented as a triangle in the figure.

The CoMFA model presented 24 molecules with a negative residual (p*K*_iexp_ − p*K*_ipred_) and 22 with a positive residual, while CoMSIA presented 28 predictions with negative residual and 18 with positive residual. Therefore, the CoMFA model presents the best equilibrium in terms of predictive capability, while CoMSIA tends to overestimate prediction values. Figure 4A,B shows the distribution of experimental versus predicted values for CoMFA and CoMSIA. As can be seen in both cases, one compound deviated in more than one logarithmic unit in the predicted value of *K*_i_ (compound **4s**, represented as a triangle). All other training and test set compounds had an adequate distribution along the *y = x* straight line. The best value fit was obtained for the CoMSIA model.

**Figure 5:**
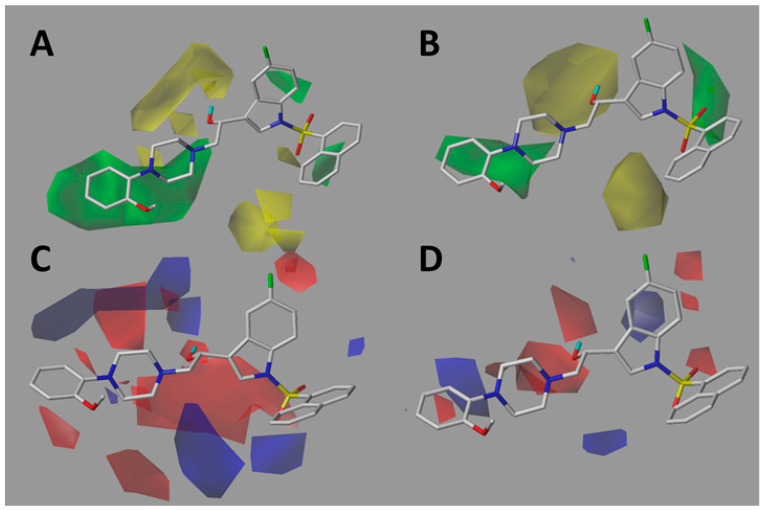
Graphics results of the CoMFA and CoMSIA studies. (**A**) CoMFA steric contour map for Table 4. **4d** (p*K*_i_ = 7.234) and (**B**) CoMSIA steric contour map. Green polyhedrons indicate that it is favorable for biological activity to use bulky substituents, while yellow polyhedrons indicate that it is not favorable. (**C**) CoMFA electrostatic contour map for the most active molecule **4d** and (**D**) CoMSIA electrostatic contour map. Blue polyhedrons indicate that the presence of electron-deficient zones is favorable for activity, while red polyhedrons indicate that electron-rich zones are favorable.

Unlike a 2D-QSAR, the results of a 3D-QSAR can be represented as contour maps around the molecules of the study. The analysis of the different colored polyhedrons around a molecule (e.g., the most active compound in the series) allows understanding the main characteristics that are favorable or unfavorable for affinity or biological activity. Figure 5 shows the results of the steric (Figure 5A,B) and electrostatic (Figure 5C,D) maps for CoMFA and CoMSIA over the most active compound of our synthetic series (derivative **4d**).

**Figure 6:**
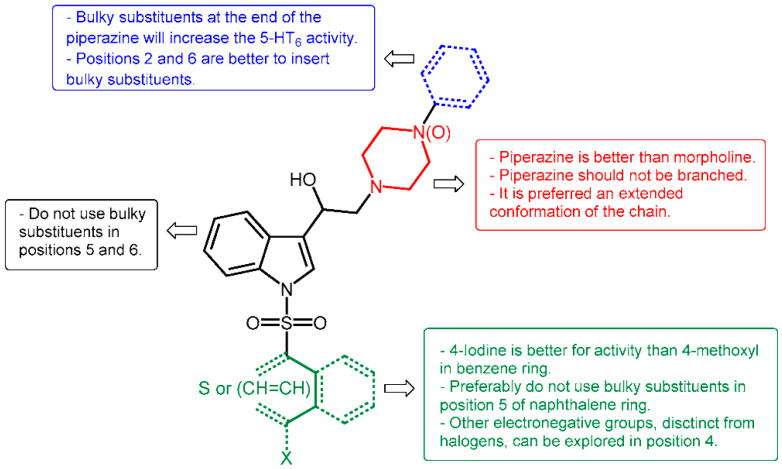
The summary of the SAR founded in the 3D-QSAR study.

The CoMFA steric contour map (Figure 5A) shows a green polyhedron in the phenyl-piperazine zone, that is, it is favorable for biological activity to use bulky substituents in that position. The less active compounds present a morpholino group instead of the aryl-piperazine fragment, which may partly explain the lower activity of these derivatives. CoMSIA steric contour map (Figure 5B) is consistent with this information but shows an additional green polyhedron close to the sulfonyl group region; therefore, the direct connection of bulky rings or alkyl groups to the nitrogen atom of the indole ring would be an interesting option to explore. Close to the hydroxyl group, both the CoMFA and CoMSIA steric contour maps (Figure 5A,B) show a yellow polyhedron, suggesting that the use of low-volume functional groups in that area is most appropriate. The elimination of the hydroxyl group could be evaluated, although there are additional electronic factors linked to this functional group. Finally, near the naphthyl ring, there is a set of yellow polyhedrons in CoMFA (Figure 5A) and a large yellow polyhedron in the steric map of CoMSIA (Figure 5B). Therefore, the insertion of bulky groups at positions 6 and 7 of the naphthyl group should be avoided. On the other hand, the electrostatic contour maps for CoMFA and CoMSIA (Figure 5C,D) are concordant. In both cases, a red polyhedron can be seen through the piperazine and indole connecting chain. In the case of CoMSIA, the red polyhedron is closer to the piperazine nitrogen atom 1 (N-1) (Figure 5D). Therefore, the insertion of groups favoring a higher charge density over this nitrogen atom would be favorable. This implies that increasing the basicity of such nitrogen atom would be favorable, which is consistent with our docking studies for the most active derivative **4d**, where the nitrogen atom (N-1) of piperazine moiety mediates a key salt bridge with Asp106 (3.32). A red polyhedron over the hydroxyl group on CoMFA map (Figure 5C) indicates that the presence of an electronegative atom in that position would be beneficial. Another red polyhedron is seen close to the halogen of the indole ring. Complementary to this information, the CoMSIA electrostatic contour map (Figure 5D) shows a blue polyhedron in the indole ring. Therefore, the presence of electron-attracting groups on the indole scaffold would be beneficial for biological activity. Among them, it would be interesting to explore groups NO_2_, CF_3_, CN and COR among others. In Figure 6, we summarize the structure–activity relationships found in this study. With this information, new active molecules can be designed and synthesized in the future.

## 3. Materials and Methods

All organic solvents used for the synthesis were of analytical grade. All reagents used were purchased from Sigma-Aldrich (St. Louis, MO, USA), Merck (Kenilworth, NJ, USA) or AK Scientific (Union City, CA, USA) and were used as received. Melting points were determined on a Stuart Scientific SMP30 apparatus (Bibby Scientific Limited, Staffordshire, UK) and are uncorrected. NMR spectra were recorded on a Bruker Avance III HD 400 (Billerica, MA, USA) at 400.1 MHz for ^1^H, 100.6 MHz for ^13^C-NMR and 376.5 MHz for ^19^F-NMR using the solvent signal (CDCl_3_ or DMSO-d_6_) as reference. The chemical shifts are expressed in ppm (δ scale) downfield from tetramethylsilane (TMS). Multiplicity is given as follows: s, singlet; bs, broad singlet; d, doublet; t, triplet; td, triplet of doublets; m, multiplet. Coupling constants values (*J*) are given in Hertz. Atom numbering and, ^1^H and ^13^C NMR spectra of the final compounds are available in the ESI. High resolution mass spectra were obtained on mass spectrometer with flight time analyzer (TOF) and Triwave^®^ system model SYNAPT™ G2 (WATERS, Milford, MA, USA), using atmospheric pressure ionization with electro spray (ESI+/−), Capillarity 3.0, source temperature 100 °C, desolvation temperature 500 °C. The IR spectra were obtained on a Bruker Vector 22 spectrophotometer (Billerica, MA, USA) using KBr discs. Column chromatography was performed on Merck silica gel 60 (70–230 mesh). Thin layer chromatographic separations were performed on Merck silica gel 60 (70–230 mesh) chromatofoils.

### 3.1. Synthetic Procedures

*2-bromo-1-(5-fluoro-1H-indol-3-yl)ethan-1-one* (**1a**)

To a solution of 5-fluoroindole (1 g, 7.40 mmol) in dry CH_2_Cl_2_ (30 mL) was added anhydrous zinc chloride (2.08 g, 15.25 mmol) under N_2_ atmosphere; immediately, methylmagnesium bromide 3 M (2.5 mL, 7.50 mmol) was slowly added over a 20 min period and the mixture was vigorously stirred for 2 h at room temperature. After this time, bromoacetyl chloride (0.84 mL, 9.65 mmol) was added in one portion and the mixture was stirred until the starting product disappeared upon checking TLC. The reaction was quenched by adding saturated ammonium chloride solution and extracted with CH_2_Cl_2_. The combined organic layers were dried with anhydrous sodium sulfate and the removal of the solvent afforded a residue, which was further purified by column chromatography on silica gel (CH_2_Cl_2_/ EtOAc 5:1) to give 929 mg of (**1a**) as a light brown solid. Yield: 49% mp: 200.5–200.9 °C; IR (KBr) cm^−1^: 3196, 2955, 1645, 1519, 1467 and 1168. ^1^H-NMR (400.1 MHz, DMSO-d_6_) δ (ppm): 12.28 (s, 1H, N-H), 8.54 (d, *J* = 3.1 Hz, 1H, H-2′), 7.83 (dd, *J* = 2.2 and 9.8 Hz, 1H, H-4′), 7.53 (dd, *J* = 4,6 and 8,8 Hz, 1H, H-7′), 7,10 (td, *J* = 2.4 and 9.3 Hz, 1H, H-6′), 4.65 (s, 2H, H-2). ^13^C-NMR (100.6 MHz, DMSO-d_6_) δ (ppm): 186.8, 159.2 (d, *J* = 235.1 Hz); 137.0, 133.7, 126.5 (d, *J* = 11.1 Hz), 114.1, 114.0, 111.8 (d, *J* = 26.0 Hz), 106.5 (d, *J* = 26.0 Hz), 33.7. HRMS calculated for C_10_H_8_BrFNO [M + H^+^]: 255.9768; Found: 255.9764.

*2-bromo-1-(5-methoxy-1H-indol-3-yl)ethan-1-one* (**1b**)

5-Methoxyindole (1.01 g, 6.80 mmol), anhydrous zinc chloride (1.85 g, 13.6 mmol), methylmagnesium bromide 3M (5 mL, 15.0 mmol) and bromoacetyl chloride (0.80 mL, 9.19 mmol) were reacted to give a residue, which was purified by column chromatography on silica gel using CH_2_Cl_2_/EtOAc 5:1 to afford 1.21 g of (**1b**) as a pale pink solid. Yield: 66% mp: 230–233 °C; IR (KBr) cm^−1^: 3193, 1643. ^1^H-NMR (400.1 MHz, DMSO-d_6_) δ (ppm): 12.07 (s, 1H, N-H); 8.38 (d, *J* = 3.2 Hz, 1H, H-2′); 7.67 (d, *J* = 2.0 Hz, 1H, H-4′); 7.40 (d, J = 8.8 Hz, 1H, H-7′); 6.88 (dd, *J* = 8.8 and 2.3 Hz,1H, H-6′); 4.85 (s, 2H, H-2); 3.79 (s, 3H, OCH_3_). ^13^C-NMR (100.6 MHz, DMSO-d_6_) δ (ppm): 186.4, 156.1, 135.2, 131.8, 126.6, 113.8, 113.5, 113.3, 103.3, 55.7 and 46.6. HRMS calculated for C_11_H_11_BrNO_2_ [M + H^+^]: 267.9968; Found: 267.9968.

*General Procedure for 2-Bromo-1-(arylsulfonyl-1H-3-yl)ethanone Derivatives* (**2a**–**i**):

*2-bromo-1-(5-fluoro-1-((4-iodophenyl)sulfonyl)-1H-indol-3-yl)ethan-1-one* (**2a**)

In a round bottom flask under N_2_, 2-bromo-1-(5-fluoro-1*H*-indol-3-yl)ethan-1-one (**1a**) (2.0 g, 7.81 mmol), *p*-iodobenzenesulfonyl chloride (2.84 g, 9.39 mmol), DMAP (96 mg, 0.78 mmol), and triethylamine (1.6 mL, 11.53 mmol) were dissolved in 30 mL of dry CH_2_Cl_2_, and the solution was stirred at room temperature until the starting material disappeared upon checking TLC. The reaction mixture was quenched by dilution with CH_2_Cl_2_ (30 mL), and the organic extract was washed with 1N HCl (20 mL). The organic layer was dried over anhydrous Na_2_SO_4_ and filtered, and the solvent was removed under vacuum. The product was purified by silica gel column chromatography using CH_2_Cl_2_/hexane (2:1) to give 2.08 g of (**2a**) as light pink crystalline plates. Yield: 51% m.p.: 165.8–166.4 °C; IR (KBr) cm^−1^: 1683, 1378, 1192. ^1^H-NMR (400.1 MHz, DMSO-d_6_) δ (ppm): 8.24 (s, 1H, H-2′), 7.93 (dd, *J* = 8.9 and 2.5 Hz, 1H, H-4′), 7.80 (m, 3H, H-7′, H-3″ and H-5″), 7,56 (d, *J* = 8.5 Hz, 2H, H-2″ and H-6″), 7.08 (td, *J* = 8.9 and 2.6 Hz, 1H, H-6′), 4.45 (s, 2H, H-2). ^13^C-NMR (100.6 MHz, DMSO-d_6_) δ (ppm): 185.6, 159.8 (d, *J* = 243.8 Hz), 138.1, 135.6, 132.3, 129.9, 127.8, 127.1, 113.8, 113.6, 113.2 (d, *J* = 9.5 Hz), 108.1 (d, *J* = 25.6 Hz), 102.3 and 44.7. HRMS calculated for C_16_H_11_BrFINO_3_S [M + H^+^]: 521.8666; Found: 521.8673.

*2-bromo-1-(5-fluoro-1-(naphthalen-1-ylsulfonyl)-1H-indol-3-yl)ethan-1-one* (**2b**)

2-Bromo-1-(5-fluoro-1*H*-indol-3-yl)ethan-1-one (**1a**) (920 mg, 3.60 mmol), naphthalenesulfonyl chloride (947 mg, 4.32 mmol), DMAP (44 mg, 0.36 mmol) and triethylamine (0.8 mL, 5.77 mmol) were reacted to give a crude mixture, which was purified by column chromatography on silica gel using CH_2_Cl_2_/hexane 2:1 to afford 675 mg of (**2b**) as pale pink crystalline plates. Yield: 42% m.p.: 176.5–177.7 °C; IR (KBr) cm^−1^: 1676, 1375, 1159. ^1^H-NMR (400.1 MHz, CDCl_3_) δ (ppm): 8.53 (d, *J* = 8.0 Hz, 1H, H-2″), 8.53 (s, 1H, H-2′), 8.31 (dd, *J* = 7.5 and 1.0 Hz, 1H, H-8″), 8.06 (d, *J* = 8.0 Hz, 1H, H-4′), 7.83–7.88 (m, 2H, H-4″ and H-5″), 7.63–7.57 (m, 2H, H-3″ and H-7′), 7.55–7.48 (m, 2H, H-6″ and H-7″), 6.96 (td, *J* = 9.0 and 2.5 Hz, 1H, H-6′), 4.47 (s, 2H, H-2). ^13^C-NMR (100.6 MHz, CDCl_3_) δ (ppm): 187.1, 161.0 (d, *J* = 244.6 Hz, 137.2, 134.7, 134.5, 132.5, 131.7, 130.0, 129.9, 129.0 (d, *J* = 11.1 Hz), 128.2, 128.1, 124.5, 123.5, 117.6 (d, *J* = 4.1 Hz), 114.8, 114.6, 114.5 (d, *J* = 9.4 Hz), 109.3 (d, *J* = 25.4 Hz) and 46.1. HRMS calculated for C_20_H_14_BrFNO_3_S [M + H^+^]: 445.9856; Found: 445.9854.

*2-bromo-1-(5-fluoro-1-((4-methoxyphenyl)sulfonyl)-1H-indol-3-yl)ethan-1-one* (**2c**)

2-Bromo-1-(5-fluoro-1*H*-indol-3-yl)ethan-1-one (**1a**) (1.44 g, 5.63 mmol), *p*-methoxybenzenesulfonyl chloride (1.40 g, 6.76 mmol), DMAP (69 mg, 0.57 mmol) and triethylamine (1.2 mL, 8.65 mmol) were reacted to give a crude mixture, which was purified by column chromatography on silica gel using CH_2_Cl_2_/hexane 2:1 to afford 897 mg of (**2c**) as orange crystalline plates. Yield: 37% m.p.: 190.3–194.1 °C; IR (KBr) cm^−1^: 1672, 1379 and 1163. ^1^H-NMR (400.1 MHz, CDCl_3_) δ (ppm): 8.85 (s, 1H, H-2′), 7.96 (d, *J* = 9.0 Hz, 2H, H-2″ and H-6″), 7.88 (dd, *J* =9.1 and 4.3, 1H, H-4′), 7.83 (dd, *J* = 9.1 and 2.6 Hz, 1H, H-7′), 7.12 (td, *J* = 9.0 and 2.6 Hz, 1H, H-6′), 7.00 (d, 9.0 Hz, 2H, H-3″ and H-5″), 4.89 (s, 2H, H-2), 3.78 (s, 3H, OCH_3_). ^13^C-NMR (100.6 MHz, CDCl_3_) δ (ppm): 187.4, 164.7, 160.3 (d, *J* = 241.1 Hz), 135.8, 130.9, 130.0, 128.6 (d, *J* = 11.0 Hz), 127.9, 117.6, 115.3, 114.7 (d, *J* = 9.4 Hz), 114.0 (d, *J* = 25.7 Hz), 108.2 (d, *J* = 25.3 Hz), 56.1, 46.8. HRMS calculated for C_17_H_14_BrFNO_4_S [M + H^+^]: 425.9805; Found: 425.9798.

*2-bromo-1-(1-((5-bromothiophen-2-yl)sulfonyl)-5-fluoro-1H-indol-3-yl)ethan-1-one* (**2d**)

2-Bromo-1-(5-fluoro-1*H*-indol-3-yl)ethan-1-one (**1a**) (1 g, 3.91 mmol), 5-bromothiophene-2-sulfonyl chloride (1.23 g, 4.69 mmol), DMAP (48 mg, 0.39 mmol) and triethylamine (0.8 mL, 5.77 mmol) were reacted to give a crude mixture, which was purified by column chromatography on silica gel using CH_2_Cl_2_/hexane (2:1) to afford 549 mg of (**2d**) as pale pink crystalline plates. Yield: 29%; m.p.: 171.0–174.6 °C; IR (KBr) cm^−1^: 1676, 1384, 1160, 1534, 1471, 1198. ^1^H-NMR (400.1 MHz, CDCl_3_) δ (ppm): 8.20 (s, 1H, H-2′), 7.94 (dd, *J* = 8.9 and 2.5 Hz, 1H, H-4′), 7.81 (dd, *J* = 9.1 and 4.3 Hz, 1H, H-7′), 7.48 (d, *J* = 4.1 Hz, 1H, H-2″), 7.11 (td, *J* = 8.9 and 2.6 Hz, 1H, H-6′); 6.99 (d, *J* = 4.1 Hz, 1H, H-3″), 4.47 (s, 2H, H-2). ^13^C-NMR (100.6 MHz, CDCl_3_) δ (ppm): 186.7, 160.9 (d, *J* = 243.4 Hz), 137.2, 134.8, 133.1, 130.9 (d, *J* = 16.8 Hz), 128.9 (d, *J* = 11.0 Hz), 124.1, 118.6 (d, *J* = 4.2 Hz), 115.0, 114.4, 114.3 (d, *J* = 9.5 Hz), 109.2 (d, *J* = 25.7 Hz), 45.8. HRMS calculated for C_14_H_9_Br_2_FNO_3_S_2_ [M + H^+^]: 479.8369; Found: 479.8363.

*2-bromo-1-(1-((4-iodophenyl)sulfonyl)-5-methoxy-1H-indol-3-yl)ethan-1-one* (**2e**)

2-Bromo-1-(5-methoxy-1*H*-indol-3-yl)ethan-1-one (**1b**) (1.06 g, 3.7 mmol), *p*-iodobenzenesulfonyl chloride (1.12 g, 4.1 mmol), DMAP (46 mg, 0.38 mmol) and triethylamine (0.6 mL, 4.32 mmol) were reacted to give a crude mixture, which was purified by column chromatography on silica gel using CH_2_Cl_2_ to afford 796 mg of (**2e**) as colorless crystalline plates. Yield: 40%; m.p.: 189.0–190.0 °C; IR (KBr) cm^−1^: 1681, 1378, 1132, 1053, 607, 586. ^1^H-NMR (400.1 MHz, CDCl_3_) δ (ppm): 8.44 (s, 1H, H-2′), 8.06 (d, *J* = 8.1 Hz; 2H, H-3″ and H-5″), 8.00–7.98 (m, 2H, H-4′ and H-7′), 7.22 (d, *J* = 8.1 Hz, 2H, H-2″ and H-6″), 7.01 (d, *J* = 9.1 Hz, 1H, H-6′), 4.77 (s, 2H, H-2), 3.04 (s, 3H, OCH_3_). ^13^C-NMR (100.6 MHz, CDCl_3_) δ (ppm): 186.9, 157.9, 139.0 (2C), 136.8, 132.3, 129.1, 128.7, 128.2 (2C), 118.4, 116.2, 113.9, 104.6, 102.9, 55.7, 45.9. HRMS calculated for C_17_H_14_BrINO_4_S [M + H^+^]: 533.8866; Found: 533.8862.

*2-bromo-1-(5-methoxy-1-(naphthalen-1-ylsulfonyl)-1H-indol-3-yl)ethan-1-one* (**2f**)

2-Bromo-1-(5-methoxy-1*H*-indol-3-yl)ethan-1-one (**1b**) (2.00 g, 7.5 mmol), naphthalenesulfonyl chloride (1.87 g, 8.2 mmol), DMAP (100 mg, 0.82 mmol) and triethylamine (1.1 mL, 7.93 mmol) were reacted to give a crude mixture, which was purified by column chromatography on silica gel using CH_2_Cl_2_ to afford 1.30 g of (**2f**) as a colorless crystalline plate. Yield: 38%; m.p.: 135.0–137.0 °C; IR (KBr) cm^−1^: 1656, 1372, 1165, 1027, 689. ^1^H-NMR (400.1 MHz, CDCl_3_) δ (ppm): 8.54 (d; *J* = 8.5 Hz, 1H, H-2″), 8.44 (s, 1H, H-2′), 8.26 (dd, *J* = 7.5 and 1.1 Hz, 1H, H-8″), 8.00 (d, *J* = 8.3 Hz, 1H, H-4″), 7.79 (d, *J* = 8.1 Hz, 1H, H-5″), 7.65 (d, *J* = 2.6 Hz, 1H, H-4′), 7.59–7.53 (m, 1H, H-3″), 7.53 (d, *J* = 9.2 Hz, 1H, H-7′), 7.47 (t, *J* = 7.81, 2H, H-7″ and H-6″), 6.81 (dd, *J* = 9.1 and 2.6 Hz, 1H, H-6′), 4.48 (s, 2H, H-2), 3.70 (s, 3H, OCH_3_). ^13^C-NMR (100.6 MHz, CDCl_3_) δ (ppm): 187.5, 158.5, 136.9, 134.7, 133.6, 132.8, 130.9, 129.9, 129.7, 129.5, 128.9, 128.3, 127.9, 124.5, 123.6, 117.7, 116.3, 114.2, 104.9, 56.1, and 46.3. HRMS calculated for C_21_H_17_BrNO_4_S [M + H^+^]: 458.0056; Found: 458.0063.

*2-bromo-1-(5-methoxy-1-((4-methoxyphenyl)sulfonyl)-1H-indol-3-yl)ethan-1-one* (**2g**)

2-Bromo-1-(5-methoxy-1H-indol-3-yl)ethan-1-one (**1b**) (2.00 g, 7.5 mmol), p-methoxybenzenesulfonyl chloride (1.69 g, 8.2 mmol), DMAP (91 mg, 0.7 mmol) and triethylamine (1.2 mL, 8.65 mmol) were reacted to give a crude mixture, which was purified by column chromatography on silica gel using CH_2_Cl_2_ to afford 1.14 g of (**2g**) as pale brown crystalline plates. Yield: 35% m.p.: 173–176 °C; IR (KBr) cm−1: 1673, 1375 1166, 994 and 680. ^1^H-NMR (400.1 MHz, CDCl_3_) δ (ppm): 8.18 (s, 1H, H-2′), 7.78 (d, *J* = 9.0 Hz, 2H, H-2″), 7.72 (d, *J* = 9.1 Hz, 1H, H-7′), 7.69 (d, *J* = 2.6 Hz, 1H, H-4′), 6.91 (dd, *J* = 9.1 and 2.6 Hz, 1H, H-6′), 6.84 (d, *J* = 9.0 Hz, 2H, H-3″), 4.49 (s, 2H, H-2), 3.75 (s, 3H, 4″OCH_3_), 3.72 (s, 3H, 5′-OCH_3_). ^13^C-NMR (100.6 MHz, CDCl_3_) δ (ppm): 187.0, 164.5, 157.8, 132.6, 129.5 (2C), 129.2, 128.6, 128.4, 117.8, 115.9, 114.9 (2C), 104.4, 108.2, 55.8, 55.7, 45.9. HRMS calculated for C_18_H_17_BrNO_5_S [M + H^+^]: 438.0005; Found: 438.0012.

*2-bromo-1-(1-((5-bromothiophen-2-yl)sulfonyl)-5-methoxy-1H-indol-3-yl)ethan-1-one* (**2h**)

2-Bromo-1-(5-methoxy-1*H*-indol-3-yl)ethan-1-one (**1b**) (632 mg, 2.4 mmol), 5-bromothiophene-2-sulfonyl chloride (1.07 g, 2.6 mmol), DMAP (30 mg, 0.25 mmol) and triethylamine (0.4 mL, 2.88 mmol) were reacted to give a crude mixture, which was purified by column chromatography on silica gel using CH_2_Cl_2_ to afford 312 mg of (**2h**) as a white crystalline plate. Yield: 30%; m.p.: 151.0–152.0 °C; IR (KBr) cm^−1^: 1656, 1390, 1169, 854, 688, 618. ^1^H-NMR (400.1 MHz, CDCl_3_) δ (ppm): 8.11 (s, 1H, H-2′), 7.73 (d, *J* = 9.0 Hz, 1H, H-7′), 7.72 (d, *J* = 2.7 Hz, 1H, H-4′), 7.46 (d, *J* = 4.1 Hz, 1H, H-3″), 6.99–6.94 (m, 2H, H-2″ and H-6′), 4.49 (s, 2H, H-2), 3.79 (s, 3H, 5′-OCH3). ^13^C-NMR (100.6 MHz, CDCl_3_) δ (ppm): 187.0, 158.1, 137.5, 134.6, 132.1, 131.0, 128.9, 128.8, 123.7, 118.8, 116.3, 114.0, 104.8, 55.8, and 45.9. HRMS calculated for C_15_H_12_Br_2_NO_4_S_2_ [M + H^+^]: 491.8569; Found: 491.8569.

*2-bromo-1-(5-methoxy-1-tosyl-1H-indol-3-yl)ethan-1-one* (**2i**)

2-Bromo-1-(5-methoxy-1*H*-indol-3-yl)ethan-1-one (**1b**) (623 mg, 2.3 mmol), *p*-toluensulfonyl chloride (687 mg, 3.4 mmol), DMAP (46 mg, 0.38 mmol) and triethylamine (0.35 mL, 2.52 mmol) were reacted to give a crude mixture, which was purified by column chromatography on silica gel using CH_2_Cl_2_-hexane (1:1) to afford 676 mg of (**2i**) as a white crystalline plate. Yield: 70%; m.p.: 182.0–183.0 °C; IR (KBr) cm^−1^: 1680, 1376, 1175, 891, 678. ^1^H-NMR (400.1 MHz, CDCl_3_) δ (ppm): 8.21 (s, 1H, H-2′), 7.74–7.67 (m, 4H, H-4′ and H-7′, H2″), 7.18 (d, *J* = 8.0 Hz, 2H, H-3″), 6.90 (d, *J* = 9.0 Hz, 1H, H-6′), 4.27 (s, 2H, H-2), 3.74 (s, 3H, 5′-OCH_3_), 2.26 (s, 3H, 4″-CH_3_). ^13^C-NMR (100.6 MHz, CDCl_3_) δ (ppm): 187.5, 158.2, 146.5, 134.7, 133.5, 130.7(2C), 129.7, 129.1, 127.6 (2C), 118.3, 116.4, 114.4, 104.9, 56.1, 31.9, 22.1. HRMS calculated for C_18_H_17_BrNO_4_S [M + H^+^]: 422.0056; Found: 422.0057.

*General Procedure for 2-(4-(Aryl)piperazin-1-yl)-1-(1-arylsulfonyl-1H-indol-3-yl)ethanone Derivatives* (**3a**–**v**):

*1-(5-fluoro-1-((4-iodophenyl)sulfonyl)-1H-indol-3-yl)-2-(4-(2-methoxyphenyl)piperazin-1-yl)ethan-1-one* (**3a**)

To a solution of 1-(2-methoxyphenyl)-piperazine (208 mg, 1.08 mmol) and potassium carbonate (149 mg, 1.08 mmol) in acetone (30 mL) 2-bromo-1-(5-fluoro-1-((4-iodophenyl)sulfonyl)-1H-indol-3-yl)ethan-1-one (**2a**) (511 mg, 0.98 mmol) was added and the mixture was stirred for 24 h at room temperature. The reaction was stopped by dilution with water (30 mL) and the organic layer was extracted with CH_2_Cl_2_ (3 × 30 mL). The combined organic layers were dried with anhydrous sodium sulfate, and removal of the solvent under vacuum afforded a crude residue. The solid was purified by column chromatography on silica gel (CH_2_Cl_2_) to yield 240 mg of (**3a**) as an orange gel. Yield: 39% m.p.: 155 °C (dec); IR (KBr) cm^−1^: 1656, 1562, 1500, 1385, 1174, 1142, 1028, 736. ^1^H-NMR (400.1 MHz, CDCl_3_) δ (ppm): 8.77 (s, 1H, H-2′), 8.04 (dd, *J* = 9.1 and 2.6 Hz, 1H, H-4′), 7.87 (dd, *J* = 9.1 and 4.3 Hz, 1H, H-7′), 7.83 (d, *J* = 8.5 Hz, 2H, H-3″ and H-5″), 7.62 (d, *J* = 8.6 Hz, 2H, H-2″ and H-6″), 7.11 (td, *J* = 8.9 and 2.6 Hz, 1H, H-6′), 7.05–7.00 (m, 1H, H-5‴), 6.96 (d, *J* = 4.1 Hz, 2H, H-3‴ and H-4‴), 6.88 (d, *J* = 7.8 Hz, 1H, H-6‴), 3.87 (s, 3H, OCH_3_), 3.68 (s, 2H, H-2), 3.14 (bs, 4H, H-3⁗ and H-5⁗), 2.80 (bs, 4H, H-2⁗ and H-6⁗). ^13^C-NMR (100.6 MHz, CDCl_3_) δ (ppm): 193.7, 161.1 (d, *J* = 242.5 Hz), 152.7, 141.5, 139.5, 137.5, 134.5, 131.1, 129.7 (d, *J* = 11.0 Hz), 128.5, 123.6, 121.5, 120.0 (d, *J* = 4.0 Hz), 118.6, 114.6 (d, *J* = 14.4 Hz), 114.4 (d, *J* = 1.5 Hz), 111.8, 109.6 (d, *J* = 25.5 Hz), 103.4, 67.5, 55.8, 54.2 (2C) and 51.1 (2C). HRMS calculated for C_27_H_26_FIN_3_O_4_S [M + H^+^]: 634.0667; Found: 634.0681.

*1-(5-fluoro-1-((4-iodophenyl)sulfonyl)-1H-indol-3-yl)-2-(4-(pyridin-2-yl)piperazin-1-yl)ethan-1-one* (**3b**)

1-(2-Pyridyl)-piperazine (406 mg, 2.49 mmol), potassium carbonate (344 mg, 2.49 mmol) and 2-bromo-1-(5-fluoro-1-((4-iodophenyl)sulfonyl)-1*H*-indol-3-yl)ethan-1-one (**2a**) (1.18 g, 2.26 mmol) were reacted to give a solid, which was purified by column chromatography on silica gel using CH_2_Cl_2_/ethyl acetate 5:1 to obtain 615 mg of pure product (**3b**) as white crystalline plates. Yield: 45% m.p.: 133.1–135.7 °C; IR (KBr) cm^−1^: 1664, 1387 and 1146, 1595, 1538, 1171, 737. ^1^H-NMR (400.1 MHz, CDCl_3_) δ (ppm): 8.75 (s, 1H, H-2′), 8.23 (d, *J* = 4.3 Hz, 1H, H-3‴), 8.05 (dd, *J* = 9.0 and 2.3 Hz, 1H, H-4′), 7.89–7.87 (m, 1H, H-7′), 7.85 (d, *J* = 8.6 Hz, 2H, H-3″ and H-5″), 7.62 (d, *J* = 8.4 Hz, 2H, H-2″ and H-6″), 7.52 (t, *J* = 7.7 Hz, 1H, H-5‴), 7.13 (td, *J* = 8.9 and 2.4 Hz, 1H, H-6′), 6.69–6.65 (m, 2H, H-4‴ and H-6‴), 3.69 (s, 2H, H-2), 3.64–3.57 (m, 4H, H-3⁗ and H-5⁗), 2.75–2.69 (m, 4H, H-2⁗ and H-6⁗). ^13^C-NMR (100.6 MHz, CDCl_3_) δ (ppm): 192.9, 160.7 (d, *J* = 242.7 Hz), 159.4, 148.0, 139.1 (2C), 137.6, 136.9, 134.0, 130.7, 129.2 (d, *J* = 11.1 Hz), 128.1 (2C), 119.5 (d, *J* = 4.2 Hz), 114.2 (d, *J* = 21.4 Hz), 114.0 (d, *J* = 4.9 Hz), 113.6, 109.2 (d, *J* = 25.5 Hz), 107.2, 103.1, 66.8, 53.3 (2C), 45.3 (2C). HRMS calculated for C_25_H_23_FIN_4_O_3_S [M + H^+^]: 605.0514; Found: 605.0522.

*1-(5-fluoro-1-((4-iodophenyl)sulfonyl)-1H-indol-3-yl)-2-morpholinoethan-1-one* (**3c**)

Morpholine (0.1 mL, 1.16 mmol), potassium carbonate (100 mg, 0.72 mmol) and 2-bromo-1-(5-fluoro-1-((4-iodophenyl)sulfonyl)-1*H*-indol-3-yl)ethan-1-one (**2a**) (340 mg, 0.65 mmol) were reacted to give an orange gel, which was purified by column chromatography on silica gel using CH_2_Cl_2_/ethyl acetate 5:1 to obtain 190 mg of pure product (**3c**) as orange crystalline plates. Yield: 55%; m.p.: 139.7–141.1 °C; IR (KBr) cm^−1^: 1655, 1568, 1535, 1389, 1174, 1144, 738. ^1^H-NMR (400.1 MHz, CDCl_3_) δ (ppm): 8.59 (s, 1H, H-2′); 7.93 (dd, *J* = 9.1 and 2.6 Hz, 1H, H-4′); 7.82–7.75 (m, 3H, H-7′, H-3″ and H-5″); 7.56 (d, *J* = 9.0 Hz, 2H, H-2″ and H-6″); 7.03 (td, *J* = 8.9 and 2.6 Hz, 1H, H-6′); 3.75–3.67 (m, 4H, H-3⁗ and H-5⁗); 3.58 (s, 2H, H-2); 2.59–2.52 (m, 4H, H-2⁗ and H-6⁗). ^13^C-NMR (100.6 MHz, CDCl_3_) δ (ppm): 192.5, 160.7 (d, *J* = 242.7 Hz), 139.1 (2C), 136.9, 133.7, 130.7, 129.1 (d, *J* = 11.0 Hz), 128.1 (2C), 119.5 (d, *J* = 4.2 Hz), 114.2 (d, *J* = 36.5 Hz), 114.1, 109.1 (d, *J* = 25.5 Hz), 103.1, 66.8 (2C), 66.7 and 53.8 (2C). HRMS calculated for C_20_H_19_FIN_2_O_4_S [M + H^+^]: 529.0089; Found: 529.0113.

*1-(5-fluoro-1-(naphthalen-1-ylsulfonyl)-1H-indol-3-yl)-2-(4-(2-methoxyphenyl)piperazin-1-yl)ethan-1-one* (**3d**)

1-(2-Methoxyphenyl)-piperazine (428 mg, 2.22 mmol), potassium carbonate (307 mg, 2.22 mmol) and 2-bromo-1-(5-fluoro-1-(naphthalen-1-ylsulfonyl)-1*H*-indol-3-yl)ethan-1-one (**2b**) (900 mg, 2.02 mmol) were reacted to give an orange gel, which was purified by column chromatography on silica gel using CH_2_Cl_2_/ethyl acetate 5:1 to obtain 1.04 g of pure product (**3d**) as a yellowish gel. Yield: 93% m.p.: product in gel state °C; IR (KBr) cm^−1^: 1655, 1593, 1502, 1373, 1174, 1146. ^1^H-NMR (400.1 MHz, CDCl_3_) δ (ppm): 8.93 (s, 1H, H-2′); 8.56 (d, *J* = 8.6 Hz, 1H, H-2″); 8.28 (d, *J* = 6.8, 1H, H-8″); 8.02 (d, *J* = 8.2 Hz, 1H, H-4′); 7.94 (dd, *J* = 9.1 and 2.5 Hz, 1H, H-4″); 7.82 (d, *J* = 8.1 Hz, 1H, H-5″); 7.66 (dd, *J* = 9.1 and 4.3 Hz, 1H, H-7′); 6.98–6.93 (m, 2H, H-6′ and H-5‴); 6.88 (d, *J* = 4.2 Hz, 2H, H-3‴ and H-4‴); 6.81 (d, *J* = 7.9 Hz, 1H, H-6‴); 3.79 (s, 3H, OCH_3_); 3.65 (s, 2H, H-2); 3.07 (s, 4H, H-3⁗ and H-5⁗); 2.76 (s, 4H, H-2⁗ and H-6⁗). ^13^C-NMR (100.6 MHz, CDCl_3_) δ (ppm): 192.9, 160.5 (d, *J* = 242.0 Hz), 152.3, 141.0, 136.7, 134.7, 134.4, 132.6, 130.8, 130.5, 129.6, 129.4, 128.9 (d, *J* = 11.2 Hz), 128.0, 127.6, 124.2, 124.1, 123.3 (d, *J* = 3.8 Hz), 121.0, 118.6 (d, *J* = 4.1 Hz), 118.4, 114.0 (d, *J* = 8.7 Hz), 113.7, 111.4, 109.0 (d, *J* = 25.5 Hz), 66.8, 55.4, 53.8 (2C) and 50.4 (2C). HRMS calculated for C_31_H_29_FN_3_O_4_S [M + H^+^]: 558.1857; Found: 558.1885.

*1-(5-fluoro-1-(naphthalen-1-ylsulfonyl)-1H-indol-3-yl)-2-(4-(pyridin-2-yl)piperazin-1-yl)ethan-1-one* (**3e**)

1-(2-Pyridyl)-piperazine (315 mg, 1.93 mmol), potassium carbonate (267 mg, 1.93 mmol) and 2-bromo-1-(5-fluoro-1-(naphthalen-1-ylsulfonyl)-1*H*-indol-3-yl)ethan-1-one (**2b**) (780 mg, 1.75 mmol) were reacted to give a solid, which was purified by column chromatography on silica gel using CH_2_Cl_2_/ethyl acetate 5:1 to obtain 528 mg of pure product (**3e**) as white crystalline plates. Yield: 57% m.p.: 173–175.7 °C; IR (KBr) cm^−1^: 1656, 1596, 1531, 1382, 1199, 1170. ^1^H-NMR (400.1 MHz, CDCl_3_) δ (ppm): 9.02 (s, 1H, H-2′), 8.64 (d, *J* = 8.7 Hz, 1H, H-2″); 8.38 (d, *J* = 7.5 Hz, 1H, H-8″); 8.25 (d, *J* = 4.6 Hz, 1H, H-4′); 8.10–8.06 (m, 1H, H-3‴); 8.04 (dd, *J* = 9.2 and 2.4 Hz, 1H, H-4″); 7.88 (d, *J* = 8.2 Hz, 1H, H-5″); 7.79 (dd, *J* = 9.1 and 4.3 Hz, 1H, H-7′); 7.64–7.58 (m, 2H, H-3″ and H-5‴); 7.56–7.51 (m, 2H, H-6″ and H-7″); 7.06 (td, *J* = 8.9 and 2.3 Hz, 1H, H-6′); 6.70–6.66 (m, 2H, H-4‴ and H-6‴); 3.67 (s, 2H, H-2); 3.58–3.53 (m, 4H, H-3⁗ and H-5⁗); 2.72–2.67 (m, 4H, H-2⁗ and H-6⁗). ^13^C-NMR (100.6 MHz, CDCl_3_) δ (ppm): 193.1, 160.5 (d, *J* = 241.0 Hz, 1C), 159.4, 147.9, 137.6, 136.7, 134.6, 134.3, 132.5, 130.8, 130.5, 129.6, 129.4, 128.9 (d, *J* = 11.1 Hz), 127.9, 127.6, 124.2, 123.2, 118.6 (d, *J* = 4.2 Hz), 114.1 (d, *J* = 10.0 Hz), 113.9 (d, *J* = 26.0 Hz), 113.6, 109.0 (d, *J* = 25.4 Hz), 107.2, 67.1, 53.3 (2C) and 45.2 (2C). HRMS calculated for C_29_H_26_FN_4_O_3_S [M + H^+^]: 529.1704; Found: 529.1720.

*1-(5-fluoro-1-(naphthalen-1-ylsulfonyl)-1H-indol-3-yl)-2-morpholinoethan-1-one* (**3f**)

Morpholine (0.12 mL, 1.39 mmol), potassium carbonate (188 mg, 1.36 mmol) and 2-bromo-1-(5-fluoro-1-(naphthalen-1-ylsulfonyl)-1*H*-indol-3-yl)ethan-1-one (**2b**) (550 mg, 1.23 mmol) were reacted to obtain a crude mixture, which was purified by column chromatography employing CH_2_Cl_2_/ethyl acetate 5:1 to yield 436 mg of (**3f**) as a yellow gel. Yield: 78% m.p.: 145–148 °C; IR (KBr) cm^−1^: 1665, 1587, 1535, 1361, 1171, 1155. ^1^H-NMR (400.1 MHz, CDCl_3_) δ (ppm): 8.96 (s, 1H, H-2′); 8.66 (d, *J* = 8.6 Hz, 1H, H-2″); 8.37 (d, *J* = 7.5 Hz, 1H, H-8″); 8.16 (d, *J* = 8.2, 1H, H-5″); 8.01 (dd, *J* = 9.1 and 2.4 Hz, 1H, H-4′); 7.94 (d, *J* = 8.1 Hz, 1H, H-4″); 7.74 (dd, *J* = 9.1 and 4.3 Hz, 1H, H-7′); 7.70–7.65 (m, 1H, H-3″); 7.62 (dd, *J* = 10.9 and 4.7 Hz, 2H, H-6″ and H-7″); 7.04 (td, *J* = 9.0 and 2.0 Hz, 1H, H-6′); 3.79–3.75 (m, 4H, H-3⁗ and H-5⁗); 3.66 (s, 2H, H-2); 2.63 (s, 4H, H-2⁗ and H-6⁗). ^13^C-NMR (100.6 MHz, CDCl_3_) δ (ppm): 192.7, 160.5 (d, *J* = 242.0 Hz), 136.7, 134.4, 134.3 132.5, 130.8, 130.5, 129.6, 129.3, 128.8 (d, *J* = 11.0 Hz), 127.9, 127.6, 124.2, 123.2, 118.6 (d, *J* = 4.2 Hz), 114.0 (d, *J* = 1.8 Hz), 113.9 (d, *J* = 18.5 Hz), 108.9 (d, *J* = 25.5 Hz), 67.0, 66.8 (2C) and 53.8 (2C). HRMS calculated for C_24_H_22_FN_2_O_4_S [M + H^+^]: 453.1279; Found: 453.1312.

*1-(5-fluoro-1-(naphthalen-1-ylsulfonyl)-1H-indol-3-yl)-2-(4-(pyrimidin-2-yl)piperazin-1-yl)ethan-1-one* (**3g**)

1-(2-Pyrimidyl)-piperazine (146 mg, 0.89 mmol), potassium carbonate (123 mg, 0.89 mmol) and 2-bromo-1-(5-fluoro-1-(naphthalen-1-ylsulfonyl)-1*H*-indol-3-yl)ethan-1-one (**2b**) (363 mg, 0.81 mmol) were reacted to give a solid, which was purified by column chromatography on silica gel using CH_2_Cl_2_/ethyl acetate 5:1 to give 329 mg of (**3g**) as white crystalline plates. Yield: 77%; m.p.: 185.9–187.5 °C; IR (KBr) cm^−1^: 1659, 1585, 1544, 1359, 1169, 1147. ^1^H-NMR (400.1 MHz, CDCl_3_) δ (ppm): 9.02 (s, 1H, H-2′); 8.66 (d, *J* = 8.6 Hz, 1H, H-2″); 8.40–8.33 (m, 3H, H-3‴, H-5‴ and H-8″); 8.13 (d, *J* = 8.2 Hz, 1H, H-4′); 8.04 (dd, *J* = 9.1 and 1.9 Hz, 1H, H-4″); 7.92 (d, *J* = 8.1 Hz, 1H, H-5″); 7.76 (dd, *J* = 9.1 and 4.1 Hz, 1H, H-7′); 7.66 (t, *J* = 7.8 Hz, 1H, H-3″); 7.59 (m, 2H, H-6″ and H-7″); 7.06 (dt, *J* = 8.9 and 1.8 Hz, 1H, H-6′); 6.54 (s, 1H, H-4‴); 3.89 (s, 4H, H-3⁗ and H-5⁗); 3.69 (s, 2H, H-2); 2.67 (s, 4H, H-2⁗ and H-6⁗). ^13^C-NMR (100.6 MHz, CDCl_3_) δ (ppm): 193.3, 160.9 (d, *J* = 241 Hz), 162.1, 158.1, 137.0, 134.9, 134.7, 133.0, 131.2, 130.8, 130.0, 129.7, 129.3 (d, *J* = 10.9 Hz), 128.4, 128.0, 124.6, 123.6, 119.0 (d, *J* = 4.0 Hz), 114.4 (d, *J* = 8.3 Hz), 114.1, 110.5, 109.4 (d, *J* = 25.6 Hz), 67.4, 53.8 (2C) and 44.0 (2C). HRMS calculated for C_28_H_25_FN_5_O_3_S [M + H^+^]: 530.1657; Found: 530.1700.

*1-(5-fluoro-1-((4-methoxyphenyl)sulfonyl)-1H-indol-3-yl)-2-(4-(2-methoxyphenyl)piperazin-1-yl)ethan-1-one* (**3h**)

1-(2-Methoxyphenyl)-piperazine (448 mg, 2.32 mmol), potassium carbonate (321 mg, 2.32 mmol) and 2-bromo-1-(5-fluoro-1-((4-methoxyphenyl)sulfonyl)-1*H*-indol-3-yl)ethan-1-one (**2c**) (900 mg, 2.11 mmol) were reacted to give a gel, which was purified by column chromatography on silica gel using CH_2_Cl_2_/ethyl acetate 7:1 to obtain 1.10 g of (**3h**) as an orange gel. Yield: 97%; m.p.: product in gel state; IR (KBr) cm^−1^: 1665, 1594, 1500, 1380, 1165, 1198. ^1^H-NMR (400.1 MHz, CDCl_3_) δ (ppm): 8.70 (s, 1H, H-2′); 7.93 (dd, *J* = 9.2 and 2.5 Hz, 1H, H-4′); 7.81–7.74 (m, 3H, H-7′, H-2″ and H-6″); 6.97, (td, *J* = 9.0 and 2.5 Hz, 1H, H-6′); 6.93–6.83 (m, 2H, H-3‴ and H-5‴); 6.83–6.74 (m, 2H, H-3″, H-5″, H-4‴ and H-6‴); 3.75 (s, 3H, 4″-OCH_3_); 3.64 (s, 3H, 2‴-OCH_3_); 3.60 (s, 2H, H-2); 3.04 (s, 4H, H-3⁗ and H-5⁗); 2.70 (s, 4H, H-2⁗ and H-6⁗). ^13^C-NMR (100.6 MHz, CDCl_3_) δ (ppm): 193.3, 164.5, 160.5 (d, *J* = 241.5), 152.3, 141.1, 134.4, 130.8, 129.5, 129.1 (d, *J* = 11.0 Hz), 128.6, 123.1, 121.0, 118.9 (d, *J* = 4.2 Hz), 118.2, 115.0, 114.1 (d, *J* = 9.5 Hz), 113.8 (d, *J* = 25.9 Hz), 114.4, 108.8 (d, *J* = 25.4 Hz), 66.7, 55.7, 55.4, 53.7 (2C) and 50.6 (2C). HRMS calculated for C_28_H_29_FN_3_O_5_S [M + H^+^]: 538.1806; Found: 538.1825.

*1-(1-((5-bromothiophen-2-yl)sulfonyl)-5-fluoro-1H-indol-3-yl)-2-(4-(2-methoxyphenyl)piperazin-1-yl)ethan-1-one* (**3i**)

1-(2-Methoxyphenyl)-piperazine (432 mg, 2.24 mmol), potassium carbonate (310 mg, 2.24 mmol) and 2-bromo-1-(1-((5-bromothiophen-2-yl)sulfonyl)-5-fluoro-1*H*-indol-3-yl)ethan-1-one (**2d**) (985 mg, 2.04 mmol) were reacted to give a gel, which was purified by column chromatography on silica gel using CH_2_Cl_2_/ethyl acetate 5:1 to obtain 505 mg of (**3i**) as a light orange solid. Yield: 42%; m.p.: 104–107 °C; IR (KBr) cm^−1^: 1667, 1588, 1500, 1390, 1174, 1148. ^1^H-NMR (400.1 MHz, CDCl_3_) δ (ppm): 8.76 (s, 1H, H-2′); 8.07 (dd, *J* = 9.0 and 2.5 Hz, 1H, H-4′); 7.88 (dd, *J* = 9.1 and 4.3 Hz, 1H, H-7′); 7.54 (d, *J* = 4.0 Hz, 1H, H-3″); 7.14 (td, *J* = 8.9 and 2.5 Hz, 1H, H-6′); 7.03 (d, *J* = 4.6 Hz, 1H, H-2″); 6.99–6.86 (m, 4H, H-3‴, H-4‴, H-5‴ and H-6‴); 3.87 (s, 3H, 2‴-OCH_3_); 3.70 (s, 2H, H-2); 3.17 (s, 4H, H-3⁗ and H-5⁗); 2.81 (s, 4H, H-2⁗ and H-6⁗). ^13^C-NMR (100.6 MHz, CDCl_3_) δ (ppm): 193.2, 160.8 (d, *J* = 242.6 Hz), 152.3, 141.1, 137.6, 134.5, 134.0, 131.0, 130.5, 129.4 (d, *J* = 11.0 Hz), 123.6, 123.1, 121.1, 119.8 (d, *J* = 4.2 Hz), 118.3, 114.1 (d, *J* = 9.5 Hz), 113.8 (d, *J* = 25.9 Hz), 111.3, 109.3 (d, *J* = 25.5 Hz), 67.0, 55.4, 53.8 (2C), 50.6 (2C). HRMS calculated for C_25_H_24_BrFN_3_O_4_S_2_ [M + H^+^]: 592.0370; Found: 592.0399.

*1-(1-((4-iodophenyl)sulfonyl)-5-methoxy-1H-indol-3-yl)-2-(4-(2-methoxyphenyl)piperazin-1-yl)ethan-1-one* (**3j**)

1-(2-Methoxyphenyl)-piperazine (154 mg, 0.80 mmol), potassium carbonate (96 mg, 0.70 mmol) and 2-bromo-1-(1-((4-iodophenyl)sulfonyl)-5-methoxy-1*H*-indol-3-yl)ethan-1-one (**2e**) (370 mg, 0.69 mmol) were reacted to give a solid, which was purified by column chromatography on silica gel using CH_2_Cl_2_/AcOEt 5:1 to give 416 mg of (**3j**) as white crystalline plates. Yield: 93%; m.p.: 99–100 °C; IR (KBr) cm^−1^: 1663, 1386, 1215, 1176, 963, 585. ^1^H-NMR (400.1 MHz, CDCl_3_) δ (ppm): 8.57 (s, 1H, H-2′); 7.77 (d, *J* = 2.4 Hz, 1H, H-4′); 7.74 (d, *J* = 8.3, 2H, H-2″ and H-6″); 7.73 (d, *J* = 9.1 Hz, 1H, H-7′); 7.54 (d, *J* = 8.3 Hz, 2H, H-3″ and H-5″); 6.97–6.85 (m, 4H, H-6′, H-3‴, H-4‴ and H-5‴); 6.81 (d, *J* = 7.9 Hz, H-6‴); 3.80 (s, 3H, 5′-OCH_3_); 3.77 (s, 3H, 2‴-OCH_3_); 3.65 (s, 2H, H-2); 3.08 (bs, 4H, H-3⁗ and H-5⁗); 2.75 (bs, 4H, H-2⁗ and H-6⁗). ^13^C-NMR (100.6 MHz, CDCl_3_) δ (ppm): 193.4, 157.8, 152.3, 141.0, 138.9 (2C), 137.2, 133.1, 129.1, 128.9, 128.1(2C), 123.2, 121.1, 119.7, 118.3, 115.8, 113.8, 111.3, 104.7, 102.7, 66.6, 55.7, 55.4, 53.8(2C), 50.6(2C). HRMS calculated for C_28_H_29_IN_3_O_5_S [M + H^+^]: 646.0867; Found: 646.0869.

*1-(1-((4-iodophenyl)sulfonyl)-5-methoxy-1H-indol-3-yl)-2-morpholinoethan-1-one* (**3k**)

Morpholine (0.1 mL, 1.16 mmol), potassium carbonate (83 mg, 0.6 mmol) and 2-bromo-1-(1-((4-iodophenyl)sulfonyl)-5-methoxy-1H-indol-3-yl)ethan-1-one (**2e**) (320 mg, 0.6 mmol) were reacted to give a solid, which was purified by column chromatography on silica gel using CH_2_Cl_2_/ethyl acetate 5:1 to give 237 mg of (**3k**) as yellow crystalline plates. Yield: 73% m.p.: 163–166 °C; IR (KBr) cm^−1^: 1651, 1387, 1174, 1215, 1115, 863, 616. ^1^H-NMR (400.1 MHz, CDCl_3_) δ (ppm): 8.48 (s, 1H, H-2′); 7.76 (d, *J* = 8.5 Hz, 2H, H-2″ and H-6″); 7.74 (d, *J* = 2.6 Hz, 1H, H-4′); 7.71 (d; *J* = 9.1 Hz, 1H, H-7′); 7.53 (d, *J* = 8.3 Hz, 2H, H-3″ and H-5″); 6.90 (dd, *J* = 9.1 and 2.5 Hz, 1H, H-6′); 3.76 (s, 3H, 5′-OCH3); 3.70 (t, *J* = 4.6 Hz, 4H, H-3⁗ and H-5⁗); 3.57 (s, 2H, H-2); 2.53 (t, *J* = 4.4 Hz, 4H, H-2⁗ and H-6⁗).^13^C-NMR (100.6 MHz, CDCl_3_) δ (ppm): 193.0, 157.8, 138.9 (2C), 137.1, 132.7, 129.1, 128.9, 128.1 (2C), 119.7, 115.8, 113.8, 104.7, 102.8, 66.9 (2C), 66.7, 55.7, 53.8 (2C). HRMS calculated for C_21_H_22_IN_2_O_5_S [M + H^+^]: 541.0289; Found: 541.0305.

*1-(5-methoxy-1-(naphthalen-1-ylsulfonyl)-1H-indol-3-yl)-2-(4-(2-methoxyphenyl)piperazin-1-yl)ethan-1-one* (**3l**)

1-(2-Methoxyphenyl)-piperazine (119 mg, 0.62 mmol), potassium carbonate (86 mg, 0.62 mmol) and 2-bromo-1-(5-methoxy-1-(naphthalen-1-ylsulfonyl)-1*H*-indol-3-yl)ethan-1-one (**2f**) (284 mg, 0.62 mmol) were reacted to give a solid, which was purified by column chromatography on silica gel using CH_2_Cl_2_/ethyl acetate 5:1 to give 269 mg of product (**3l**) as a brown-orange solid. Yield: 77%; m.p.: 78–80 °C; IR (KBr) cm^−1^: 1661, 1371, 1134, 1215, 1030. ^1^H-NMR (400.1 MHz, CDCl_3_) δ (ppm): 8.92 (s, 1H, H-2′); 8.67 (d, *J* = 8.7 Hz, 1H, H-2″); 8.32 (dd, *J* = 7.5 and 1.0 Hz, 1H, H-8″); 8.08 (d, *J* = 8.3 Hz, 1H, H-4″); 7,88 (d, *J* = 7.8 Hz, 1H, H-5″); 7.83 (d, *J* = 2.6, 1H, H-4′); 7.67 (d, *J* = 9.1 Hz, 1H, H-7′); 7.67–7.61 (m, 1H, H-3″); 7.58–7.52 (m, 2H, H-6″ and H-7″); 7.05–7.01 (m, 1H, H-5‴); 6.98–6.93 (m, 2H, H-3‴ and H-4‴); 6.91 (dd, *J* = 9.1 and 2.6 Hz, 1H, H-6′); 6.88 (d, *J* = 7.8 Hz, 1H, H-6‴); 3.87 (s, 3H, 5′-OCH_3_); 3.81 (s, 3H, 2‴-OCH_3_); 3.72 (s, 2H, H-2); 3.15 (bs, 4H, H-3⁗); 2.83 (bs, 4H, H-2⁗). ^13^C-NMR (100.6 MHz, CDCl_3_) δ (ppm): 193.4, 157.6, 152.3, 141.1, 136.4, 134.3, 133.7, 132.8, 130.3, 129.5, 129.2, 129.1, 128.8, 128.0, 127.5, 124.2, 123.4, 123.1, 121.0, 118.7, 118.4, 115.5, 113.7, 111.3, 104.6, 66.8, 55.7, 55.4, 53.8 (2C), 50.5 (2C). HRMS calculated for C_32_H_32_N_3_O_5_S [M + H^+^]: 570.2057; Found: 570.2074.

*1-(5-methoxy-1-(naphthalen-1-ylsulfonyl)-1H-indol-3-yl)-2-morpholinoethan-1-one* (**3m**)

Morpholine (0.1 mL, 1.16 mmol), potassium carbonate (97 mg, 0.7 mmol) and 2-bromo-1-(5-methoxy-1-(naphthalen-1-ylsulfonyl)-1*H*-indol-3-yl)ethan-1-one (**2f**) (320 mg, 0.7 mmol) were reacted to give a solid, which was purified by column chromatography on silica gel using CH_2_Cl_2_/ethyl acetate 5:1 to give 319 mg of product (**3m**) as an orange solid. Yield: 98%; m.p.: 104–107 °C; IR (KBr) cm^−1^: 1657, 1366, 1173, 1216, 1116, 985. ^1^H-NMR (400.1 MHz, CDCl_3_) δ (ppm): 8.76 (s, 1H, H-2′); 8.58 (d, *J* = 8.6 Hz, 1H, H-2″); 8.23 (d, *J* = 7.5 Hz, 1H, H-8″); 8.03 (d, *J* = 8.2 Hz, 1H, H-4″); 7.83 (d, *J* = 8.1 Hz, 1H, H-5″); 7.72 (s, 1H, H-4′); 7.57 (d, *J* = 9.2 Hz, 1H, H-7′); 7.58–7.53 (m, 1H, H-3″); 7.53–7.46 (m, 2H, H-6″ and H-7″); 6.82 (dd, *J* = 9.1 and 1.9 Hz, 1H, H-6′); 3.72 (s, 3H, 5′-OCH_3_); 3.68 (t, *J* = 4.3 Hz, 4H, H-3⁗); 3.56 (s, 2H, H-2); 2.52 (t, *J* = 4.0 Hz, 4H, H-2⁗). ^13^C-NMR (100.6 MHz, CDCl_3_) δ (ppm): 193.2, 157.6, 136.4, 134.3, 133.4, 132.8, 130.3, 129.5, 129.1, 129.1, 128.7, 127.9, 127.5, 124.2, 123.3, 118.7, 115.5, 113.7, 104.5, 66.9, 66.8(2C), 55.7, 53.9(2C). HRMS calculated for C_25_H_25_N_2_O_5_S [M + H^+^]: 465.1479; Found: 465.1488.

*1-(5-methoxy-1-(naphthalen-1-ylsulfonyl)-1H-indol-3-yl)-2-(4-(pyridin-2-yl)piperazin-1-yl)ethan-1-one* (**3n**)

1-(2-Pyridyl)-piperazine (114 mg, 0.7 mmol), potassium carbonate (99 mg, 0.72 mmol) and 2-bromo-1-(5-methoxy-1-(naphthalen-1-ylsulfonyl)-1*H*-indol-3-yl)ethan-1-one (**2f**) (320 mg, 0.7 mmol) were reacted to give a solid, which was purified by column chromatography on silica gel using CH_2_Cl_2_/ethyl acetate 5:1 to give 360 mg of product (**3n**) as a brown solid. Yield: 95%; m.p.: 109–112 °C; IR (KBr) cm^−1^: 1654, 1381, 1174, 1215, 855. ^1^H-NMR (400.1 MHz, CDCl_3_) δ (ppm): 8.81 (s, 1H, H-2′); 8.56 (d, *J* = 8.6 Hz, 1H, H-2″); 8.23 (d, *J* = 7.5 Hz, 1H, H-8″); 8.14 (d, *J* = 4.6 Hz, 1H, H-3‴); 7.96 (d, *J* = 8.2 Hz, 1H, H-4″); 7.79–7.73 (m, 2H, H-4′ and H-5″); 7.61 (d, *J* = 9.1 Hz, 1H, H-7′); 7.55–7.38 (m, 4H, H-6′, H-3″, H-6″ and H-7″); 6.83 (d, *J* = 7.4 Hz, 1H, H-5‴); 6.60–6.53 (m, 2H, H-6‴ and H-4‴); 3.73 (s, 3H, 5′-OCH_3_); 3.59 (s, 2H, H-2); 3.47 (t, *J* = 4.7 Hz, 4H, H-3⁗ and H-5⁗); 2.64–2.57 (t, *J* = 4.6 Hz, 4H, H-2⁗ and H-6⁗). ^13^C-NMR (100.6 MHz, CDCl_3_) δ (ppm): 193.4, 159.4, 157.6, 148.0, 137.5, 136.4, 134.3, 133.6, 132.8, 130.3, 129.5, 129.2, 129.1, 128.8, 128.0, 127.5, 124.2, 123.3, 118.7, 115.5, 113.8, 113.5, 107.2, 104.6, 66.9, 55.7, 53.3(2), 45.2(2). HRMS calculated for C_30_H_29_N_4_O_4_S [M + H^+^]: 541.1904; Found: 541.1913.

*1-(5-methoxy-1-tosyl-1H-indol-3-yl)-2-(4-(2-methoxyphenyl)piperazin-1-yl)ethan-1-one* (**3o**)

1-(2-Methoxyphenyl)-piperazine (154 mg, 0.8 mmol), potassium carbonate (110 mg, 0.8 mmol) and 2-bromo-1-(5-methoxy-1-tosyl-1*H*-indol-3-yl)ethan-1-one (**2i**) (338 mg, 0.8 mmol) were reacted to give a solid, which was purified by column chromatography on silica gel using CH_2_Cl_2_/ethyl acetate 5:1 to give 264 mg of product (**3o**) as a yellow-orange solid. Yield: 62%; m.p.: 85–88 °C; IR (KBr) cm^−1^: 1663, 1377, 1173, 1241, 1029. ^1^H-NMR (400.1 MHz, CDCl_3_) δ (ppm): 8.57 (s, 1H, H-2′); 7.76–7.72 (m, 4H, H-2″, H-4′ and H-7′); 7.17 (d, *J* = 8.2 Hz, 2H, H-3″); 6.96–6.82 (m, 4H, H-3‴, H-4‴, H-5‴ and H-6′); 6.80 (d, *J* = 7.8 Hz, 1H, H-6‴); 3.79 (s, 3H, 5′-OCH_3_); 3.75 (s, 3H, 2‴-OCH_3_); 3.66 (s, 2H, H-2); 3.08 (bs, 4H, H-3⁗); 2.75 (bs, 4H, H-2⁗); 2.26 (s, 3H, 4″-CH_3_). ^13^C-NMR (100.6 MHz, CDCl_3_) δ (ppm): 193.4, 157.7, 152.3, 145.9, 141.1, 134.7, 133.3, 130.2 (2C), 129.1, 129.0, 127.1 (2C), 123.1, 121.0, 119.2, 118.3, 115.5, 113.9, 111.3, 104.5, 66.5, 55.7, 55.4, 53.7 (2C), 50.5(2C), 21.6. HRMS calculated for C_29_H_32_N_3_O_5_S [M + H^+^]: 534.2057; Found: 534.2076.

*1-(5-methoxy-1-tosyl-1H-indol-3-yl)-2-(4-(pyridin-2-yl)piperazin-1-yl)ethan-1-one* (**3p**)

1-(2-Pyridyl)-piperazine (89 mg, 0.55 mmol), potassium carbonate (76 mg, 0.55 mmol) and 2-bromo-1-(5-methoxy-1-tosyl-1*H*-indol-3-yl)ethan-1-one (**2i**) (230 mg, 0.54 mmol) were reacted to give a solid, which was purified by column chromatography on silica gel using CH_2_Cl_2_/ethyl acetate 5:1 to give 269 mg of product (**3p**) as a yellow solid. Yield: 99%; m.p.: 144–147 °C; IR (KBr) cm^−1^: 1654, 1385, 1174, 1216, 857. ^1^H-NMR (400.1 MHz, CDCl_3_) δ (ppm): 8.64 (s, 1H, H-2′); 8.21 (d, *J* = 3.8 Hz, 1H, H-3‴); 7.89–7.82 (m, 4H, H-4′, H-7′ and H-2″); 7.49 (t, *J* = 7.7 Hz, 1H, H-5‴); 7.24 (d, *J* = 8.0 Hz, 2H, H-3″); 6.98 (d, *J* = 9.0 Hz, 1H, H-6′); 6.72–6.56 (m, 2H, H-4‴ and H-6‴); 3.84 (s, 3H, 5′-OCH_3_); 3.69 (s, 2H, H-2); 3.61 (bs, 4H, H-3⁗ and H-5⁗); 2.71 (bs, 4H, H-2⁗ and H-6⁗); 2.33 (s, 3H, 4″-CH_3_). ^13^C-NMR (100.6 MHz, CDCl_3_) δ (ppm): 193.8, 159.8, 158.1, 148.4, 146.3, 137.9, 135.0, 133.7, 130.6(2C), 129.5, 129.4, 127.5(2C), 119.6, 115.9, 114.3, 113.9, 107.5, 104.9, 67.0, 56.1, 53.7 (2C), 45.6 (2C), 22.0. HRMS calculated for C_27_H_29_N_4_O_4_S [M + H^+^]: 505.1904; Found: 505.1920.

*1-(5-methoxy-1-tosyl-1H-indol-3-yl)-2-morpholinoethan-1-one* (**3q**)

Morpholine (0.1 mL, 1.16 mmol), potassium carbonate (101 mg, 0.73 mmol) and 2-bromo-1-(5-methoxy-1-tosyl-1*H*-indol-3-yl)ethan-1-one (**2i**) (311 mg, 0.74 mmol) were reacted to give a solid, which was purified by column chromatography on silica gel using CH_2_Cl_2_/ethyl acetate 5:1 to give 308 mg of product (**3q**) as a yellow solid. Yield: 97%; m.p.: 152–154 °C; IR (KBr) cm^−1^: 1663, 1377, 1174, 1216, 1086, 811. ^1^H-NMR (400.1 MHz, CDCl_3_) δ (ppm): 8.60 (s, 1H, H-2′), 7.97–7.73 (m, 4H, H-4′, H-7′, H-2″); 7.28 (d, *J* = 7.6 Hz, 2H, H-3″); 6.97 (d, *J* = 8.2 Hz, 1H, H-6′); 3.83 (s, 3H, 5′-OCH_3_); 3.78 (s, 4H, H-3⁗); 3.65 (s, 2H, H-2); 2.61 (bs, 4H, H-2⁗); 2.36 (s, 3H, 4″-CH_3_). ^13^C-NMR (100.6 MHz, CDCl_3_) δ (ppm): 193.5, 158.1, 146.3, 135.1, 133.5, 130.6(2C), 129.5, 129.4, 127.5(2C), 119.6, 115.9, 114.3, 104.9, 67.3 (2C), 67.1, 56.1, 54.2 (2C), 22,0. HRMS calculated for C_22_H_25_N_2_O_5_S [M + H^+^]: 429.1479; Found: 429.1494.

*1-(1-((5-bromothiophen-2-yl)sulfonyl)-5-methoxy-1H-indol-3-yl)-2-(4-(2-methoxyphenyl)piperazin-1-yl)ethan-1-one* (**3r**)

1-(2-Methoxyphenyl)-piperazine (116 mg, 0.6 mmol), potassium carbonate (84 mg, 0.61 mmol) and 2-bromo-1-(1-((5-bromothiophen-2-yl)sulfonyl)-5-methoxy-1*H*-indol-3-yl)ethan-1-one (**2h**) (300 mg, 0.61 mmol) were reacted to give a gel, which was purified by column chromatography on silica gel using CH_2_Cl_2_/ethyl acetate 5:1 to give 353 mg of product (**3r**) as a brown gel. Yield: 96%; m.p.: product in gel state; IR (KBr) cm^−1^: 1672, 1387, 1176, 1215, 858, 605. ^1^H-NMR (400.1 MHz, CDCl_3_) δ (ppm): 8.55 (s, 1H, H-2′); 7.80 (bs, 1H, H-4′); 7.74 (d, *J* = 9.1 Hz, 1H, H-7′); 7.44 (d, *J* = 4.0 Hz, 1H, H-4″); 6.97–6.83 (m, 5H, H-6′, H-3″, H-3‴, H-4‴, H-5‴); 6.80 (d, *J* = 7.9 Hz, 1H, H-6‴); 3.79 (s, 3H, 5′-OCH_3_); 3.78 (s, 3H, 2‴-OCH_3_); 3.63 (s, 2H, H-2); 3.09 (bs, 4H, H-3⁗ and H-5⁗); 2.74 (bs, 4H, H-2⁗ and H-6⁗). ^13^C-NMR (100.6 MHz, CDCl_3_) δ (ppm): 193.6, 158.0, 152.3, 141.1, 137.9, 134.2, 132.9, 130.9, 129.3, 128.7, 123.2, 123.1, 121.0, 120.0, 118.3, 115.8, 113.9, 111.3, 104.9, 66.8, 55.7, 55.4, 53.8 (2C), 50.6 (2C). HRMS calculated for C_26_H_27_BrN_3_O_5_S_2_ [M + H^+^]: 604.0570; Found: 604.0565.

*1-(1-((5-bromothiophen-2-yl)sulfonyl)-5-methoxy-1H-indol-3-yl)-2-morpholinoethan-1-one* (**3s**)

Morpholine (0.1 mL, 1.16 mmol), potassium carbonate (86 mg, 0.62 mmol) and 2-bromo-1-(1-((5-bromothiophen-2-yl)sulfonyl)-5-methoxy-1*H*-indol-3-yl)ethan-1-one (**2h**) (300 mg, 0.61 mmol) were reacted to give a solid, which was purified by column chromatography on silica gel using CH_2_Cl_2_/ethyl acetate 5:1 to give 277 mg of product (**3s**) as a yellow solid. Yield: 91%; m.p.: 130–133 °C; IR (KBr) cm^−1^: 1670, 1389, 1172, 1217, 1130, 854, 605. ^1^H-NMR (400.1 MHz, CDCl_3_) δ (ppm): 8.46 (s, 1H, H-2′); 7.77 (d, *J* = 2.7 Hz, 1H, H-4′); 7.73 (d, *J* = 9.1 Hz, 1H, H-7′); 7.45 (d, *J* = 4.1 Hz, 1H, H-4″); 6.96–6.92 (m, 2H, H-6′, H-3″); 3.78 (s, 3H, 5′-OCH_3_); 3.71 (t, *J* = 4.6 Hz, 4H, H-3⁗ and H-5⁗); 3.57 (s, 2H, H-2); 2.54 (t, *J* = 4.5 Hz, 4H, H-2⁗ and H-6⁗). ^13^C-NMR (100.6 MHz, CDCl_3_) δ (ppm): 193.1, 158.0, 137.9, 134.3, 132.7, 130.9, 129.2, 128.7, 123.3, 120.0, 115.8, 113.9, 104.9, 66.9 (2C), 66.8, 55.7, 53.8 (2C). HRMS calculated for C_19_H_20_BrN_2_O_5_S_2_ [M + H^+^]: 498.9992; Found: 499.0013.

*1-(5-methoxy-1-((4-methoxyphenyl)sulfonyl)-1H-indol-3-yl)-2-morpholinoethan-1-one* (**3t**)

Morpholine (0.1 mL, 1.16 mmol), potassium carbonate (97 mg, 0.7 mmol) and 2-bromo-1-(5-methoxy-1-((4-methoxyphenyl)sulfonyl)-1*H*-indol-3-yl)ethan-1-one (**2g**) (307 mg, 0.7 mmol) were reacted to give a gel, which was purified by column chromatography on silica gel using CH_2_Cl_2_/ethyl acetate 5:1 to give 305 mg of product (**3t**) as an orange gel. Yield: 98%; m.p.: product in gel state; IR (KBr) cm^−1^: 1656, 1382, 1164, 1215, 1114, 985. ^1^H-NMR (400.1 MHz, CDCl_3_) δ (ppm): 8.51 (s, 1H, H-2′) 7.79 (d, *J* = 8.9 Hz, 2H, H-2″ and H-6″); 7.75–7.71 (m, 2H, H-4′ and H-7′); 6.89 (dd, *J* = 9.1 and 2.5 Hz, 1H, H-6′); 6.80 (d, *J* = 8.9 Hz, 2H, H-3″ and H-5″); 3.75 (s, 3H, 5′-OCH_3_); 3.72 (s, 3H, 4″-OCH_3_); 3.70 (t, *J* = 4.5 Hz, 4H, H-3⁗ and H-5⁗); 3.57 (s, 2H, H-2); 2.53 (t, *J* = 4.4 Hz, 4H, H-2⁗ and H-6⁗). ^13^C-NMR (100.6 MHz, CDCl_3_) δ (ppm): 193.1, 164.4, 157.6, 133.0, 129.4 (2C), 129.0, 129.0, 128.8, 119.0, 115.5, 114.8 (2C), 113.9, 104.5, 66.9 (2C), 66.6, 55.8, 55.7, 53.8 (2C). HRMS calculated for C_22_H_25_N_2_O_6_S [M + H^+^]: 445.1428; Found: 445.1443.

*1-(5-methoxy-1-((4-methoxyphenyl)sulfonyl)-1H-indol-3-yl)-2-(4-(2-methoxyphenyl)piperazin-1-yl)ethan-1-one* (**3u**)

1-(2-Methoxyphenyl)-piperazine (135 mg, 0.7 mmol), potassium carbonate (98 mg, 0.71 mmol) and 2-bromo-1-(5-methoxy-1-((4-methoxyphenyl)sulfonyl)-1*H*-indol-3-yl)ethan-1-one (**2g**) (307 mg, 0.7 mmol) were reacted to give a solid, which was purified by column chromatography on silica gel using CH_2_Cl_2_/ethyl acetate 5:1 to give 362 mg of product (**3u**) as an orange-brown solid. Yield: 94%; m.p.: 73–76 °C; IR (KBr) cm^−1^: 1682, 1371, 1169, 1217, 985. ^1^H-NMR (400.1 MHz, CDCl_3_) δ (ppm): 8.59 (s, 1H, H-2′); 7.79 (d, *J* = 8.9 Hz, 2H, H-2″ and H-6″); 7.78–7.73 (m, 2H, H-7′, H-4′,); 6.96–6.76 (m, 7H, H-6′, H-3″, H-5″, H-3‴, H-4‴, H-5‴, H-6‴); 3.79 (s, 3H, 5′-OCH_3_); 3.76 (s, 3H, 2⁗-OCH_3_); 3.68 (s, 3H, 4″-OCH_3_); 3.63 (s, 2H, H-2); 3.07 (bs, 4H, H-3⁗ and H-5⁗); 2.72 (bs, 4H, H-2⁗ and H-6⁗). ^13^C-NMR (100.6 MHz, CDCl_3_) δ (ppm): 193.6, 164.3, 157.6, 152.3, 141.2, 133.3, 129.4 (2C), 129.1, 129.0, 128.9, 123.1, 121.0, 119.1, 118.2, 115.5, 114.8 (2C), 113.9, 111.3, 104.5, 66.7, 55.7, 55.6, 55.4, 53.8 (2C), 50.6 (2C). HRMS calculated for C_29_H_32_N_3_O_6_S [M + H^+^]: 550.2006; Found: 550.2023.

*1-(5-methoxy-1-((4-methoxyphenyl)sulfonyl)-1H-indol-3-yl)-2-(4-(pyridin-2-yl)piperazin-1-yl)ethan-1-one* (**3v**)

1-(2-Pyridyl)-piperazine (115 mg, 0.7 mmol), potassium carbonate (100 mg, 0.72 mmol) and 2-bromo-1-(5-methoxy-1-((4-methoxyphenyl)sulfonyl)-1*H*-indol-3-yl)ethan-1-one (**2g**) (305 mg, 0.7 mmol) were reacted to give a gel, which was purified by column chromatography on silica gel using CH_2_Cl_2_/ethyl acetate 5:1 to give 361 mg of product (**3v**) as a brown gel. Yield: 99%; m.p.: product in gel state; IR (KBr) cm^−1^: 1662, 1376, 1166, 1216, 980. ^1^H-NMR (400.1 MHz, CDCl_3_) δ (ppm): 8.56 (s, 1H, H-2′); 8.12 (d, *J* = 5.8 Hz, 1H, H-3‴); 7.80–7.72 (m, 4H, H-4′, H-7′, H-2″ and H-6″); 7.44–7.37 (m, 1H, H-5‴); 6.89 (dd, *J* = 9.1 and 2.6 Hz, 1H, H-6′); 6.81 (d, *J* = 8.9 Hz, 2H, H-3″ and H-5″); 6.60–6.53 (m, 2H, H-4‴, H-6‴); 3.76 (s, 3H, 5′-OCH_3_); 3.69 (s, 3H, 4″-OCH_3_); 3.61 (s, 2H, H-2); 3.52 (t, *J* = 4.9 Hz, 4H, H-3⁗ and H-5⁗); 2.63 (t, *J* = 4.9 Hz, 4H, H-2⁗ and H-6⁗). ^13^C-NMR (100.6 MHz, CDCl_3_) δ (ppm): 193.3, 164.3, 159.4, 157.6, 148.0, 137.6, 133.2, 129.4 (2C), 129.0, 129.0, 128.8, 119.1, 115.5, 114.8 (2C), 113.9, 113.5, 107.1, 104.5, 66.5, 55.7, 55.7, 53.3 (2C), 45.2 (2C). HRMS calculated for C_27_H_29_N_4_O_5_S [M + H^+^]: 521.1853; Found: 521.1866.

*General Procedure for 2-(4-(Aryl)piperazin-1-yl)-1-(1-arylsulfonyl-1H-indol-3-yl)ethanol Derivatives* (**4a**–**v**)

*1-(5-fluoro-1-((4-iodophenyl)sulfonyl)-1H-indol-3-yl)-2-(4-(2-methoxyphenyl)piperazin-1-yl) ethan-1-ol* (**4a**)

To a solution of (**3a**) (214 mg, 0.34 mmol) in ethanol (30 mL) was added sodium borohydride (19 mg, 0.51 mmol) in one portion and the mixture was vigorously stirred at room temperature until the starting material was disappeared upon checking TLC. Adding water (30 mL) stopped the reaction. The organic phase was extracted with CH_2_Cl_2_ (3 × 30 mL). The combined organic layers were dried with anhydrous sodium sulfate, and the removal of the solvent under vacuum afforded a residue, which was further purified by column chromatography on silica gel using CH_2_Cl_2_/ethyl acetate 5:1 to give 69 mg of (**4a**) as pale-yellow crystalline plates. Yield: 32%; m.p.: 94.5–96.4 °C; IR (KBr) cm^−1^: 3423, 1500, 1468, 1375, 1175, 1137, 751. ^1^H-NMR (400.1 MHz, CDCl_3_) δ (ppm): 7.82 (dd, *J* = 9.0 and 4.3 Hz, 1H, H-4′); 7,70 (d, *J* = 8.5 Hz, 2H, H-3″ and H-5″); 7.49 (s, 1H, H-2′); 7.47 (d, *J* = 8.8 Hz, 2H, H-2″ and H-6″); 7.24 (dd, *J* = 8.8 and 2.0 Hz, 1H, H-7′); 7.00–6.97 (m, 1H, H-6′); 6.95–6.92 (m, 1H, H-5‴); 6.87–6.84 (m, 2H, H-3‴ and H-4‴); 6.80 (d, *J* = 7.8 Hz, 1H, H-6‴); 4.92 (dd, *J* = 9.4 and 3.9 Hz, 1H, H-1); 3.80 (s, 3H, OCH_3_); 3.07 (bs, 4H, H-3⁗ and H-5⁗); 2.92–2.85 (m, 2H, H-2a⁗ and H-6a⁗); 2.69–2.64 (m, 4H, H-2, H-2b⁗ and H-6b⁗). ^13^C-NMR (100.6 MHz, CDCl_3_) δ (ppm): 159.9 (d, *J* = 241.0 Hz); 152.7, 141.4, 139.1 (2C), 137.9, 132.2, 130.6 (d, *J* = 10.0 Hz), 128.4 (2C), 124.9, 124.3 (d, *J* = 2.1 Hz), 123.6, 121.5, 118.7, 115.2 (d, *J* = 9.6 Hz), 113.5 (d, *J* = 25.8 Hz), 111.8, 106.9 (d, *J* = 24.3 Hz), 102.4, 64.2, 63.3, 55.8, 53.8 (2C), 51.1 (2C). HRMS calculated for C_27_H_28_FIN_3_O_4_S [M + H^+^]: 636.0824; Found: 636.0840.

*1-(5-fluoro-1-((4-iodophenyl)sulfonyl)-1H-indol-3-yl)-2-(4-(pyridin-2-yl)piperazin-1-yl)ethan-1-ol* (**4b**)

(**3b**) (556 mg, 0.92mmol) and sodium borohydride (52 mg, 1.38 mmol) were reacted to give a solid, which was purified by column chromatography on silica gel using dichloromethane/ethyl acetate 5:1 to obtain 197 mg of compound (**4b**) as an orange solid. Yield: 35%; m.p.: 135–137 °C; IR (KBr) cm^−1^: 3422, 1593, 1470, 1386, 1175, 1140, 735. ^1^H-NMR (400.1 MHz, CDCl_3_) δ (ppm): 8.20 (dd, *J* = 4.8 and 1.3 Hz, 1H, H-3‴); 7.90 (dd, *J* = 9.1 and 4.4 Hz, 1H, H-4′); 7.79 (d, *J* = 8.6 Hz, 2H, H-3″ and H-5″); 7.58–7.53 (m, 3H, H-2′, H-2″ and H-6″); 7.53–7.47 (m, 1H, H-5‴); 7.31 (dd, *J* = 8.8 and 2.5 Hz, 1H, H-7′); 7.05 (td, *J* = 9.0 and 2.5 Hz, 1H, H-6′); 6.69–6.61 (m, 2H, H-4‴ and H-6‴); 5.01 (dd, *J* = 10.2 and 3.3 Hz, 1H, H-1); 3.70–3.52 (m, 4H, H-3⁗ and H-5⁗); 2.92–2.84 (m, 2H, H-2a⁗ and H-6a⁗); 2.81–2.66 (m, 2H, H-2); 2.66–2.58 (m, 2H, H-2b⁗ and H-6b⁗). ^13^C-NMR (100.6 MHz, CDCl_3_) δ (ppm): 159.7 (d, *J* = 240.0 Hz), 159.5, 148.1, 138.8 (2C), 137.7, 137.6, 131.9, 130.2 (d, *J* = 9.9 Hz), 128.1 (2C), 124.6, 123.9 (d, *J* = 4.1 Hz). 114.9 (d, *J* = 9.4 Hz), 113.8, 113.3 (d, *J* = 25.6 Hz), 107.3, 106.5 (d, *J* = 24.4 Hz), 102.1, 64.0, 63.1, 53.1 (2C), 45.4 (2C). ^19^F-NMR (376.5 MHz, CDCl_3_) δ (ppm): −118.75. HRMS calculated for C_25_H_25_FIN_4_O_3_S [M + H^+^]: 607.0671; Found: 607.0682.

*1-(5-fluoro-1-((4-iodophenyl)sulfonyl)-1H-indol-3-yl)-2-morpholinoethan-1-ol* (**4c**)

(**3c**) (175 mg, 0.33 mmol) and sodium borohydride (19 mg, 0.51 mmol) were reacted to give a gel, which was purified by column chromatography on silica gel using dichloromethane/ethyl acetate 5:1 to obtain 68 mg of compound (**4c**) as a pale-yellow gel. Yield: 39%; m.p.: product in gel state; IR (KBr) cm^−1^: 3423, 1567, 1469, 1375, 1176, 1115, 736. ^1^H-NMR (400.1 MHz, CDCl_3_) δ (ppm): 7.89 (dd, *J* = 9.1 and 4.4 Hz, 1H, H-4′); 7.79 (d. *J* = 8.4 Hz, 2H, H-3″ and H-5″); 7.58–7.51 (m, 3H, H-2′, H-2″ and H-6″); 7.28 (dd, *J* = 8.8 and 2.5 Hz, 1H, H-7′); 7.05 (td, *J* = 9.0 and 2.5 Hz, 1H, H-6′); 4.96 (dd, *J* = 8.3 and 5.5 Hz, 1H, H-1); 3.82–3.71 (m, 4H, H-3⁗ and H-5⁗); 2.80–2.70 (m, 2H, H-2a⁗ and H-6a⁗); 2.70–2.62 (m, 2H, H-2); 2.55–2.46 (m, 2H, H-2b⁗ and H-6b⁗). ^13^C-NMR (100.6 MHz, CDCl_3_) δ (ppm): 159.7 (d, *J* = 240.9 Hz), 138.8 (2C), 137.6, 131.9, 130.2 (d, *J* = 9.9 Hz), 128.1 (2C), 124.6, 123.8 (d, *J* = 4.1 Hz), 114.9 (d, *J* = 9.4 Hz), 113.3 (d, *J* = 25.7 Hz), 106.5 (d, *J* = 24.3 Hz), 102.1, 67.1 (2C), 64.4, 62.9, 53.7 (2C). ^19^F-NMR (376.5 MHz, CDCl_3_) δ (ppm): −118.77. HRMS calculated for C_20_H_21_FIN_2_O_4_S [M + H^+^]: 531.0245; Found: 531.0253.

*1-(5-fluoro-1-(naphthalen-1-ylsulfonyl)-1H-indol-3-yl)-2-(4-(2-methoxyphenyl)piperazin-1-yl)ethan-1-ol* (**4d**)

(**3d**) (601 mg, 1.1 mmol) and sodium borohydride (62 mg, 1.65 mmol) were reacted to give a solid, which was purified by column chromatography on silica gel using dichloromethane/ethyl acetate 5:1 to obtain 499 mg of compound (**4d**) as pale orange crystalline plates. Yield: 81%; m.p.: 181.1–183.2 °C; IR (KBr) cm^−1^: 3449, 1593, 1500, 1370, 1174, 1139. ^1^H-NMR (400.1 MHz, CDCl_3_) δ (ppm): 8.71 (d, *J* = 8.7 Hz, 1H, H-2″); 8.09–8.02 (m, 2H, H-4″ and H-8″); 7.89 (d, *J* = 8.1 Hz, 1H, H-5″); 7.81–7.75 (m, 2H, H-2′ and H-4′); 7.68–7.62 (m, 1H, H-7″); 7.57 (t, *J* = 7.5 Hz, 1H, H-6″); 7.50 (t, *J* = 7.9 Hz, 1H, H-3″); 7.34 (dd, *J* = 8.9 and 2.5 Hz, 1H, H-7′); 7.06–6.91 (m, 4H, H-6′, H-3‴, H-4‴ and H-5‴); 6.89 (d, *J* = 7.7 Hz, 1H, H-6‴); 5.01 (dd, *J* = 10.1 and 3.5 Hz, 1H, H-1); 3.88 (s, 3H, OCH_3_); 3.14 (bs, 4H, H-3⁗ and H-5⁗); 3.02–2.91 (m, 2H, H-2a⁗ and H-6a⁗); 2.83–2.65 (m, 4H, H-2, H-2b⁗ and H-6b⁗).^13^C-NMR (100.6 MHz, CDCl_3_) δ (ppm): 159.4 (d, *J* = 240.5 Hz), 152.4, 141.2, 135.7, 134.4, 134.0, 132.0, 129.8 (d, *J* = 10.1 Hz), 129.3 (d, *J* = 7.5 Hz), 129.0, 128.2, 127.4, 125.2, 124.2, 124.1, 123.3, 122.5 (d, *J* = 4.2 Hz), 121.2, 118.4, 114.7 (d, *J* = 9.4 Hz), 113.1, 112.8, 111.4, 106.5 (d, *J* = 24.3 Hz), 63.9, 63.1, 55.5, 53.5 (2C), 50.8 (2C). ^19^F-NMR (376.5 MHz, CDCl_3_) δ (ppm): −119.47. HRMS calculated for C_31_H_31_FN_3_O_4_S [M + H^+^]: 560.2014; Found: 560.2029.

*1-(5-fluoro-1-(naphthalen-1-ylsulfonyl)-1H-indol-3-yl)-2-(4-(pyridin-2-yl)piperazin-1-yl)ethan-1-ol* (**4e**)

(**3e**) (493 mg, 0.93 mmol) and sodium borohydride (53 mg, 1.40 mmol) were reacted to give a solid, which was purified by column chromatography on silica gel using dichloromethane/ethyl acetate 5:1 to obtain 99 mg of compound (**4e**) as an orange gel. Yield: 57%; m.p.: 112–114 °C; IR (KBr) cm^−1^: 3421, 1565, 1481, 1367, 1171, 1140. ^1^H-NMR (400.1 MHz, CDCl_3_) δ (ppm): 8,70 (d, *J* = 8,7 Hz, 1H, H-2″); 8.21 (d, *J* = 3.9 Hz, 1H, H-3‴); 8.11–8.00 (m, 2H, H-4″ and H-8″); 7.87 (d, *J* = 8,2 Hz, 1H, H-5″); 7.81–7.74 (m, 2H, H-2′ and H-4′); 7.64 (t, *J* = 7.8 Hz, H1, H-7″); 7.55 (d, *J* = 7.5 Hz, 1H, H-6″); 7,49 (t, *J* = 7.9 Hz, 2H, H-3″ and H-5‴); 7.32 (dd, J = 8.9 and 2.6 Hz, 1H, H-7′); 6.98 (td, *J* = 9.0 and 2.6 Hz, 1H, H-6′); 6.70–6.60 (m, 2H, H-4‴ and H-6‴); 5.02 (dd, *J* = 10.4 and 3.4 Hz, 1H, H-1); 3.67–3.49 (bs, 4H, H-3⁗ and H-5⁗); 2.89–2.80 (bs, 2H, H-2a⁗ and H-6a⁗); 2.80–2,63 (m, 2H, H-2); 2.63–2.54 (m, 2H, H-2b⁗ and H-6b⁗). ^13^C-NMR (100.6 MHz, CDCl_3_) δ (ppm): 159.5, 159.4 (d, *J* = 240.5 Hz), 148.1, 137.7, 135.8, 134.4, 133.9, 131.9, 129.7 (d, *J* = 10.0 Hz), 129.3 (d, *J* = 4.1 Hz), 129.0, 128.2, 127.4, 125.2, 124.2, 124.0, 122.4 (t, *J* = 4.2 Hz), 114.7 (d, *J* = 9.4 Hz), 113.7, 113.1, 112.8, 107.3, 106.4 (d, *J* = 24.3 Hz), 64.0, 63.2, 53.1 (2C), 45.4 (2C). ^19^F-NMR (376.5 MHz, CDCl_3_) δ (ppm): −119.40. HRMS calculated for C_29_H_28_FN_4_O_3_S [M + H^+^]: 531.1861; Found: 531.1868.

*1-(5-fluoro-1-(naphthalen-1-ylsulfonyl)-1H-indol-3-yl)-2-morpholinoethan-1-ol* (**4f**)

(**3f**) (360 mg, 0.80 mmol) and sodium borohydride (45 mg, 1.20 mmol) were reacted to give a solid, which was purified by column chromatography on silica gel using dichloromethane/ethyl acetate 5:1 to obtain 191 mg of compound (**4f**) as pale-yellow crystalline plates. Yield: 53%; m.p.: 164.1–165.8 °C; IR (KBr) cm^−1^: 3449, 1592, 1470, 1373, 1173, 1139. ^1^H-NMR (400.1 MHz, CDCl_3_) δ (ppm): 8.69 (d, *J* = 8.6 Hz, 1H, H-2″); 8.12–7.99 (m, 2H, H-4″ and H-8″); 7.88 (d, *J* = 8.1 Hz, 1H, H-5″); 7.81–7.72 (m, 2H, H-2′ and H-4′); 7.64 (t, *J* = 7.7 Hz,1H, H-7″); 7.56 (t, *J* = 7.5 Hz, 1H, H-6″); 7.49 (t, *J* = 7.9 Hz, 1H, H-3″); 7.30 (dd, *J* = 8.9 and 1.6 Hz, 1H, H-7′); 6.98 (td, J = 9.0 and 1.7 Hz, 1H, H-6′); 5.00 (dd, *J* = 9.9 and 3.5 Hz, 1H, H-1); 3.85–3.65 (m, 4H, H-3⁗ and H-5⁗); 3.37 (s, 1H, OH); 2.80–2.72 (bs, 2H, H-2a⁗ and H-6a⁗); 2.72–2.61 (m, 2H, H-2); 2.55–2.47 (bs, 2H, H-2b⁗ and H-6b⁗). ^13^C-NMR (100.6 MHz, CDCl_3_) δ (ppm): 159.4 (d, *J* = 240,5 Hz), 135.8, 134.4, 134.0, 132.0, 129.7 (d, *J* = 9.9 Hz), 129.3 (d, *J* = 3.5 Hz), 129.0, 128.2, 127.5, 125.2, 124.2, 124.1, 122.2 (d, *J* = 4.2 Hz), 114.8 (d, *J* = 9.4 Hz), 113.1, 112.9, 106.4 (d, *J* = 24.4 Hz), 67.0 (2C), 64.4, 63.0, 53.7 (2C). ^19^F-NMR (376.5 MHz, CDCl_3_) δ (ppm): −119.42. HRMS calculated for C_24_H_24_FN_2_O_4_S [M + H^+^]: 455.1435; Found: 455.1440.

*1-(5-fluoro-1-(naphthalen-1-ylsulfonyl)-1H-indol-3-yl)-2-(4-(pyrimidin-2-yl)piperazin-1-yl)ethan-1-ol* (**4g**)

(**3g**) (264 mg, 0.50 mmol) and sodium borohydride (28 mg, 0.75 mmol) were reacted to give a solid, which was purified by column chromatography on silica gel using dichloromethane/ethyl acetate 5:1 to obtain 220 mg of compound (**4g**) as a white-orange solid. Yield: 83%; m.p.: 100–103 °C; IR (KBr) cm^−1^: 3423, 1586, 1470, 1360, 1172, 1139. ^1^H-NMR (400.1 MHz, CDCl_3_) δ (ppm): 8.69 (d, *J* = 8.6 Hz, 1H, H-2″); 8.31 (d, *J* = 4.7 Hz, 2H, H-3‴ and H-5‴); 8.06 (d, *J* = 7,4 Hz, 1H, H-4″); 8.03 (d, *J* = 8.2 Hz, 1H, H-8″); 7.86 (d, *J* = 8.1 Hz, 1H, H-5″); 7.80–7.73 (m, 2H, H-2′ and H-4′); 7.66–7.60 (m, 1H, H-7″); 7.54 (t, *J* = 7.5 Hz, 1H, H-6″); 7.48 (t, *J* = 7.8 Hz, 1H, H-3″); 7.32 (dd, *J* = 8.9 and 2.4 Hz, 1H, H-7′); 6.97 (td, *J* = 9.0 and 2.4 Hz, 1H, H-6′); 6.49 (t, *J* = 4.7 Hz, 1H, H-4‴); 5.02 (dd, 10.3 and 3.0 Hz, 1H, H-1); 3.95–3.79 (m, 4H, H-3⁗ and H-5⁗); 2.84–2.71 (m, 3H, H-2a, H-2a⁗ and H-6a⁗); 2.64 (dd, *J* = 12.6 and 3.4 Hz, 1H, H-2b); 2.58–2.49 (m, 2H, H-2b⁗ and H-6b⁗). ^13^C-NMR (100.6 MHz, CDCl_3_) δ (ppm): 161.7, 159.4 (d, *J* = 240.5 Hz), 157.8 (2C), 135.7, 134.3, 133.9, 131.9, 129.7 (d, *J* = 10.0 Hz), 129.3 (d, *J* = 2.4 Hz), 128.9, 128.2, 127.4, 125.1, 124.2, 124.0, 122.3 (d, *J* = 4.2 Hz); 114.7 (d, *J* = 9.4 Hz), 113.0, 112.8, 110.2, 106.4 (d, *J* = 24.3 Hz); 64.0, 63.2, 53.1 (2C), 43.8 (2C). ^19^F-NMR (376.5 MHz, CDCl_3_) δ (ppm): −119.38. HRMS calculated for C_28_H_27_FN_5_O_3_S [M + H^+^]: 532.1813; Found: 532.1830.

*1-(5-fluoro-1-((4-methoxyphenyl)sulfonyl)-1H-indol-3-yl)-2-(4-(2-methoxyphenyl)piperazin-1-yl)ethan-1-ol* (**4h**)

(**3h**) (411 mg, 0.77 mmol) and sodium borohydride (44 mg, 1.16 mmol) were reacted to give a gel, which was purified by column chromatography on silica gel using dichloromethane/ethyl acetate 5:1 to obtain 308 mg of compound (**4h**) as a pale-yellow gel. Yield: 74%; m.p.: product in gel state; IR (KBr) cm^−1^: 3424, 1594, 1499, 1372, 1188, 1165, 803. ^1^H-NMR (400.1 MHz, CDCl_3_) δ (ppm): 7,93 (dd, *J* = 9.1 and 4.4 Hz, 1H, H-4′); 7.80 (d, *J* = 9.0 Hz, 2H, H-2″ and H-6″); 7.60 (s, 1H, H-2′); 7.33 (dd, *J* = 8.9 and 2.5 Hz, 1H, H-7′); 7.08–6.98 (m, 2H, H-6′ and H-5‴); 6.97–6.90 (m, 2H, H-3‴ and H-4‴); 6.90–6.84 (m, 3H, H-3″, H-5″ and H-6‴); 4.99 (dd, *J* = 10.1 and 3.6 Hz, 1H, H-1); 3.87 (s, 3H, 2‴-OCH_3_); 3.77 (s, 3H, 4″-OCH_3_); 3.14 (bs, 4H, H-3⁗ and H-5⁗); 3.01–2.90 (bs, 2H, H-2a⁗ and H-6a⁗); 2.82–2.63 (m, 4H, H-2, H-2b⁗ and H-6b⁗). ^13^C-NMR (100.6 MHz, CDCl_3_) δ (ppm): 164.0, 159.4 (d, *J* = 240.1 Hz), 152.3, 141.1, 131.9, 130.1 (d, *J* = 9.9 Hz), 129.5, 129.2 (2C), 124.7, 123.2, 123.1 (d, *J* = 4.1 Hz), 121.1, 118.3, 114.8 (d, *J* = 9.4 Hz), 114.6 (2C), 112.8 (d, *J* = 25.6 Hz), 111.4, 106.3 (d, *J* = 24.3 Hz), 63.8, 63.0, 55.7, 55.5, 53.4 (2C), 50.8 (2C). ^19^F-NMR (376.5 MHz, CDCl_3_) δ (ppm): −119.47. HRMS calculated for C_28_H_31_FN_3_O_5_S [M + H^+^]: 540.1963; Found: 540.2001.

*1-(1-((5-bromothiophen-2-yl)sulfonyl)-5-fluoro-1H-indol-3-yl)-2-(4-(2-methoxyphenyl)piperazin-1-yl)ethan-1-ol* (**4i**)

(**3i**) (297 mg, 0.50 mmol) and sodium borohydride (28 mg, 0.75 mmol) were reacted to give a gel, which was purified by column chromatography on silica gel using dichloromethane/ethyl acetate 5:1 to obtain 215 mg of compound (**4i**) as a yellow gel. Yield: 72%; m.p.: product in gel state; IR (KBr) cm^−1^: 3422, 1594, 1500, 1381, 1173, 1138. ^1^H-NMR (400.1 MHz, CDCl_3_) δ (ppm): 7.90 (dd, *J* = 9.0 and 4.3 Hz, 1H, H-4′); 7.53 (s, 1H, H-2′); 7.41–7.32 (m, 2H, H-2″ and H-7′); 7.08 (td, *J* = 8.9 and 1.9 Hz, 1H, H-6′); 7.03–6.96 (m, 1H, H-5‴); 6.96–6.96 (m, 3H, H-3″, H-3‴ and H-4‴); 6.86 (d, *J* = 7.9 Hz, 1H, H-6‴); 5.00 (dd, *J* = 9.6 and 3.7 Hz, 1H, H-1); 3.85 (s, 3H, 2‴-OCH_3_); 3.12 (bs, 4H, H-3⁗ and H-5⁗); 2.99–2.87 (bs, 2H, H-2a⁗ and H-6a⁗); 2.81–2.63 (m, 4H, H-2, H-2b⁗ and H-6b⁗). ^13^C-NMR (100.6 MHz, CDCl_3_) δ (ppm): 159.7 (d, *J* = 241.2 Hz), 152.3, 141.1, 138.3, 133.5, 131.7, 130.5, 130.4 (d, *J* = 10.0 Hz), 124.8 (d, *J* = 3.9 Hz), 124.3, 123.2, 122.1, 121.1, 118.3, 115.0 (d, *J* = 9.4 Hz), 113.2 (d, *J* = 25.6 Hz), 111.4, 106.7 (d, *J* = 24.4 Hz), 63.8, 63.0, 55.4, 53.4 (2C), 50.7 (2C). HRMS calculated for C_25_H_26_BrFN_3_O_4_S_2_ [M + H^+^]: 594.0527; Found: 594.0547.

*1-(1-((4-iodophenyl)sulfonyl)-5-methoxy-1H-indol-3-yl)-2-(4-(2-methoxyphenyl)piperazin-1-yl)ethan-1-ol* (**4j**)

(**3j**) (416 mg, 0.60 mmol) and sodium borohydride (30 mg, 0.80 mmol) were reacted to give a solid, which was purified by column chromatography on silica gel using dichloromethane/ethyl acetate 5:1 to obtain 142 mg of compound (**4j**) as pale-brown crystalline plates. Yield: 31%; m.p.: 101–104 °C; IR (KBr) cm^−1^: 3406, 1372, 1221, 1174, 1029, 609. ^1^H-NMR (400.1 MHz, CDCl_3_) δ (ppm): 7.83 (d, *J* = 9.1 Hz, 1H, H-7′); 7.73 (d, *J* = 8.5 Hz, 2H, H-2″ and H-6″); 7.55–7.50 (m, 3H, H-2′, H-3″ and H-5″); 7.11 (d, *J* = 2.3 Hz, 1H, H-4′); 7.05–6.99 (m, 1H, H-5‴); 6.95–6.90 (m, 3H, H6′, H3‴, H4‴); 6.87 (d, *J* = 8.0 Hz, 1H, H6‴); 5.12 (dd, *J* = 9.3 and 3.2 Hz, 1H, H-1); 3.86 (s, 3H, 2‴-OCH_3_); 3.82 (s, 3H, 5′-OCH_3_); 3.18 (bs, 4H, H-3⁗ and H-5⁗); 3.05 (bs, 2H, H-2a⁗ and H-6a⁗); 2.91–2.80 (m, 4H, H-2, H-2b⁗ and H-6b⁗). ^13^C-NMR (100.6 MHz, CDCl_3_) δ (ppm): 156.6, 152.3, 140.8, 138.6 (2C), 137.7, 130.2, 130.1, 128.1 (2C), 124.1, 123.8, 123.4, 121.2, 118.4, 114.6, 114.0, 111.4, 103.4, 101.8, 63.6, 63.0, 55.9, 55.5, 53.6 (2C), 50.3 (2C). HRMS calculated for C_28_H_31_IN_3_O_5_S [M + H^+^]: 648.1024; Found: 648.1040.

*1-(1-((4-iodophenyl)sulfonyl)-5-methoxy-1H-indol-3-yl)-2-morpholinoethan-1-ol* (**4k**)

(**3k**) (374 mg, 0.70 mmol) and sodium borohydride (31 mg, 0.80 mmol) were reacted to give a solid, which was purified by column chromatography on silica gel using dichloromethane/ethyl acetate 5:1 to obtain 173 mg of compound (**4k**) as orange crystalline plates. Yield: 44%; m.p.: 120–124 °C; IR (KBr) cm^−1^: 3423, 1374, 1225, 1175, 1115, 974, 614. ^1^H-NMR (400.1 MHz, DMSO-d_6_) δ (ppm): 7.96 (d, *J* = 8.1 Hz, 2H, H-2′’ and H-6′’); 7.81 (d, *J* = 9.0 Hz, 1H, H-7′); 7.65 (d, *J* = 8.1 Hz, 2H, H-3′’ and H-5′’); 7.59 (s, 1H, H-2′); 7.19 (s, 1H, H-4′); 6.96 (d, *J* = 9.0 Hz, 1H, H-6′); 5.32 (s, 1H, OH); 4.99 (s, 1H, H-1); 3.77 (s, 3H, 5′-OCH_3_); 3.56 (bs, 4H, H-3⁗ and H-5⁗); 2.71 (bs, 2H, H-2a⁗ and H-6a⁗); 2.61–2.44 (bs, 4H, H-2, H-2b⁗ and H-6b⁗). ^13^C-NMR (100.6 MHz, DMSO-d_6_) δ (ppm): 156.4, 139.1 (2C), 136.9, 130.9, 129.7, 128.5 (2C), 127.0, 124.3, 114.6, 114.0, 104.2, 104.0, 66.4 (2C), 64.4, 63.7, 55.9, 54.0 (2C). HRMS calculated for C_21_H_24_IN_2_O_5_S [M+H^+^]: 543.0445; Found: 543.0453.

*1-(5-methoxy-1-(naphthalen-1-ylsulfonyl)-1H-indol-3-yl)-2-(4-(2-methoxyphenyl)piperazin-1-yl)ethan-1-ol* (**4l**)

(**3l**) (370 mg, 0.60 mmol) and sodium borohydride (30 mg, 0.80 mmol) were reacted to give a solid, which was purified by column chromatography on silica gel using dichloromethane/ethyl acetate 5:1 to obtain 175 mg of compound (**4l**) as brown crystalline plates. Yield: 47%; m.p.: 105–107 °C; IR (KBr) cm^−1^: 3423, 1362, 1221, 1172, 1026. ^1^H-NMR (400.1 MHz, CDCl_3_) δ (ppm): 8.73 (d, *J* = 8.7 Hz, 1H, H-2″); 8.01 (t, *J* = 7.2 Hz, 2H, H-4″ and H-8″); 7.87 (d, *J* = 8.1 Hz, 1H, H-5″); 7.73 (d, *J* = 7.9 Hz, 1H, H-7′); 7.72 (s, 1H, H-2′); 7.64 (t, *J* = 7.7 Hz, 1H, H-7″); 7.55 (t, *J* = 7.5 Hz, 1H, H-6″); 7.47 (t, *J* = 7.9 Hz, 1H, H-3″); 7.10 (d, *J* = 2.3 Hz, 1H, H-4′); 7.06–6.98 (m, 1H, H-5‴); 6.99–6.91 (m, 2H, H-3‴ and H-6′); 6.92–6.84 (m, 2H, H-4‴ and H-6‴); 5.05 (dd, *J* = 10.1 and 3.3 Hz, 1H, H-1); 3.88 (s, 3H, 2‴-OCH_3_); 3.80 (s, 3H, 5′-OCH_3_); 3.14 (bs, 4H, H-3⁗ and H-5⁗); 2.97 (bs, 2H, H-2a⁗ and H-6a⁗); 2.87–2.65 (m, 4H, H-2, H-2b⁗ and H-6b⁗). ^13^C-NMR (100.6 MHz, CDCl_3_) δ (ppm): 156.3, 152.3, 141.1, 135.3, 134.3, 134.2, 130.3, 129.7, 129.1, 128.9, 128.7, 128.2, 127.2, 124.2, 124.1, 124.1, 123.2, 122.4, 121.0, 118.3, 114.4, 113.6, 111.3, 103.2, 63.8, 63.0, 55.8, 55.4, 53.4 (2C), 50.7 (2C). HRMS calculated for C_32_H_34_N_3_O_5_S [M+H^+^]: 572.2214; Found: 572.2227.

*1-(5-methoxy-1-(naphthalen-1-ylsulfonyl)-1H-indol-3-yl)-2-morpholinoethan-1-ol* (**4m**)

(**3m**) (361 mg, 0.80 mmol) and sodium borohydride (35 mg, 0.90 mmol) were reacted to give a solid, which was purified by column chromatography on silica gel using dichloromethane/ethyl acetate 5:1 to obtain 234 mg of compound (**4m**) as white crystalline plates. Yield: 50%; m.p.: 190–192 °C; IR (KBr) cm^−1^: 3423, 1364, 1221, 1175, 1116, 1025. ^1^H-NMR (400.1 MHz, DMSO-d_6_) δ (ppm): 8.65 (d, *J* = 8.6 Hz, 1H, H-2″), 8.31–8.25 (m, 2H, H-4″ and H-8″); 8.08 (d, *J* = 8.1 Hz, 1H, H-5″); 7.89 (s, 1H, H-2′); 7.77–7.62 (m, 4H, H-7′, H-3″, H-6″ and H-7″); 7.19 (s, 1H, H-4′); 6.89 (d, *J* = 9.0 Hz, 1H, H-6′); 5.23 (bs, 1H, OH); 4.98 (bs, 1H, H-1); 3.74 (s, 3H, 5′-OCH_3_); 3.50 (s, 4H, H-3⁗ and H-5⁗); 2.71–255 (m; 2H, H-2a⁗ and H-6a⁗); 2.50–2.38 (m, 4H, H-2, H-2b⁗ and H-6b⁗). ^13^C-NMR (100.6 MHz, DMSO-d_6_) δ (ppm): 156.1, 136.4, 134.3, 133.1, 130.5, 130.3, 129.9, 129.5, 129.4, 127.9, 127.6, 125.5, 125.1, 124.7, 123.8, 114.2, 113.8, 104.2, 66.7 (2C), 64.9, 64.0, 55.9, 54.2 (2C). HRMS calculated for C_25_H_27_N_2_O_5_S [M + H^+^]: 467.1635; Found: 467.1651.

*1-(5-methoxy-1-(naphthalen-1-ylsulfonyl)-1H-indol-3-yl)-2-(4-(pyridin-2-yl)piperazin-1-yl)ethan-1-ol* (**4n**)

(**3n**) (336 mg, 0.60 mmol) and sodium borohydride (28 mg, 0.70 mmol) were reacted to give a gel, which was purified by column chromatography on silica gel using dichloromethane/ethyl acetate 5:1 to obtain 286 mg of compound (**4n**) as a yellow gel. Yield: 63%; m.p.: product in gel state; IR (KBr) cm^−1^: 3422, 1362, 1218, 1171, 981. ^1^H-NMR (400.1 MHz, CDCl_3_) δ (ppm): 8.72 (d, *J* = 8.7 Hz; 1H, H-2″); 8.20 (d, *J* = 3.9 Hz, 1H, H-3‴); 8.00 (t, *J* = 7.0 Hz, 2H, H-4″ and H-8″); 7.84 (d, *J* = 8.1 Hz, 1H, H-5″); 7.73 (s, 1H, H-2′), 7.72 (d, *J* = 7.5 Hz, 2H, H-7′); 7.63 (t, *J* = 7.7 Hz, 1H, H-7″); 7.56–7,41 (m, 3H, H-3″, H-6″ and H-5‴); 7.11 (s, 1H, H-4′); 6.86 (d, *J* = 9.0 Hz, 1H, H-6′); 6.64 (d, *J* = 7.6 Hz, 2H, H-4‴, H-6‴); 5.09 (d, *J* = 9.8 Hz, 1H, H-1); 4.32 (bs, 1H, OH); 3.79 (s, 3H, 5′-OCH_3_); 3.69–3.36 (m, 4H, H-3⁗ and H-5⁗); 2.92–2.78 (m, 3H, H-2a and H-2a⁗ and H-6a⁗); 2.74–2.59 (m, 3H, H-2b and H-2b⁗ and H-6b⁗). ^13^C-NMR (100.6 MHz, CDCl_3_) δ (ppm): 159.3, 156.2, 148.0, 137.6, 135.4, 134.2, 134.1, 130.2, 129.6, 129.1, 129.0, 128.7, 128.1, 127.2, 124.2, 124.1, 124.1, 122.3, 114.4, 113.7, 113.7, 107.3, 103.2, 63.8, 63.1, 55.8, 53.0 (2C), 45.1 (2C). HRMS calculated for C_30_H_31_N_4_O_4_S [M + H^+^]: 543.2061; Found: 543.2065.

*1-(5-methoxy-1-tosyl-1H-indol-3-yl)-2-(4-(2-methoxyphenyl)piperazin-1-yl)ethan-1-ol* (**4o**)

(**3o**) (250 mg, 0.50 mmol) and sodium borohydride (35 mg, 0.90 mmol) were reacted to give a solid, which was purified by column chromatography on silica gel using dichloromethane/ethyl acetate 5:1 to obtain 57 mg of compound (**4o**) as a pale-brown crystalline plate. Yield: 23%; 85–88 °C; IR (KBr) cm^−1^: 3423, 1369, 1222, 1171, 1029. ^1^H-NMR (400.1 MHz, CDCl_3_) δ (ppm): 7.87 (d, *J* = 9.0 Hz, 1H, H-7′); 7.73 (d, *J* = 8.2 Hz, 2H, H-2″ and H-6″); 7.53 (s, 1H, H-2′); 7.19 (d, *J* = 8.2 Hz, 2H, H-3″ and H-5″); 7.08 (d, *J* = 2.4 Hz, 1H, H-4′); 7.05–6.98 (m, 1H, H-5‴); 6.97–6.90 (m, 3H, H-6′, H-3‴, H-4‴); 6.87 (d, *J* = 7.9 Hz, 1H, H-6‴); 5.05 (dd, *J* = 10.0 and 3.4 Hz, 1H, H-1); 3.87 (s, 3H, 2‴-OCH_3_); 3.82 (s, 3H, 5′-OCH_3_); 3.16 (bs, 4H, H-3⁗ and H-5⁗); 3.00 (bs, 2H, H-2a⁗ and H-6a⁗); 2.90–2.67 (m, 4H, H-2, H-2b⁗ and H-6b⁗); 2.32 (s, 3H, 4″-CH_3_). ^13^C-NMR (100.6 MHz, CDCl_3_) δ (ppm): 156.4, 152.4, 145.0, 141.0, 135.3, 130.3, 130.1, 130.0 (2C), 126.9 (2C), 123.9, 123.3, 123.2, 121.2, 118.4, 114.7, 113.7, 111.4, 103.2, 63.8, 63.1, 55.9, 55.5, 53.5 (2C), 50.6 (2C), 21.6. HRMS calculated for C_29_H_34_N_3_O_5_S [M + H^+^]: 536.2214; Found: 536.2228.

*1-(5-methoxy-1-tosyl-1H-indol-3-yl)-2-(4-(pyridin-2-yl)piperazin-1-yl)ethan-1-ol* (**4p**)

(**3p**) (269 mg, 0.50 mmol) and sodium borohydride (24 mg, 0.60 mmol) were reacted to give a gel, which was purified by column chromatography on silica gel using dichloromethane/ethyl acetate 5:1 to obtain 286 mg of compound (**4p**) as pale-brown crystalline plates. Yield: 74%; 123–126 °C; IR (KBr) cm^−1^: 3423, 1369, 1219, 1171, 979. ^1^H-NMR (400.1 MHz, CDCl_3_) δ (ppm): 8.19 (dd, *J* = 4.8 and 1.3 Hz, 1H, H-3‴); 7.87 (d, *J* = 9.0 Hz, 1H, H-7′); 7.72 (d, *J* = 8.3 Hz; 2H, H-2″ and H-6″); 7.51 (s, 1H, H-2′); 7.51–7.45 (m, 1H, H-5‴); 7.18 (d, *J* = 8.2 Hz, 2H, H-3″ and H-5″); 7.07 (d, *J* = 2.4 Hz, 1H, H-4′); 6.92 (dd, *J* = 9.0 and 2.4 Hz, 1H, H-6′); 6.68–6.59 (m, 2H, H-4‴ and H6‴); 5.01 (dd, *J* = 10.2 and 3.1 Hz, 1H, H-1); 3.81 (s, 3H, 5′-OCH_3_); 3.66–3.49 (m, 4H, H-3⁗ and H-5⁗); 2.88–2.80 (m, 2H, H-2, H-2a⁗ and H-6a⁗); 2.80–2.65 (m, 2H, H-2); 2.63–2.55 (m, 2H, H-2b⁗ and H-6b⁗); 2.31 (s, 3H, 4″-CH_3_). ^13^C-NMR (100.6 MHz, CDCl_3_) δ (ppm): 159.5, 156.3, 148.1, 145.0, 137.6, 135.3, 130.3, 130.1, 129.9 (2C), 126.9 (2C), 123.9, 123.3, 114.7, 113.6 (2C), 107.2, 103.3, 63.8, 63.2, 55.8, 53.1 (2C), 45.4 (2C), 21.6. HRMS calculated for C_27_H_31_N_4_O_4_S [M + H^+^]: 507.2061; Found: 507.2064.

*1-(5-methoxy-1-tosyl-1H-indol-3-yl)-2-morpholinoethan-1-ol* (**4q**)

(**3q**) (327 mg, 0.80 mmol) and sodium borohydride (35 mg, 0.90 mmol) were reacted to give a solid, which was purified by column chromatography on silica gel using dichloromethane/ethyl acetate 5:1 to obtain 220 mg of compound (**4q**) as light brown crystalline plates. Yield: 67%; 130–133 °C; IR (KBr) cm^−1^: 3423, 1365, 1216, 1171, 1115, 830. ^1^H-NMR (400.1 MHz, CDCl_3_) δ (ppm): 7.86 (d, *J* = 9.0 Hz, 1H, H-7′); 7.72 (d, *J* = 8.3 Hz, 2H, H-2″ and H-6″); 7.50 (s, 1H, H-2′); 7.18 (d, *J* = 8.2 Hz, 2H, H-3″ and H-5″); 7.05 (d, *J* = 2.4 Hz, 1H, H-4′); 6.91 (dd, *J* = 9.0 and 2,.4 Hz, 1H, H-6′); 4.97 (dd, *J* = 9.9 and 3.6 Hz, 1H, H-1); 3.80 (s, 3H, 5′-OCH_3_); 3.78–3.69 (m, 4H, H-3⁗ and H-5⁗); 2.79–2.61 (m, 4H, H-2, H-2a⁗ and H-6a⁗); 2.53–2.42 (m, 2H, H-2b⁗ and H-6b⁗); 2.32 (s, 3H, 4″-CH_3_). ^13^C-NMR (100.6 MHz, CDCl_3_) δ (ppm): 156.3, 145.0, 135.3, 130.3, 130.1, 129.9 (2C), 126.9 (2C), 123.9, 123.1, 114.7, 113.6, 103.2, 67.1 (2C), 64.2, 63.0, 55.8, 53.7 (2C), 21.6. HRMS calculated for C_22_H_27_N_2_O_5_S [M + H^+^]: 431.1635; Found: 431.1635.

*1-(1-((5-bromothiophen-2-yl)sulfonyl)-5-methoxy-1H-indol-3-yl)-2-(4-(2-methoxyphenyl)piperazin-1-yl)ethan-1-ol* (**4r**)

(**3r**) (318 mg, 0.60 mmol) and sodium borohydride (24 mg, 0.80 mmol) were reacted to give a gel, which was purified by column chromatography on silica gel using dichloromethane/ethyl acetate 5:1 to obtain 260 mg of compound (**4r**) as a yellow gel. Yield: 75%; product in gel state; IR (KBr) cm^−1^: 3422, 1367, 1219, 1164, 1029. ^1^H-NMR (400.1 MHz, CDCl_3_) δ (ppm): 7.85 (d, *J* = 9.0 Hz, 1H, H-7′); 7.45 (s, 1H, H-2′); 7.39 (bs, 1H, H-2″); 7.13 (s, 1H, H-4′), 7.07–6.91 (m, 5H, H-6′, H-3″, H-3‴, H-4‴, H-5‴); 6.88 (d, *J* = 7.9 Hz, 1H, H-6‴); 5.05 (d, *J* = 8.4 Hz, 1H, H-1); 3.87 (s, 3H, 2‴-OCH_3_); 3.85 (s, 3H, 5′-OCH_3_); 3.70 (bs, 1H, OH); 3.16 (bs, 4H, H-3⁗ and H-5⁗); 3.00 (bs, 2H, H-2a⁗ and H-6a⁗); 2.88–2.61 (m, 4H, H-2, H-2b⁗ and H-6b⁗). ^13^C-NMR (100.6 MHz, CDCl_3_) δ (ppm): 156.7, 152.3, 140.9, 138.6, 133.1, 130.5, 130.4, 130.0, 124.8, 123.5, 123.2, 121.7, 121.1, 118.3, 114.8, 113.9, 111.3, 103.6, 63.7, 63.0, 55.8, 55.4, 53.5 (2C), 50.6 (2C). HRMS calculated for C_26_H_29_BrN_3_O_5_S_2_ [M + H^+^]: 606.0727; Found: 606.0741.

*1-(1-((5-bromothiophen-2-yl)sulfonyl)-5-methoxy-1H-indol-3-yl)-2-morpholinoethan-1-ol* (**4s**)

(**3s**) (364 mg, 0.70 mmol) and sodium borohydride (33 mg, 0.90 mmol) were reacted to give a solid, which was purified by column chromatography on silica gel using dichloromethane/ethyl acetate 5:1 to obtain 231 mg of compound (**4s**) as orange crystalline plates. Yield: 45%; 52–55 °C; IR (KBr) cm^−1^: 3424, 1373, 1220, 1174, 1113, 866, 684. ^1^H-NMR (400.1 MHz, CDCl_3_) δ (ppm): 7.83 (d, *J* = 9.0 Hz, 1H, H-7′); 7.42 (s, 1H, H-2′); 7.38 (d, *J* = 3.8 Hz, 1H, H-2″); 7.10 (s, 1H, H-4′); 7.00–6.90 (m, 2H, H-6′ and H-3″); 5.05 (dd, *J* = 9.4 and 3.2 Hz, 1H, H-1), 4.31 (bs, 1H, OH); 3.83 (s, 3H, 5′-OCH_3_); 3.78 (bs, 4H, H-3⁗ and H-5⁗); 2.85–2.69 (m, 4H, H-2, H-2a⁗ and H-6a⁗); 2.63–2.52 (m, 2H, H-2b⁗ and H-6b⁗). ^13^C-NMR (100.6 MHz, CDCl_3_) δ (ppm): 156.7, 138.5, 133.2, 130.5, 130.2, 129.9, 124.5, 123.5, 121.8, 114.8, 113.8, 103.5, 66.7 (2C), 64.0, 62.8, 55.8, 53.6 (2C). HRMS calculated for C_19_H_22_BrN_2_O_5_S_2_ [M+H^+^]: 501.0148; Found: 501.0168.

*1-(5-methoxy-1-((4-methoxyphenyl)sulfonyl)-1H-indol-3-yl)-2-morpholinoethan-1-ol* (**4t**)

(**3t**) (377 mg, 0.80 mmol) and sodium borohydride (39 mg, 1.00 mmol) were reacted to give a solid, which was purified by column chromatography on silica gel using dichloromethane/ethyl acetate 5:1 to obtain 230 mg of compound (**4t**) as white crystalline plates. Yield: 44%; 64–67 °C; IR (KBr) cm^−1^: 3422, 1366, 1221, 1166, 1115, 1031. ^1^H-NMR (400.1 MHz, DMSO-d_6_) δ (ppm): 7.77 (d, *J* = 9.0 Hz, 2H, H-2″ and H-6″); 7.74 (d, *J* = 9.0 Hz, 1H, H-7′); 7.51 (s, 1H, H-2′); 7.10 (d, *J* = 2.3 Hz, 1H, H-4′); 6.99 (d, *J* = 9.0 Hz, 2H, H-3″ and H-5″); 6.87 (dd, *J* = 9.0 and 2.4 Hz, 1H, H-6′); 5.19 (bs, 1H, OH); 4.93–4.86 (m, 1H, H-1); 3.71 (s, 3H, 4″-OCH_3_); 3.69 (s, 3H, 5′-OCH_3_); 3.48 (bs, 4H, H-3″″ and H-5″″); 2.60 (bs, 2H, H-2a″″ and H-6a″″); 2.43 (bs, 4H, H-2, H-2b″″ and H-6b″″). ^13^C-NMR (100.6 MHz, DMSO-d_6_) δ (ppm): 163.6, 155.8, 130.3, 129.3, 129.0 (2C), 128.6, 125.8, 123.9, 114.9 (2C), 114.1, 113.3, 103.6, 66.1 (2C), 64.2, 63.4, 55.9, 55.5, 53.6 (2C). HRMS calculated for C_22_H_27_N_2_O_6_S [M+H^+^]: 447.1584; Found: 447.1582.

*1-(5-methoxy-1-((4-methoxyphenyl)sulfonyl)-1H-indol-3-yl)-2-(4-(2-methoxyphenyl)piperazin-1-yl)ethan-1-ol* (**4u**)

(**3u**) (353 mg, 0.60 mmol) and sodium borohydride (29 mg, 0.80 mmol) were reacted to give a gel, which was purified by column chromatography on silica gel using dichloromethane/ethyl acetate 5:1 to obtain 185 mg of compound (**4u**) as a yellow gel. Yield: 61%; product in gel state; IR (KBr) cm^−1^: 3423, 1218, 1362, 1171, 1095. ^1^H-NMR (400.1 MHz, CDCl_3_) δ (ppm): 7.86 (d, *J* = 9.0 Hz, 1H, H-7′); 7.79 (d, *J* = 8.1 Hz, 2H, H-2″ and H-6″); 7.53 (s, 1H, H-2′); 7.10 (s, 1H, H-4′); 7.06–6.98 (m, 1H, H-5‴); 6.97–6.90 (m, 3H, H-3‴, H-6′ and H-4‴); 6.90–6.81 (m, 3H, H-6‴, H-3″ and H-5″); 5.22 (s, 1H, OH); 5.11 (d, *J* = 9.8 Hz, 1H, H-1); 3.86 (s, 3H, 2‴-OCH_3_); 3.82 (s, 3H, 4″-OCH_3_); 3.76 (s, 3H, 5′-OCH_3_); 3.18 (bs, 4H, H-3⁗ and H-5⁗); 3.04 (bs, 2H, H-2a″ and H-6a″); 2.96–2.72 (m, 4H, H-2, H-2b‴ and H-6b‴). ^13^C-NMR (100.6 MHz, CDCl_3_) δ (ppm): 163.7, 156.3, 152.3, 140.8, 130.2, 130.0, 129.6, 129.1 (2C), 123.9, 123.3, 123.1, 121.1, 118.3, 114.6, 114.5 (2C), 113.6, 111.3, 103.0, 63.7, 63.0, 55.8, 55.6, 55.4, 53.5 (2C), 50,2 (2C). HRMS calculated for C_29_H_34_N_3_O_6_S [M +H^+^]: 552.2163; Found: 552.2179.

*1-(5-methoxy-1-((4-methoxyphenyl)sulfonyl)-1H-indol-3-yl)-2-(4-(pyridin-2-yl)piperazin-1-yl)ethan-1-ol* (**4v**)

(**3v**) (351 mg, 0.70 mmol) and sodium borohydride (30 mg, 0.80 mmol) were reacted to give a solid, which was purified by column chromatography on silica gel using dichloromethane/ethyl acetate 5:1 to obtain 268 mg of compound (**4v**) as yellow crystalline plates. Yield: 67%; 97–100 °C; IR (KBr) cm^−1^: 3422, 1367, 1219, 1164, 979. ^1^H-NMR (400.1 MHz, CDCl_3_) δ (ppm): 8.19 (d, *J* = 2.3 Hz, 1H, H-3‴); 7.86 (d, *J* = 9.0 Hz, 1H, H-7′); 7.77 (d, *J* = 8.2 Hz, 2H, H-2″); 7.51 (s, 1H, H-2′); 7.50–7.44 (m, 1H, H-5‴); 7.08 (d, *J* = 2.3 Hz, H-4′); 6.92 (dd, *J* = 9.0 and 2.4 Hz, 1H, H-6′); 6.83 (d, *J* = 8.2 Hz, 2H, H-3″); 6.67–6.59 (m, 2H, H-4‴, H-6‴); 5.04 (d, *J* = 10.1 and 2.9 Hz, 1H, H-1); 3.81 (s, 3H, 5′-OCH_3_); 3.75 (s, 3H, 4″-OCH_3_); 3.67–3.50 (m, 4H, H-3⁗ and H-5⁗); 2.90–2.82 (m, 2H, H-2a⁗ and H-6a⁗); 2.82–2.66 (m, 2H, H-2); 2.66–2.57 (m, 2H, H-2b⁗ and H-6b⁗). ^13^C-NMR (100.6 MHz, CDCl_3_) δ (ppm): 163.8, 159.4, 156.3, 148.0, 137.7, 130.3, 130.1, 129.7, 129.1 (2C), 123.9, 123.1, 114.7, 114.5 (2C), 113.7, 113.6, 107.3, 103.2, 63.8, 63.2, 55.9, 55.7, 53.1 (2C), 45.3 (2C). HRMS calculated for C_27_H_31_N_4_O_5_S [M + H^+^]: 523.2010; Found: 523.2013.

### 3.2. Radioligand Binding Assay at the 5-HT_6_ Receptor

The affinity of compounds toward 5-HT_6_ receptor was evaluated utilizing membranes from HEK-293 cells expressing human 5-HT_6_ receptors and [^125^I]-SB-258585 as iodinated specific radioligand (4-iodo-*N*-[4-methoxy-3-(4-methyl-piperazin-1-yl)-phenyl]-benzenesulphonamide; K_d_= 1.3 nM; 2200 Ci/mmol). Competitive inhibition assays were performed according to standard procedures. Briefly, fractions of 45 µL of diluted 5-HT_6_ membrane preparation were incubated at 27 ºC for 180 min with 25 µL of [^125^I]-SB-258585 (0.2 nM) and 25 µL of WGA PVT SPA beads (4mg/mL) in the presence of increasing concentrations (10^−11^ to 10^−4^M) of the competing drug (5 μL) or DMSO, in a final volume of 100 μL of assay buffer (50 mM Tris, 120 mM NaCl, pH 7.4). Nonspecific binding was determined by radioligand binding in the presence of a saturating concentration of 100 μM of clozapine. The binding of [^125^I]-SB-258585 to 5-HT_6_ receptors directly correlated to an increase in the signal that was read on Perkin Elmer Topcount NXT HTS. All compounds were tested at eight concentrations in triplicate. Clozapine was used as an internal standard for comparison. The data generated were analyzed using Prism software (GraphPad Inc). A linear regression line of data points was plotted from which the concentration of the competing ligand, which displaces 50% of the specific binding of the radioligand (IC_50_ value), was determined and the Ki value was calculated based upon the Cheng–Prusoff equation: K_i_ = IC_50_/(1 + L/K_D_), where L is the concentration of free radioligand used in the assay and K_D_ is the dissociation constant of the radioligand for the receptor.

### 3.3. Docking and Molecular Dynamics (MD) Simulations Studies of Compounds ***4d***, ***4l*** and ***PUC-10***

The 5-HT_6_ receptor model previously reported by our group was used as a starting point [17]. Molecular docking was performed using OEDocking suite v3.3.0.3 (OpenEye Scientific Software, Inc., Santa Fe, NM, USA) [36]. First, the receptor was prepared using the MakeReceptor GUI v3.3.0.3: a box with dimensions 32 × 28 × 29 Å was generated covering key residues reported to be involved in ligand recognition, namely Asp106 (3.32), Val107 (3.33), Cys110 (3.36), Val189 (5.39), Ala192 (5.42). Ser193 (5.43), Thr196 (5.46), Trp281 (6.48), Phe285 (6.52) and Asn288 (6.55) [17,37]. A site shape potential was set up, obtaining an outer contour of 2611 Å; no constraints were defined. The ligands were constructed using MarvinSketch v19.18.0 (ChemAxon Ltd., Budapest, Hungary) and exported as SMILES. The stereocenters were enumerated using Flipper (part of OMEGA distribution) and multiconformer libraries of compounds were prepared using OMEGA v3.1.0.3 (OpenEye Scientific Software, Inc., Santa Fe, NM, USA) [38,39]. QUACPAC v2.0.0.3 (OpenEye Scientific Software, Inc., Santa Fe, NM, USA) was used to assign AM1BCC charges to the library of conformers [40,41,42]. Finally, compounds were docked using FRED v3.3.0.3 (OpenEye Scientific Software, Inc., Santa Fe, NM, USA) and the best-scoring conformation according to the ChemGauss4 score of each compound was extracted for further analysis [43,44,45]. Graphical representations of docking poses were generated using PyMOL v2.4.1 (Schrödinger, LLC, Cambridge, MA, USA).

For MD simulations, ligand–receptor complexes were inserted into the POPC membrane, solvated, and ionized up to a concentration of 0.15 M NaCl, using the Membrane Builder module from the CHARMM-GUI web server [46,47]. The protein orientation was defined based on the OPM database. The initial system size was about 100 × 100 × 120 Å and consisted approximately of 111,000 atoms. All MD simulations were performed using the NAMD package v2.14 [48]. The simulation systems were first relaxed with 2000 steps of minimization followed by gradual heating from 0 to 310 K by running short MD simulations of 500 steps every cycle. The systems were then fixed, except for the lipid tails, which were left to equilibrate for 250 ps. The simulation was switched to NPT conditions and was further equilibrated for 2.5 ns while constraining the protein with an initial force constant of 10 kcal/(mol·Å^2^) and gradually decreasing to 8, 6, 4, 2, 1, 0.5, 0.05 kcal/(mol·Å^2^) every 250 ps of the MD simulation. Finally, the systems were run without any constraints for 80 ns. Proteins, lipids, and ions were described by the CHARMM36 force field and the parameters for ligands were obtained using the antechamber option available in the *Membrane Builder* interface [49,50]. The Van der Waals interactions were calculated applying a cutoff distance of 12 Å and switching the potential from 10 Å. A timestep of 2 fs was used in the production phase and PME (Particle Mesh Ewald) was employed for the treatment of long-range electrostatic interactions [51]. The temperature was maintained at 310 K using Langevin dynamics. The Nose–Hoover–Langevin piston method was used to control the target pressure (1 atm) with the LangevinPistonPeriod set to 200 fs and LangevinPistonDecay set to 50 fs. The results were analyzed using Visual Molecular Dynamics (VMD) software and PyMOL suite [52,53]. The last 30 ns of each simulation was extracted for analysis and clustered using the hierarchical agglomerative algorithm available in cpptraj [30]. The representative frame of the most populated cluster was then analyzed using PLIP web server [31]. 

### 3.4. CoMFA and CoMSIA Studies

#### 3.4.1. Selection of Conformers and Molecular Alignment

CoMFA and CoMSIA studies were performed with SYBYL-X v1.2 (Tripos International, St. Louis, MO, USA) software installed in a Windows 10 environment on a PC with an Intel Core i7 CPU. The alignment of the best docking poses was used as a basis for the formulation of the models. For the calculation of the potentials, each compound was assigned MMFF94 loads. The compounds were divided into training and test sets in order to test both the quality of the internal and external predictive capacities of the models. Fifteen compounds were used as test sets (equivalent to 32% of the total) and 32 compounds as training sets (equivalent to 68% of the total). The Ki values were converted to pKi (−logKi).

#### 3.4.2. CoMFA and CoMSIA Field Calculation

To derive the CoMFA and CoMSIA descriptor fields, the aligned training set molecules were placed in a three-dimensional cubic lattice with a grid spacing of 2Å in the x, y, and z directions such that the entire set was included in it. The CoMFA steric and electrostatic field energies were calculated using a sp^3^ carbon probe atom with a Van der Waals radius of 1.52 Å and a charge of +1.0. The cut-off values for both steric and electrostatic fields were set to 30.0 kcal/mol. For CoMSIA analysis, the standard settings (probe with charge +1.0, radius 1Å, hydrophobicity +1.0, H-bond donating +1.0, and H-bond accepting +1.0 [54]) were used to calculate five different fields: steric, electrostatic, hydrophobic, donor, and acceptor. Gaussian-type distance dependence was used to measure the relative attenuation of the field position of each atom in the lattice and led to a much smoother sampling of the fields around the molecules when compared to CoMFA. The default value of 0.3 was set for attenuation factor α.

#### 3.4.3. Internal Validation and Partial Least Squares (PLS) Analysis 

PLS analysis was used to construct a linear correlation between the CoMFA and CoMSIA descriptors (independent variables) and the activity values (dependent variables) [55]. To select the best model, cross-validation analysis was performed using the leave-one-out (LOO) method (and sample distance PLS [SAMPLS]), which generated the square of the cross-validation coefficient (*q*^2^) and the optimum number of components (N). The non-cross validation was performed with a column filter value of 2.0 to speed up the analysis and reduce the noise. The *q*^2^, which is a measure of the internal quality of the models, was obtained according to the following Equation (1):

(1) $$q^{2}=1-\frac{\sum {\left(y_{i}-y_{pred}\right)}^{2}}{\sum {\left(y_{i}-\bar{y}\right)}^{2}}$$

where $y_{i}$, $\bar{y}$, and $y_{pred}$ are the observed, mean, and predicted activity in the training set, respectively.

## 4. Conclusions

In this work, we reported the synthesis, biological evaluation, and modeling studies of novel *N*-arylsulfonyl indole derivatives as ligands of the 5-HT_6_ receptor. A convenient synthesis was achieved to readily access twenty-two novel diversely substituted derivatives of the extended indole-aryl piperazines. Fifteen of the tested compounds exhibited nanomolar affinity for the 5-HT_6_ receptor, with compound **4d** being the most potent, having a *K*_i_ of 58 nM, which is better than the 5-HT endogenous ligand. Protein–ligand molecular dynamics simulations provided us a deeper insight about the influence of the 5-methoxy or 5-fluorine substitutions in the binding mode of the ligands, suggesting that these C-5 substitutions are detrimental to affinity due to them precluding the ligand from plunging inside the binding site. On the other hand, in the 3D-QSAR studies, the CoMFA and CoMSIA models presented high values of q^2^ (0.653; 0.692) and r^2^ (0.879; 0.970), respectively. The biological activity of the compounds can be explained based on the steric and electronic properties, but mainly on their electronic nature. The information gained in this study will allow the design of novel derivatives with improved activity.

## Figures and Tables

**Table 1:** 5-HT_6_ Binding affinity results for new *N*-arylsulfonylindole derivatives.

The General Structures of the Compounds									
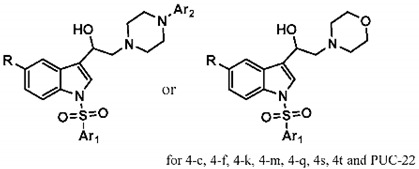									
Code	R	Ar_1_	Ar_2_	*K*_i_ (nM) *^a^*	Code	R	Ar_1_	Ar_2_	*K*_i_ (nM) *^a^*
**4a**	F	4-I-Ph	2-OMe-Ph	390	**4q**	OMe	4-Me-Ph	-	4024
**4b**	F	4-I-Ph	2-py	3629	**4r**	OMe	5-Br-2-thioph	2-OMe-Ph	167
**4c**	F	4-I-Ph	-	12,515	**4s**	OMe	5-Br-2-thioph	-	130
**4d**	F	Naph	2-OMe-Ph	58	**4t**	OMe	4-OMePh	-	7490
**4e**	F	Naph	2-py	801	**4u**	OMe	4-OMePh	2-OMe-Ph	270
**4f**	F	Naph	-	683	**4v**	OMe	4-OMePh	2-py	2185
**4g**	F	Naph	2-pyrim	1444	**PUC7**	H	4-I-Ph	2-OMe-Ph	18.4 *^b^*
**4h**	F	4-OMe-Ph	2-OMe-Ph	850	**PUC9**	H	4-I-Ph	2-py	371 *^b^*
**4i**	F	5-Br-2-thioph	2-OMe-Ph	381	**PUC10**	H	Naph	2-OMe-Ph	14.6 *^b^*
**4j**	OMe	4-I-Ph	2-OMe-Ph	289	**PUC11**	H	Naph	2-py	148 *^b^*
**4k**	OMe	4-I-Ph	-	403	**PUC12**	H	Naph	2-pyrim	603 *^b^*
**4l**	OMe	Naph	2-OMe-Ph	160	**PUC16**	H	4-Me-Ph	2-pyrim	851 *^b^*
**4m**	OMe	Naph	-	247	**PUC22**	H	4-OMePh	-	891 *^b^*
**4n**	OMe	Naph	2-py	153	**PUC24**	H	4-Me-Ph	2-py	479 *^b^*
**4o**	OMe	4-Me-Ph	2-OMe-Ph	972	**PUC32**	H	4-Me-Ph	2-OMe-Ph	13.6 *^b^*
**4p**	OMe	4-Me-Ph	2-py	2195	**5-HT**				75 [18]

*^a^* Displacement of [^125^I]-SB-258585 bound to cloned 5-HT_6_ human receptors stably expressed in HEK-293 cells. IC_50_ values (not shown) were determined in triplicate and *K*_i_ were calculated using Cheng–Prusoff equation [25]. In this assay, the IC_50_ value of Clozapine (Clz) was 12.4 nM; *K*_i_ = 11.9 nM. *^b^ K*_i_ values for unsubstituted derivatives previously reported [17].

**Table 2:** Docking scoring and distance (in Å) of hydrophobic and salt bridge interactions established with residues within the binding site and compounds **PUC10**, **4d** and **4l**.

Hydrophobic Interactions			
Residue	PUC10	4d	4l
Ala83	--	--	3.98
Thr103	--	3.7	--
Val107	3.54	3.72	3.50
	3.99	3.58	3.62
	3.93	3.75	3.67
	--	3.78	--
Pro161	3.69	3.61	3.97
Ala192	3.54	3.58	3.79
Phe284	--	--	3.55
Phe285	--	--	3.64
Phe302	3.74	--	--
Thr306	3.74	--	3.97
Trp307	--	--	3.8
**Salt Bridges**			
Asp106	3.3	4.27	3.56
**FRED ChemGauss 4 Score**			
	−17.27	−16.61	−15.5

**Table 3:** π-stacking interactions established with residues within the binding site and compounds **PUC10**, **4d** and **4l**.

π-Stacking					
Compound	Residue	CentDist	Angle	Offset	Type
**PUC10**	Phe188	4.63	88.3	0.75	T
	--	4.93	88.46	1.88	T
	Phe285	4.89	87.8	1.62	T
**4d**	Trp102	4.15	28.79	1.44	P
	Phe188	5.37	74.16	0.98	T
	Phe285	5.09	78.61	1.34	T
**4l**	Trp102	5.29	78.41	1.23	T
	Phe188	5.34	84.21	1.27	T
	--	5.36	84.38	1.43	T

**Table 4:** Hydrogen bond interactions established with residues within the binding site and compounds **PUC10**, **4d** and **4l**.

Hydrogen Bonds				
Compound	Residue	Dist_H-A	Dist_D-A	Don_Angle
**PUC10**	Ser193	3.18	3.51	101.39
		3.50	3.94	109.52
**4d**	Thr196	3.54	4.00	114.16
	Asn288	1.63	2.63	166.72
**4l**	Asp106	2.89	3.34	109.58
	Tyr310	3.34	3.78	104.37

Interactions calculated using the PLIP web server. Dist_H-A: Distance between H-bond hydrogen and acceptor atom. Dist_D-A: Distance between H-bond donor and acceptor atoms. Don_Angle: Angle at the donor.

**Table 5:** Statistical summary of the best models for CoMFA and CoMSIA.

Models	q^2^	N	SEP	r^2^_ncv_	F	SEE	r^2^_test_	% Contribution	
								Steric	Electrostatic
CoMFA-SE	0.653	2	0.410	0.879	101.243	0.243	0.786	44.9	55.1
CoMSIA-SE	0.692	5	0.409	0.970	161.624	0.128	0.726	39.2	60.8

q^2^ = the square of the LOO cross-validation (CV) coefficient; N = the optimum number of components; SEP = standard error of prediction; SEE is the standard error of estimation of non CV analysis; r^2^_ncv_ is the square of the non CV coefficient; F is the F-test value; r^2^_test_ is the regression coefficient for the molecules in the test set exclusively.

## Data Availability

Data is contained within the article and Supplementary Materials.

## Supplementary Materials

The following are available online at https://www.mdpi.com/article/10.3390/ph14060528/s1, ^1^H-NMR and ^13^C-NMR spectra of final compounds; high-resolution mass spectra of final compounds; radioligand binding studies, dose-response curves for final compounds; tables of QSAR, Tables S1–S3: CoMFA and CoMSIA data.
