# Supplementary material for: Novel N-Arylsulfonylindoles Targeted as Ligands of the 5-HT6 Receptor. Insights on the Influence of C-5 Substitution on Ligand Affinity
Source: Pharmaceuticals (Basel). 2021 Jun 1;14(6):528. doi: 10.3390/ph14060528 (PMC8227400; doi:10.3390/ph14060528)
Supplement: Supplementary file 1 [file pharmaceuticals-14-00528-s001.zip › pharmaceuticals-1215628-supplementary.pdf]

# Novel *N*-Arylsulfonylindoles targeted as ligands of the 5-HT$_6$ Receptor. Insights on the Influence of C-5 substitution on ligand affinity.

Loreto Arrieta Rodríguez,[1,&] Daniela Espinoza Rosales,[1,&] Gonzalo Vera,[1] Young Hwa Cho,[1] David Cabezas,[2,3] David Vásquez-Velásquez,[4] Jaime Mella-Raipán,[2,3] Carlos F. Lagos,[5] Gonzalo Recabarren Gajardo[1,6,*]

[1] Bioactive Heterocycles Synthesis Laboratory (BHSL), Departamento de Farmacia, Facultad de Química y de Farmacia, Pontificia Universidad Católica de Chile, Avda. Vicuña Mackenna 4860, Macul 7820436, Santiago, Chile; liarriet@uc.cl; dmespino@uc.cl; glvera@uc.cl; aycho@uc.cl
[2] Instituto de Química y Bioquímica, Facultad de Ciencias, Universidad de Valparaíso, Av. Gran Bretaña 1111, Valparaíso 2360102, Chile; jaime.mella@uv.cl
[3] Centro de Investigación Farmacopea Chilena (CIFAR), Universidad de Valparaíso, Santa Marta 183, Valparaíso 2360134, Chile; david.cabezas@postgrado.uv.cl
[4] Drug Development Laboratory, Faculty of Chemical and Pharmaceutical Sciences, Universidad de Chile, Sergio Livingstone 1007, Santiago 8380492, Chile. dvazquez@ciq.uchile.cl
[5] Chemical Biology & Drug Discovery Lab, Escuela de Química y Farmacia, Facultad de Medicina y Ciencia, Universidad San Sebastián, Lota 2465, Providencia 7510157, Santiago, Chile; carlos.lagos@uss.cl
[6] Centro Interdisciplinario de Neurociencias, Pontificia Universidad Católica de Chile, Marcoleta 391, Santiago 8330024, Santiago, Chile; grecabarren@uc.cl
[&] These authors contributed equally to this work
* Correspondence: grecabarren@uc.cl; Tel.: +56 2 23541418

**Contents:**

1. Atom numbering for the final compounds…………………………………. S2

2. $^{1}$H-NMR and $^{13}$C-NMR spectra of final compounds............……............... S3

3. High resolution mass spectra of final compounds………………………... S32

4. Radioligand binding studies ..........................................………………...... S43

5. Tables and figures of QSAR ……………………………………................. S47

6. References .......................................................................……...……............ S50

## 1. Atom numbering for the final compounds.

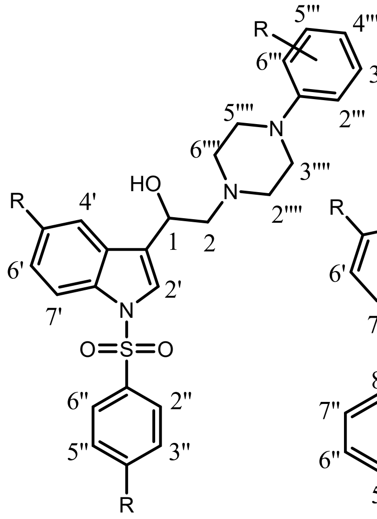


**4a-b, 4h, 4j, 4o-p, 4u-v**

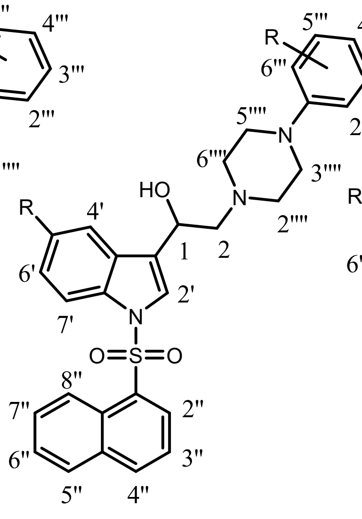


**4d-e, 4g, 4l, 4n**

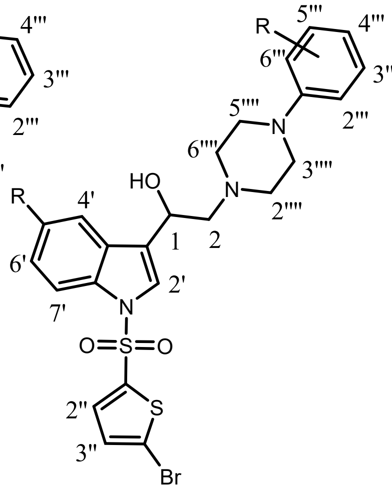


**4i, 4r**

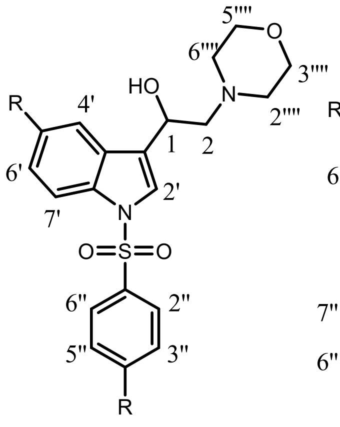


**4c, 4k, 4q, 4t**

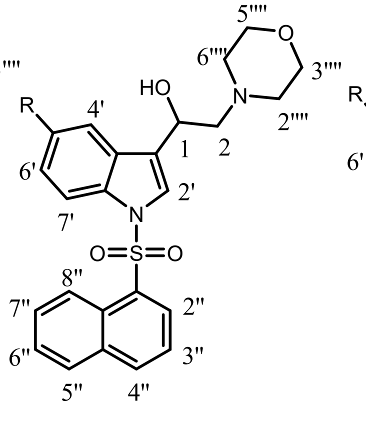


**4f, 4m**

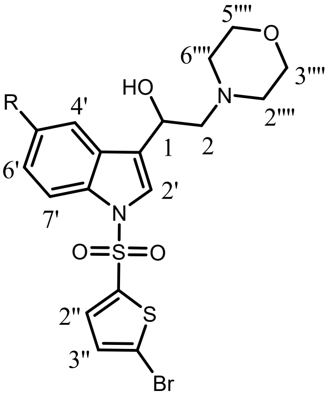


**4s**

## 2. $^{1}$H-NMR and $^{13}$C-NMR spectra of final compounds.

$^{1}$H-NMR (400.1 MHz, $CDCl_3$) for **4a**

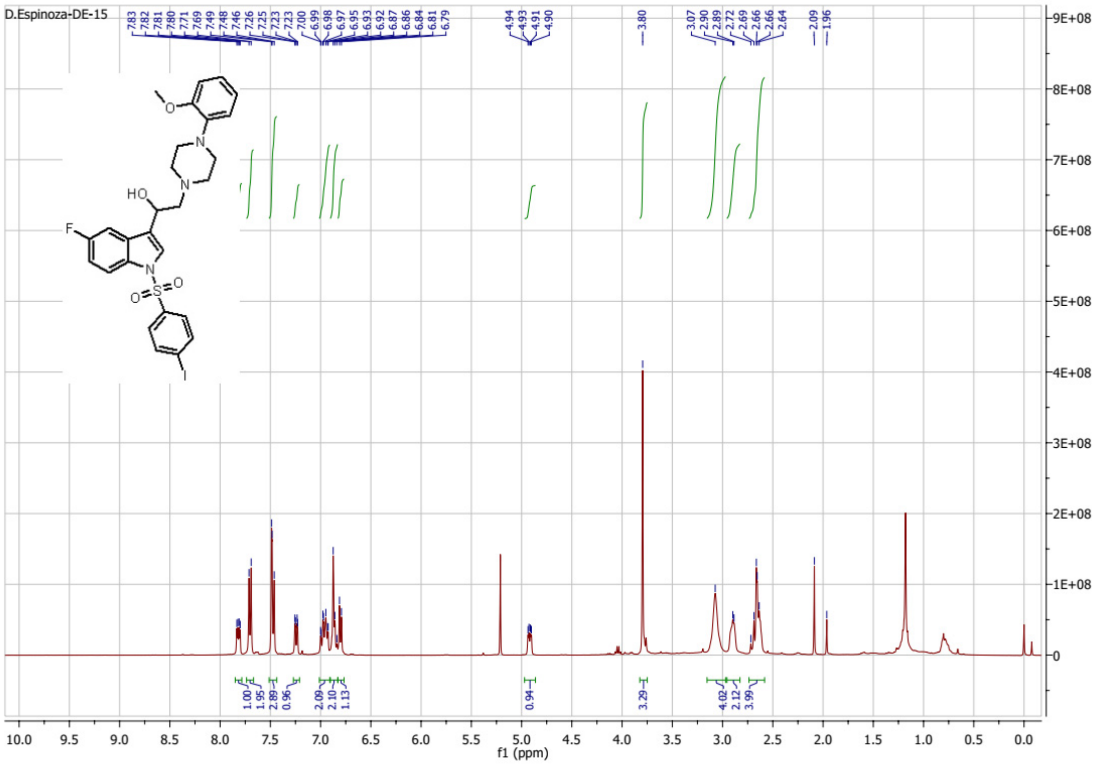


$^{13}$C-NMR (100.6 MHz, $CDCl_3$) for **4a**

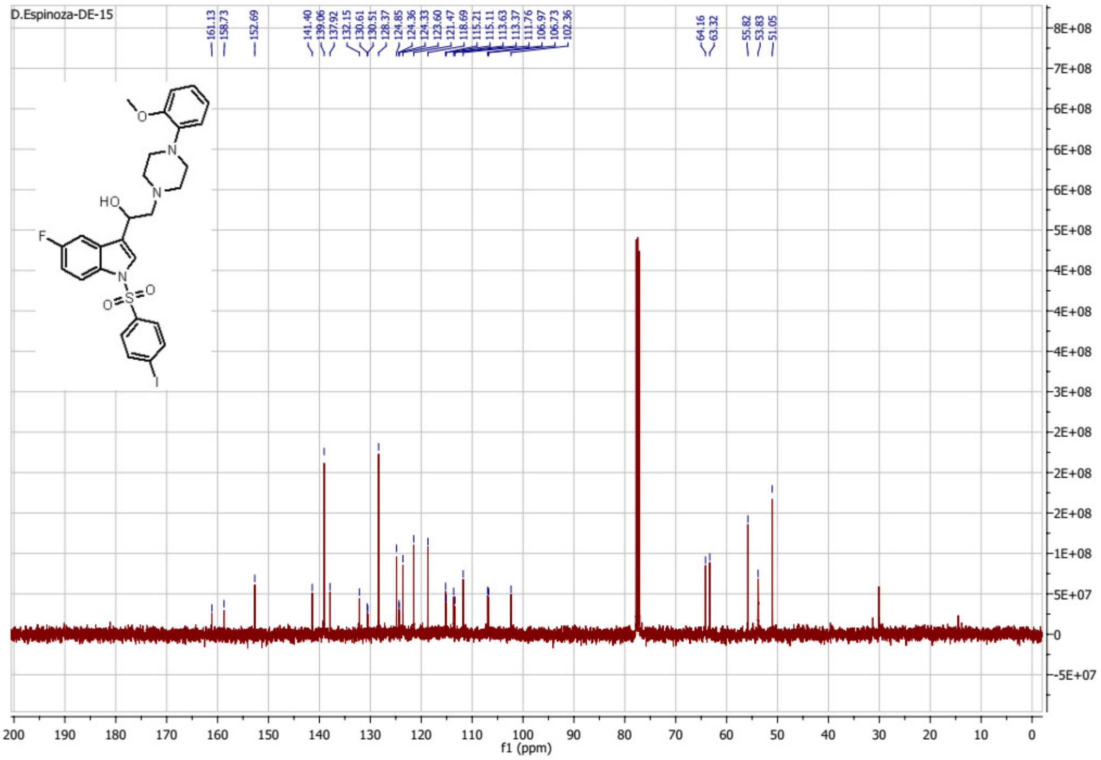

$^{1}H$-NMR (400.1 MHz, $CDCl_3$) for **4b**

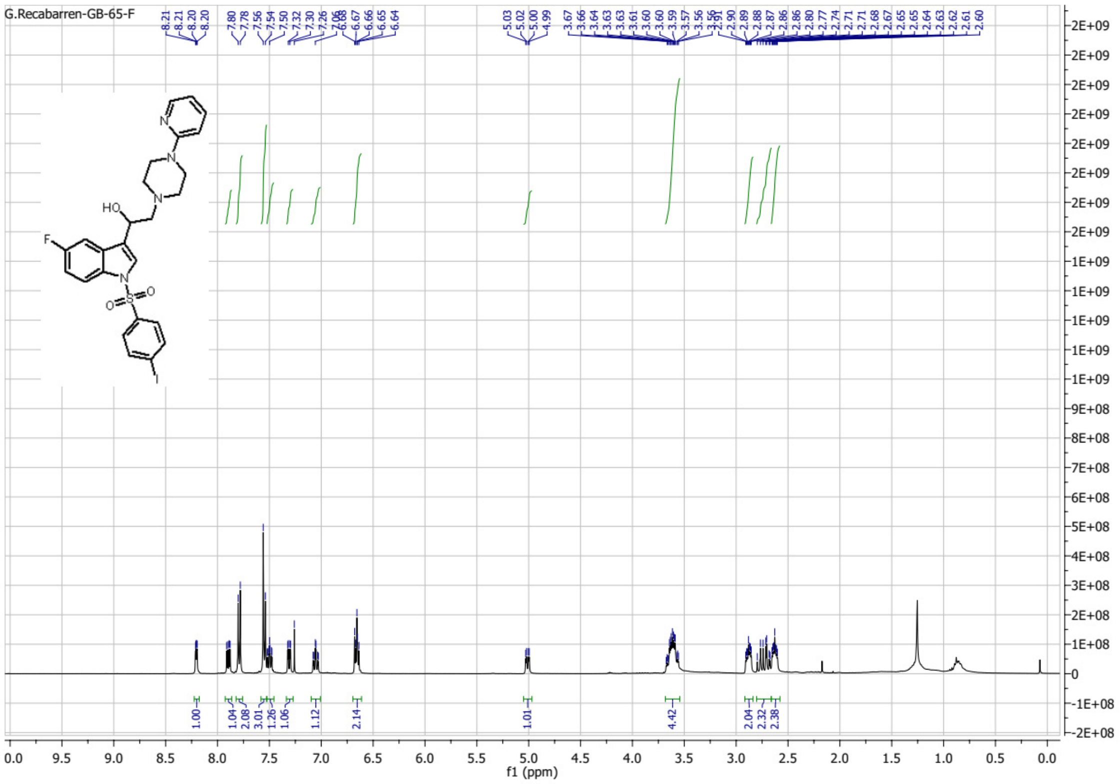

$^{13}C$-NMR (100.6 MHz, $CDCl_3$) for **4b**

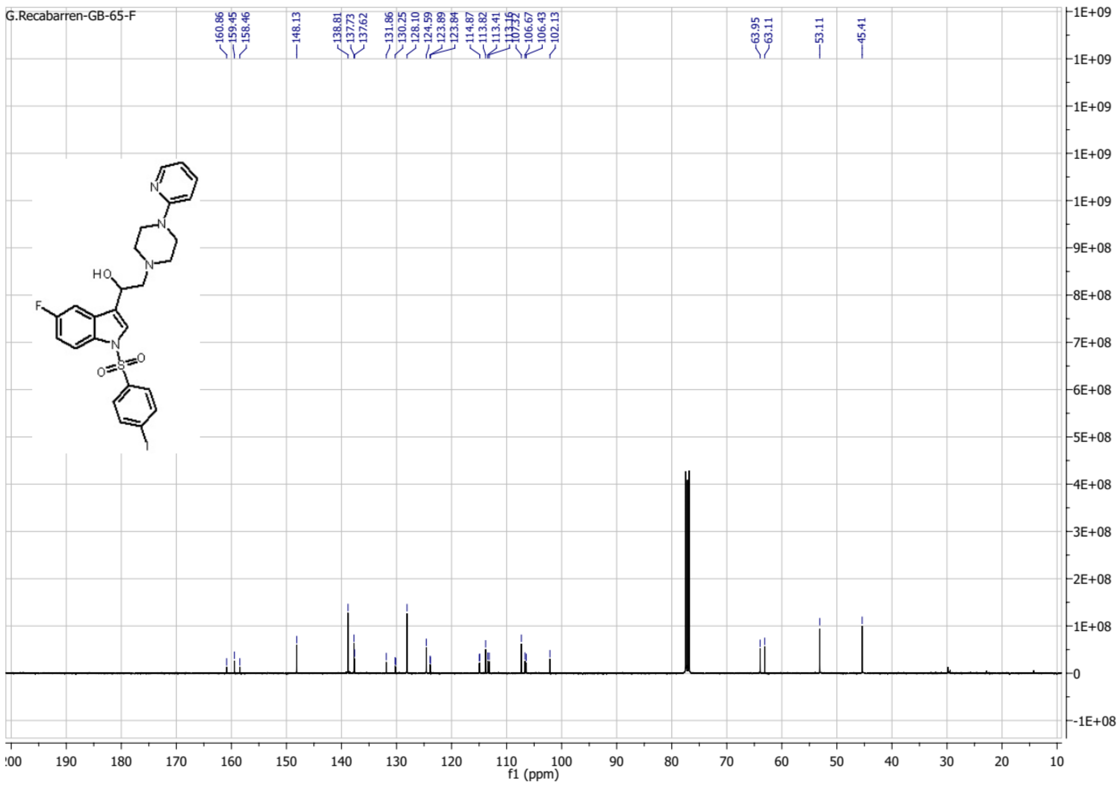

$^{19}F$-NMR (376.5 MHz, $CDCl_3$) for **4b**

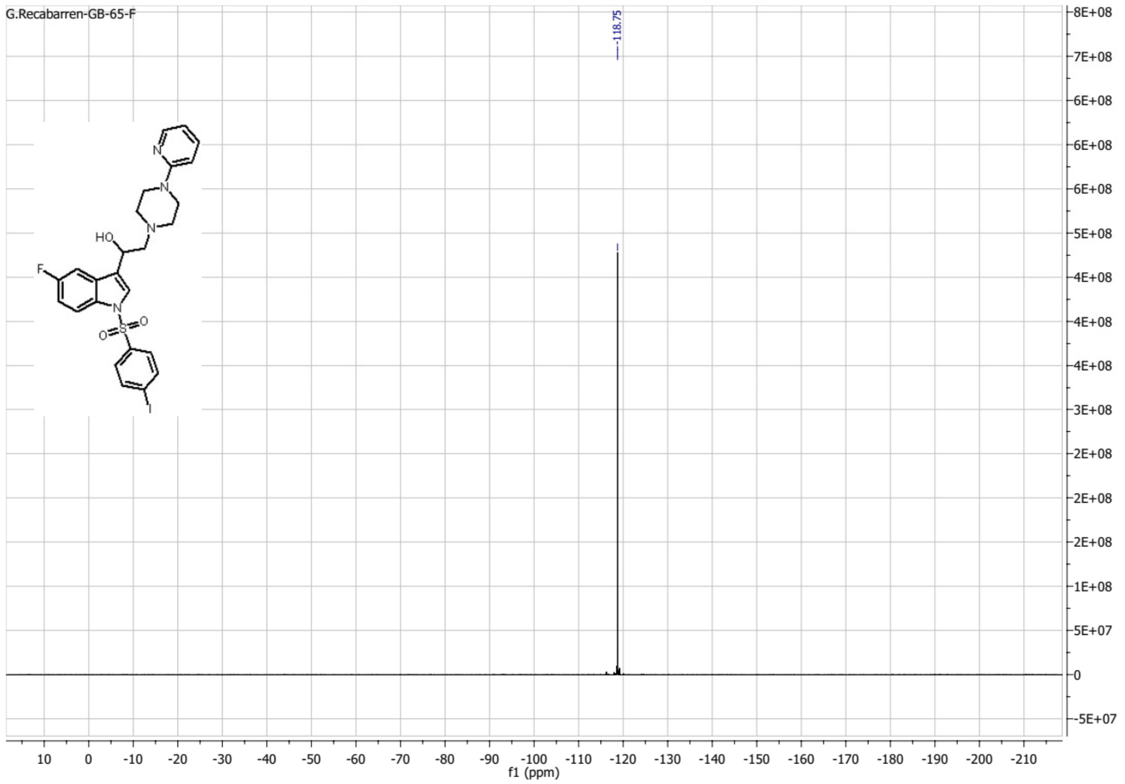

$^{1}H$-NMR (400.1 MHz, $CDCl_3$) for **4c**

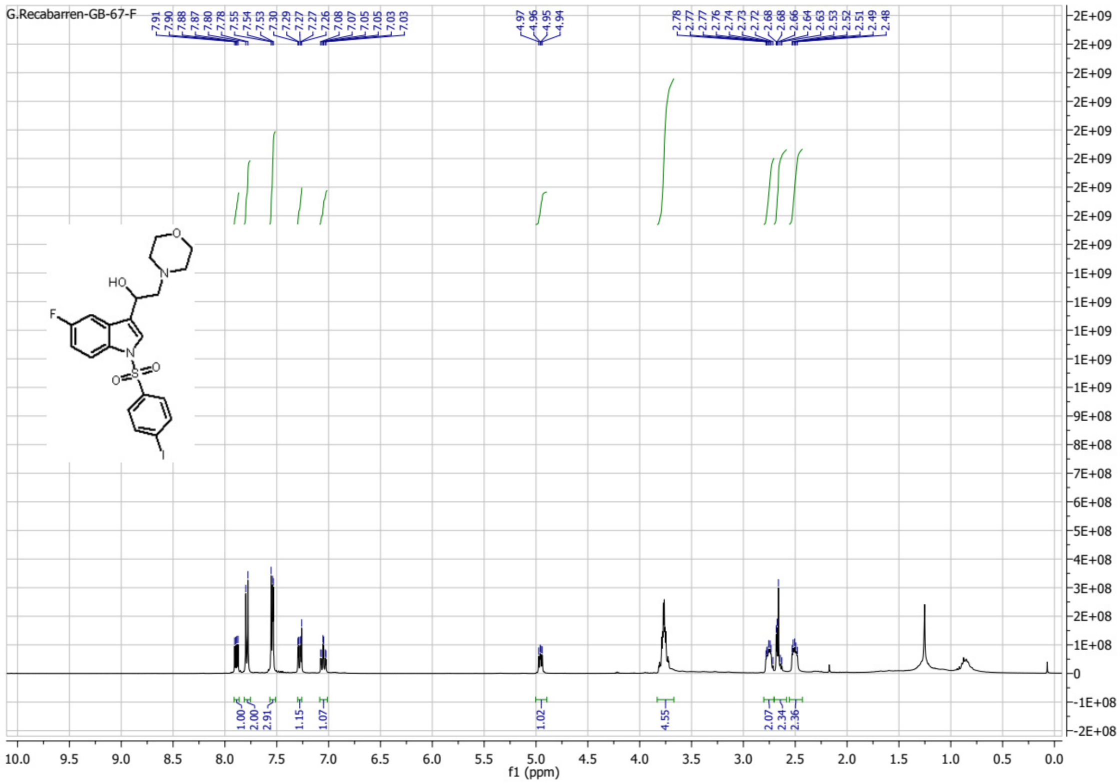

$^{13}C$-NMR (100.6 MHz, $CDCl_3$) for **4c**

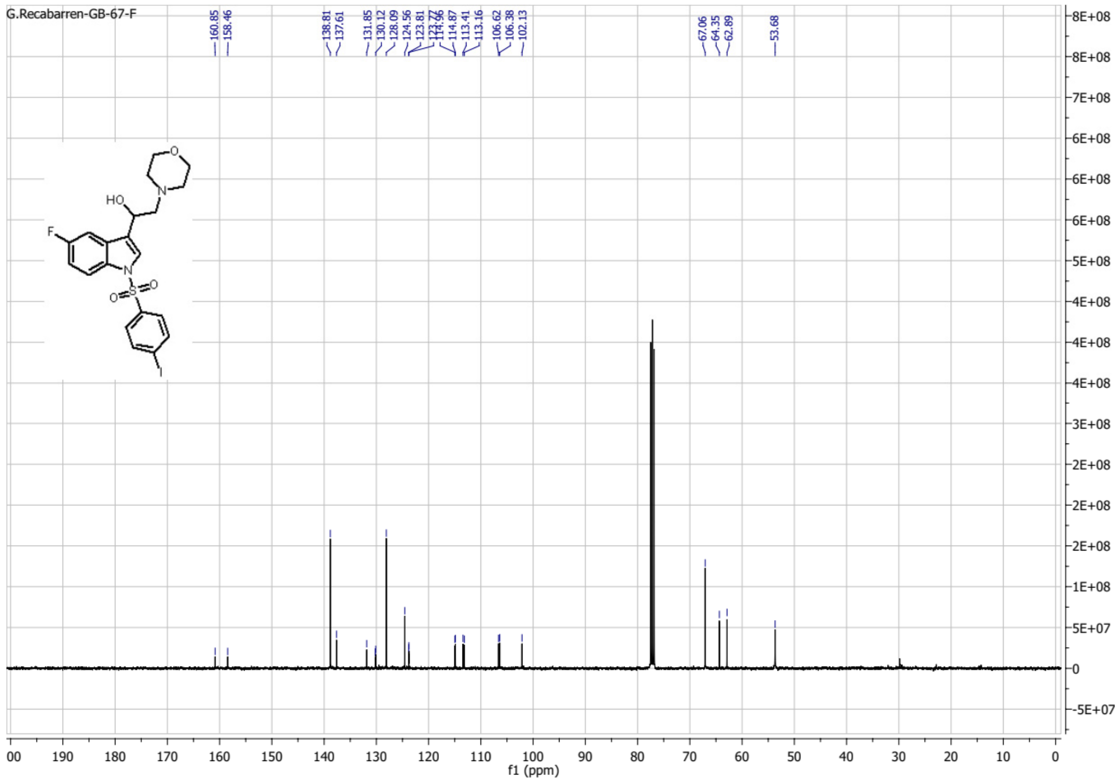

$^{19}$F-NMR (376.5 MHz, $CDCl_3$) for **4c**

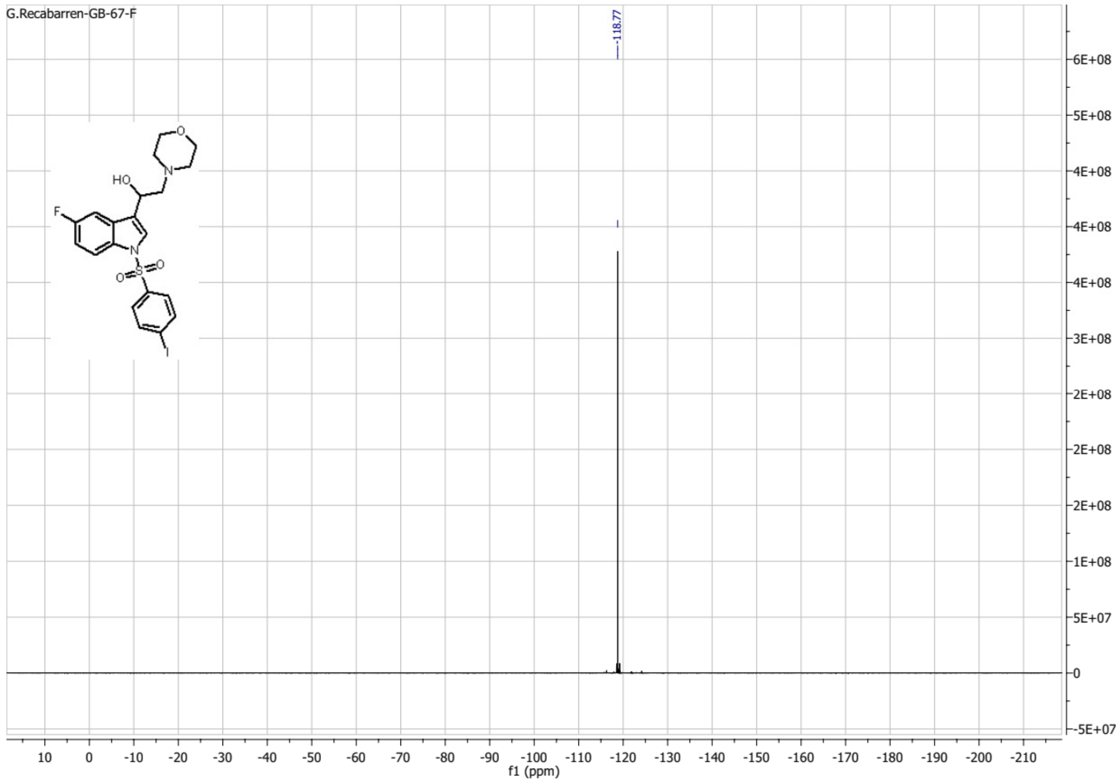

$^{1}$H-NMR (400.1 MHz, $CDCl_3$) for **4d**

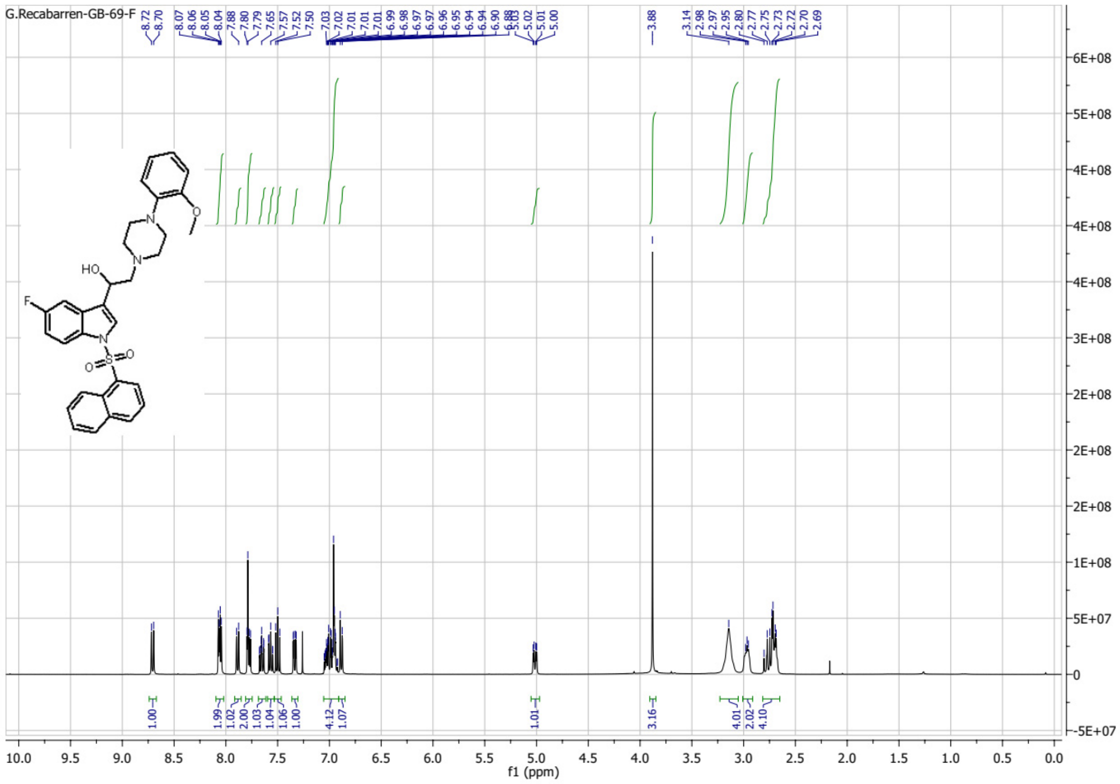

$^{13}$C-NMR (100.6 MHz, $CDCl_3$) for **4d**

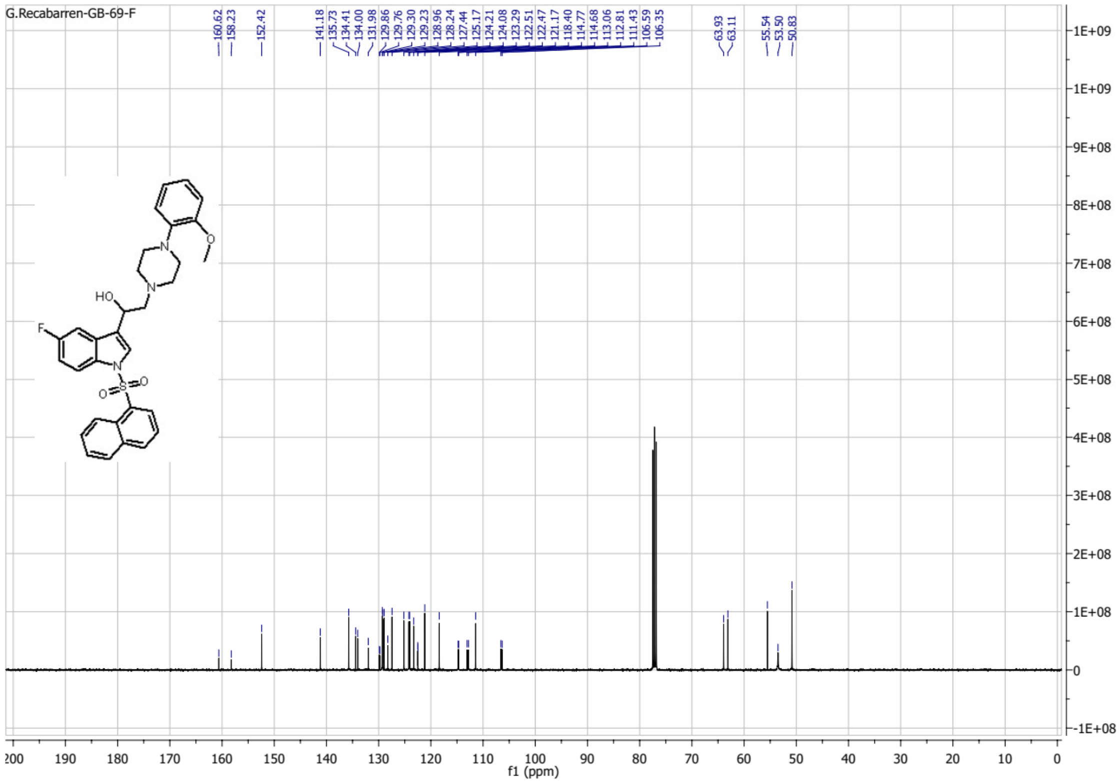

$^{19}F$-NMR (376.5 MHz, $CDCl_3$) for **4d**

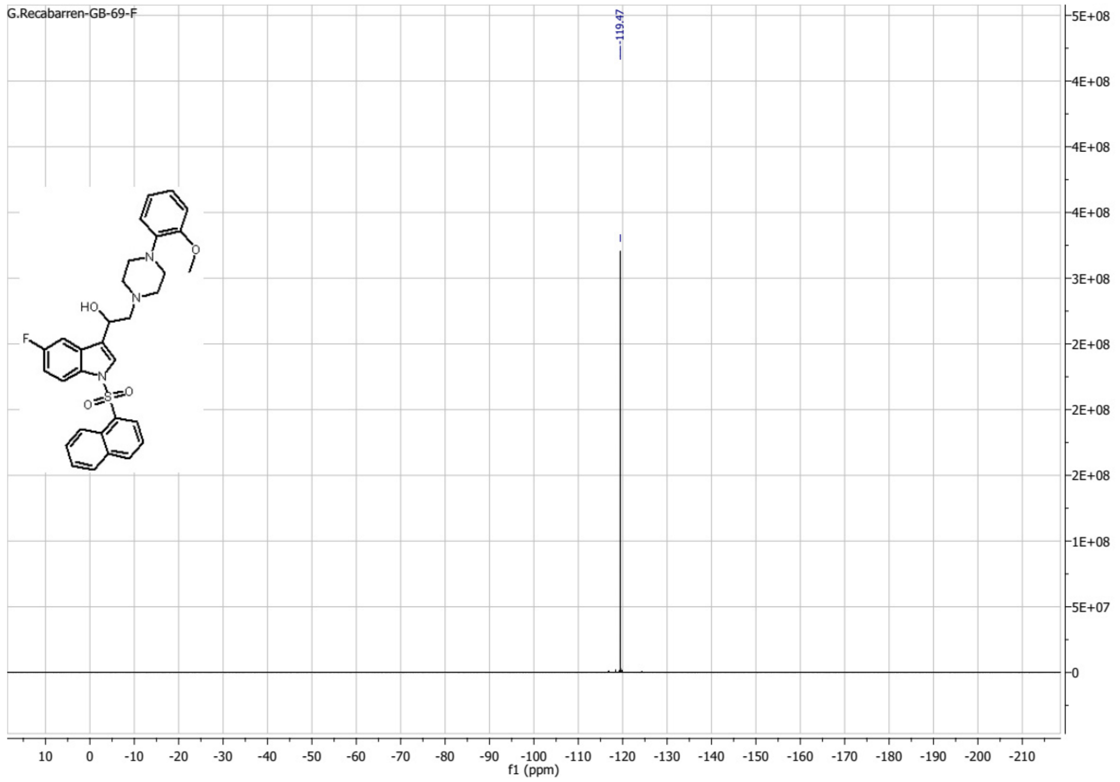

$^{1}$H-NMR (400.1 MHz, $CDCl_3$) for **4e**

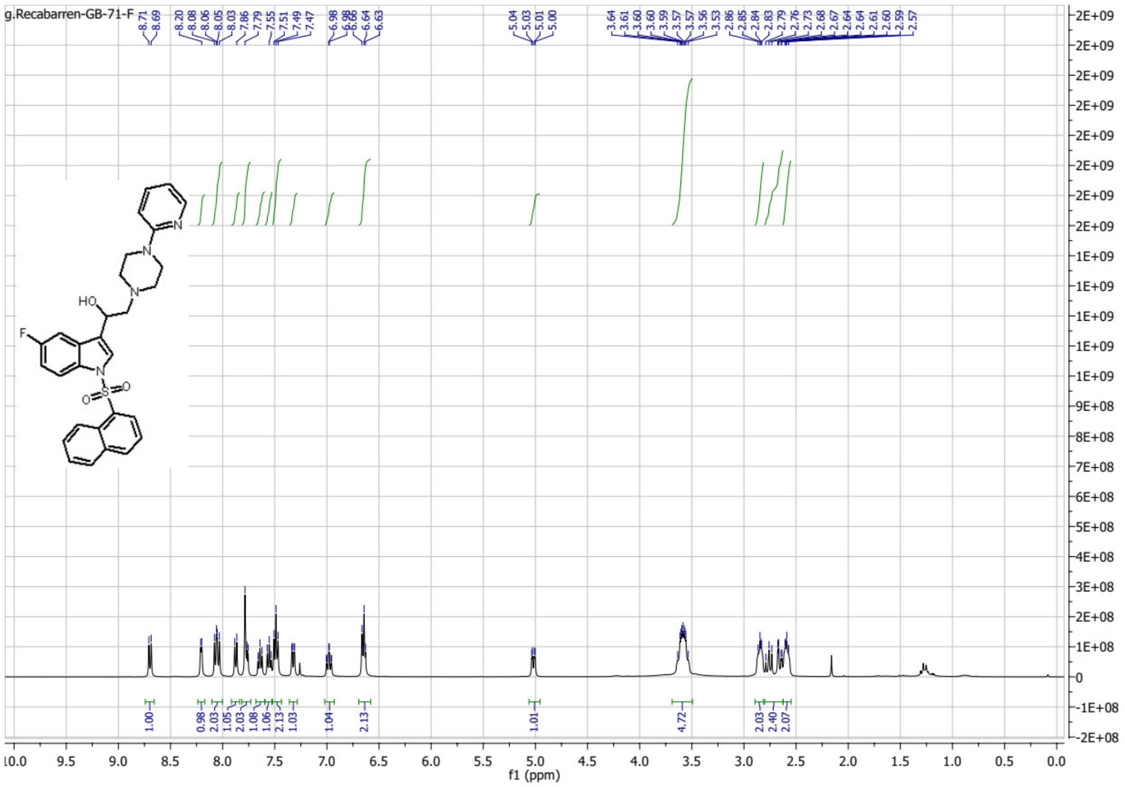

$^{13}$C-NMR (100.6 MHz, $CDCl_3$) for **4e**

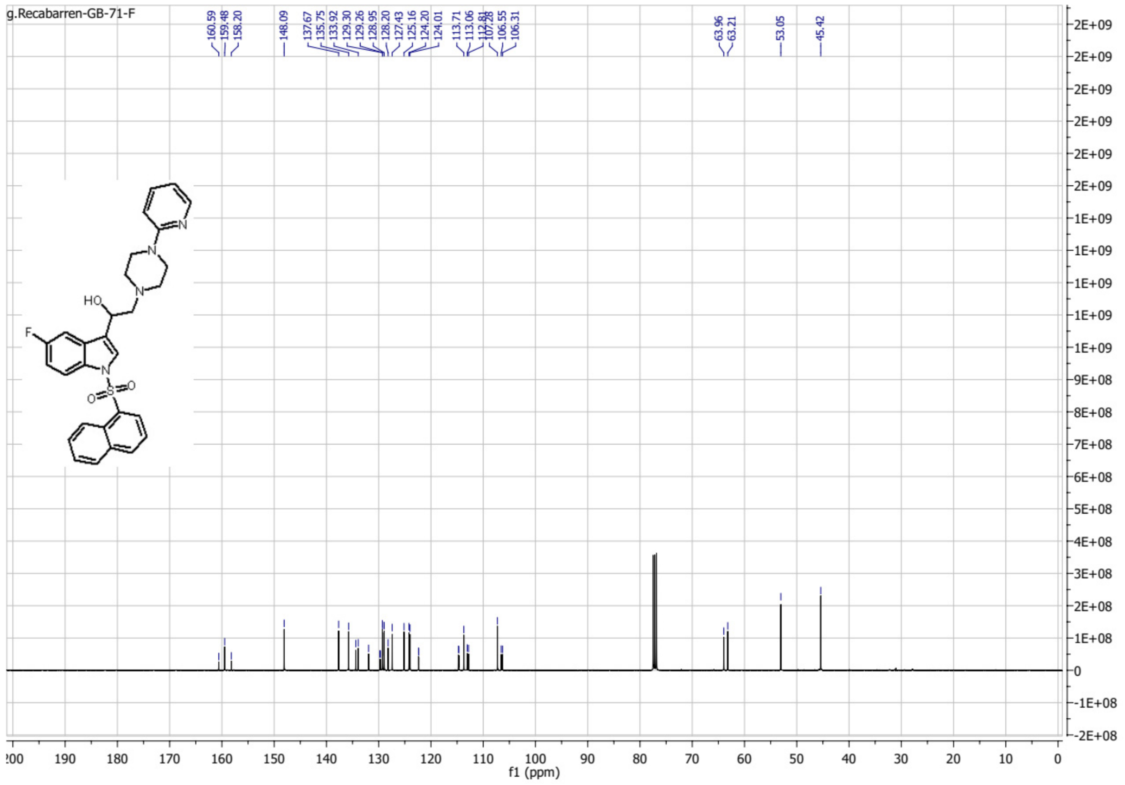

$^{19}$F-NMR (376.5 MHz, $CDCl_3$) for **4e**

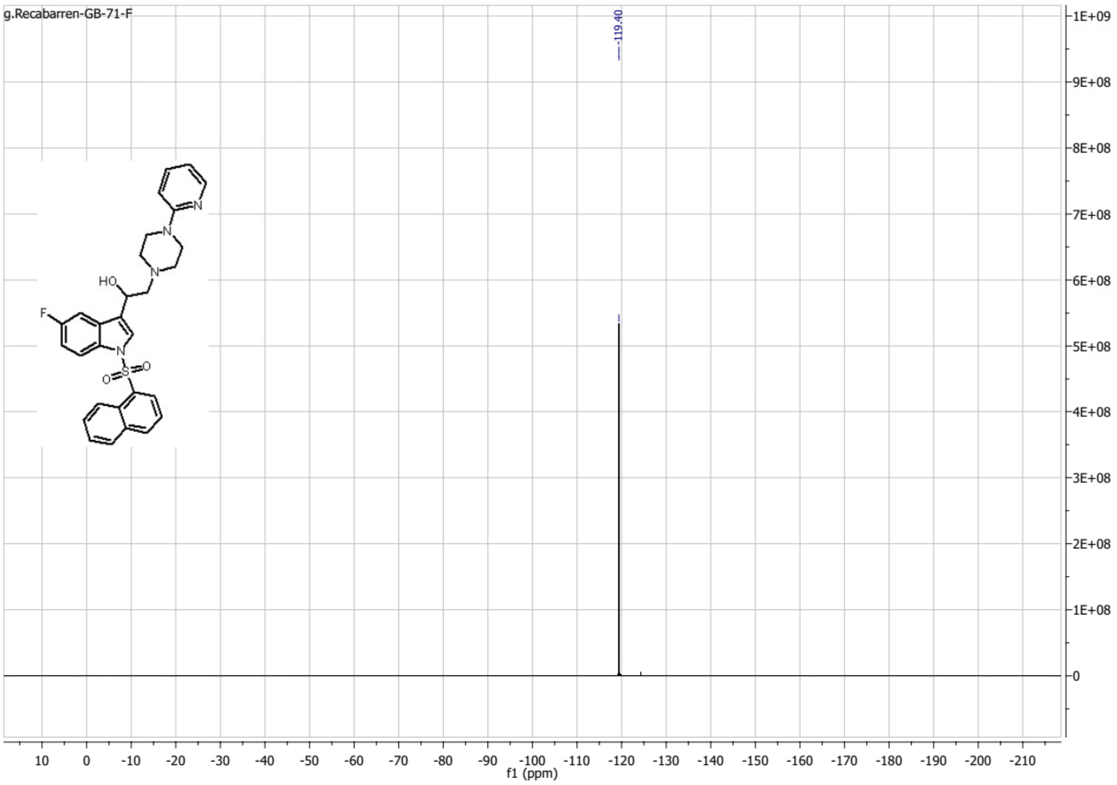

$^{1}H$-NMR (400.1 MHz, $CDCl_3$) for **4f**

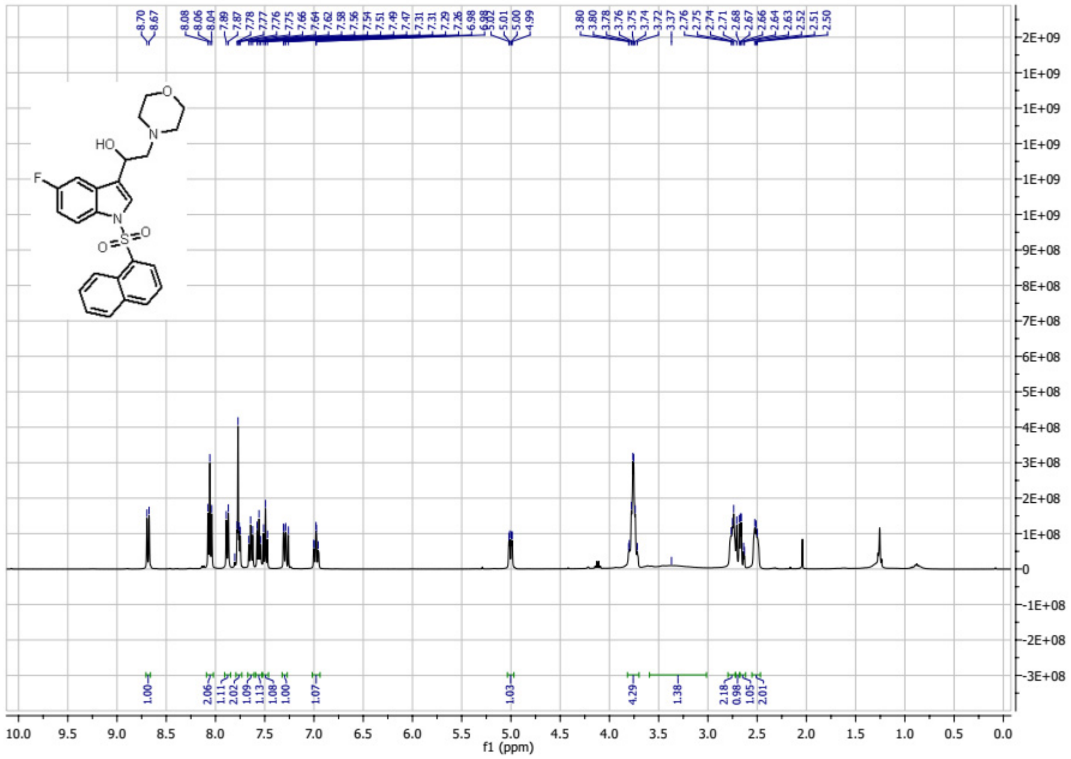

$^{13}C$-NMR (100.6 MHz, $CDCl_3$) for **4f**

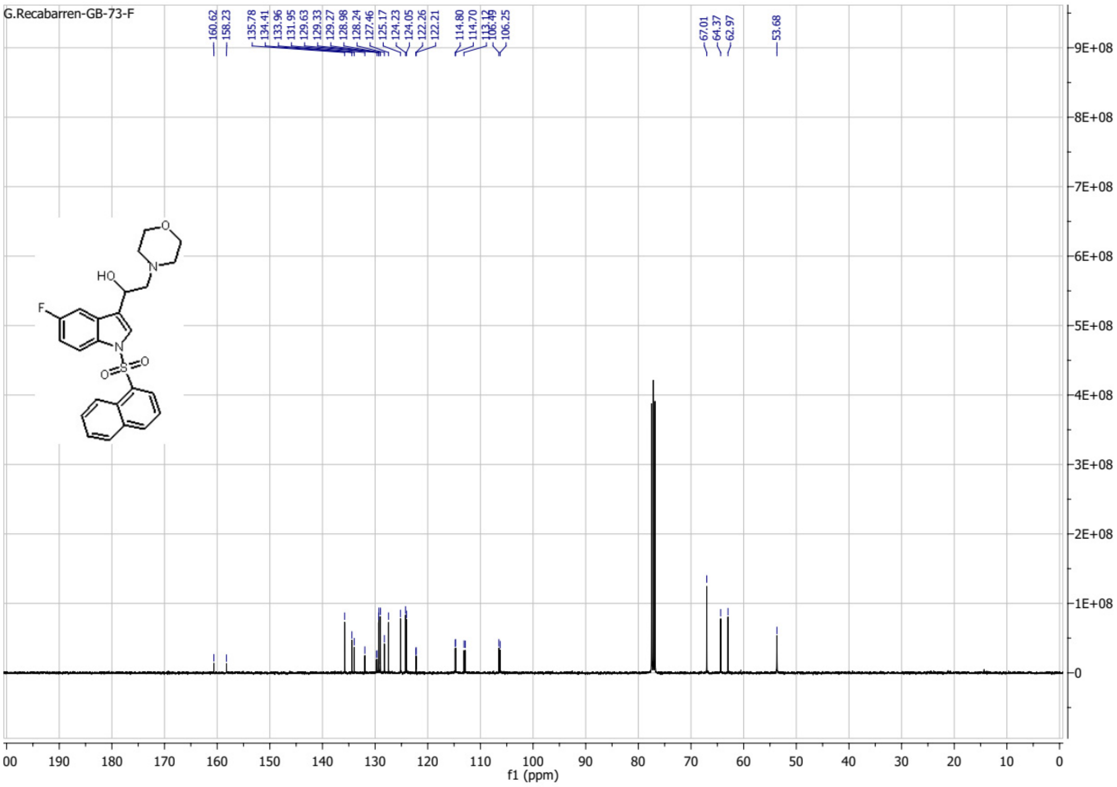

$^{19}$F-NMR (376.5 MHz, $CDCl_3$) for **4f**

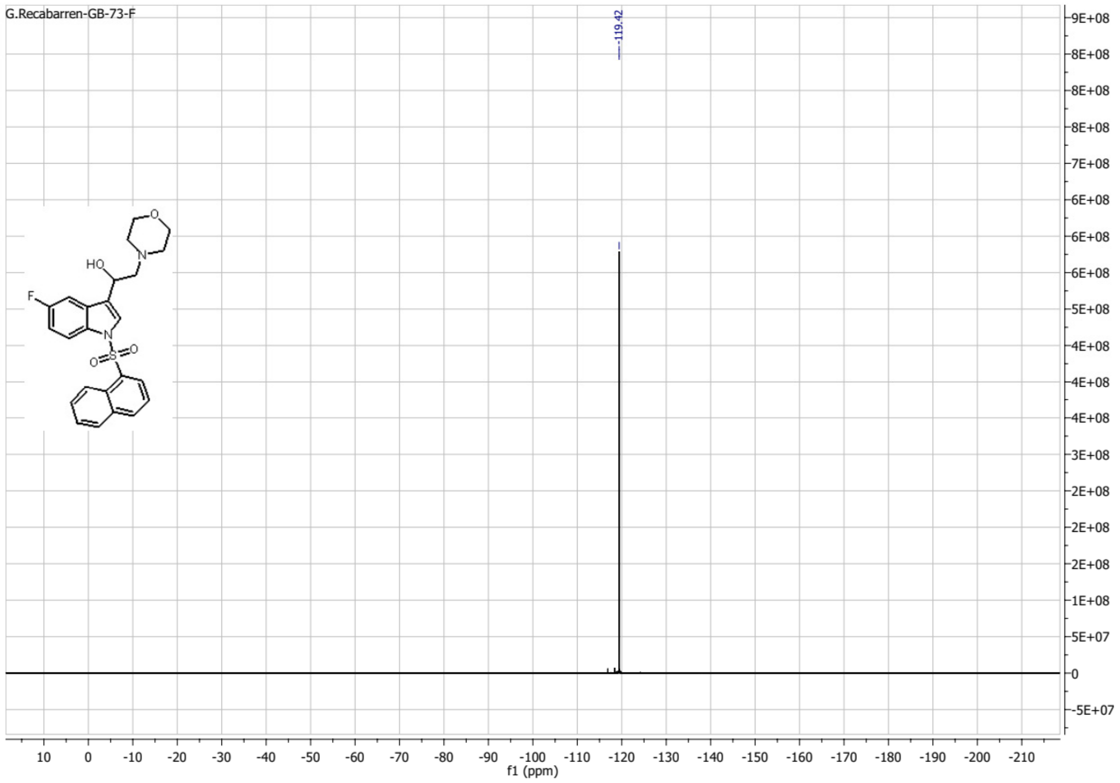

$^{1}$H-NMR (400.1 MHz, $CDCl_3$) for **4g**

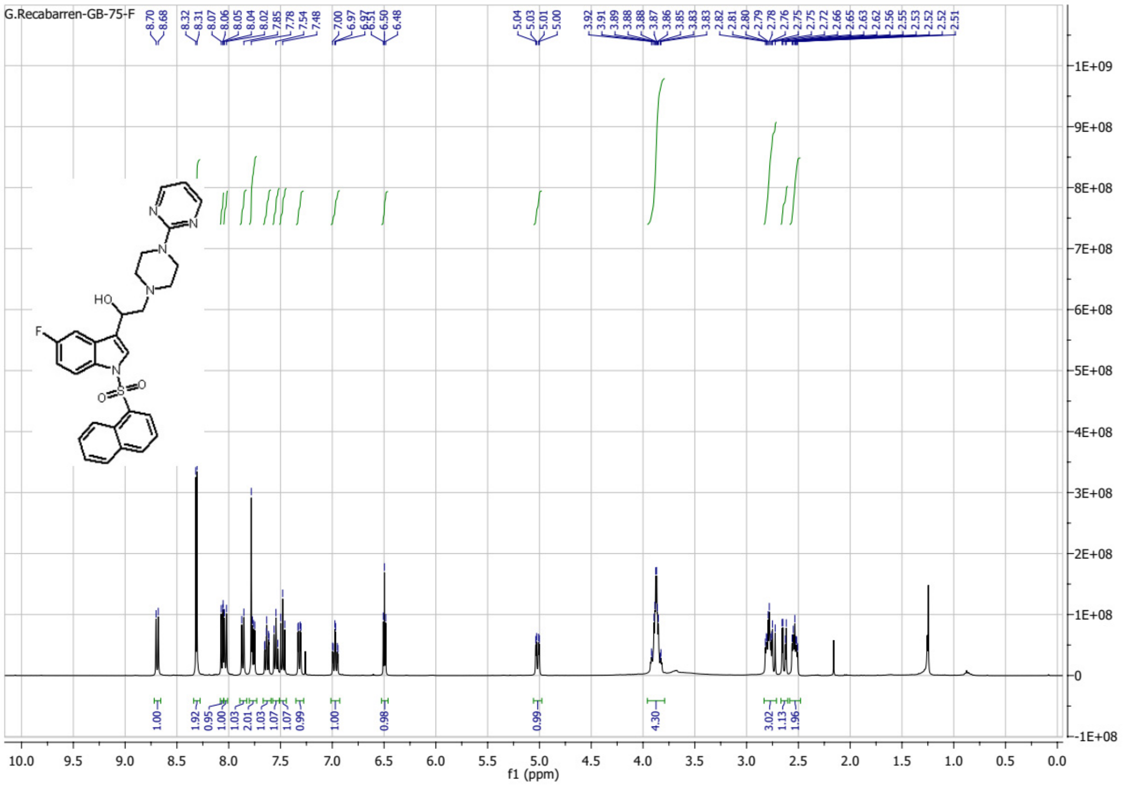


$^{13}$C-NMR (100.6 MHz, $CDCl_3$) for **4g**

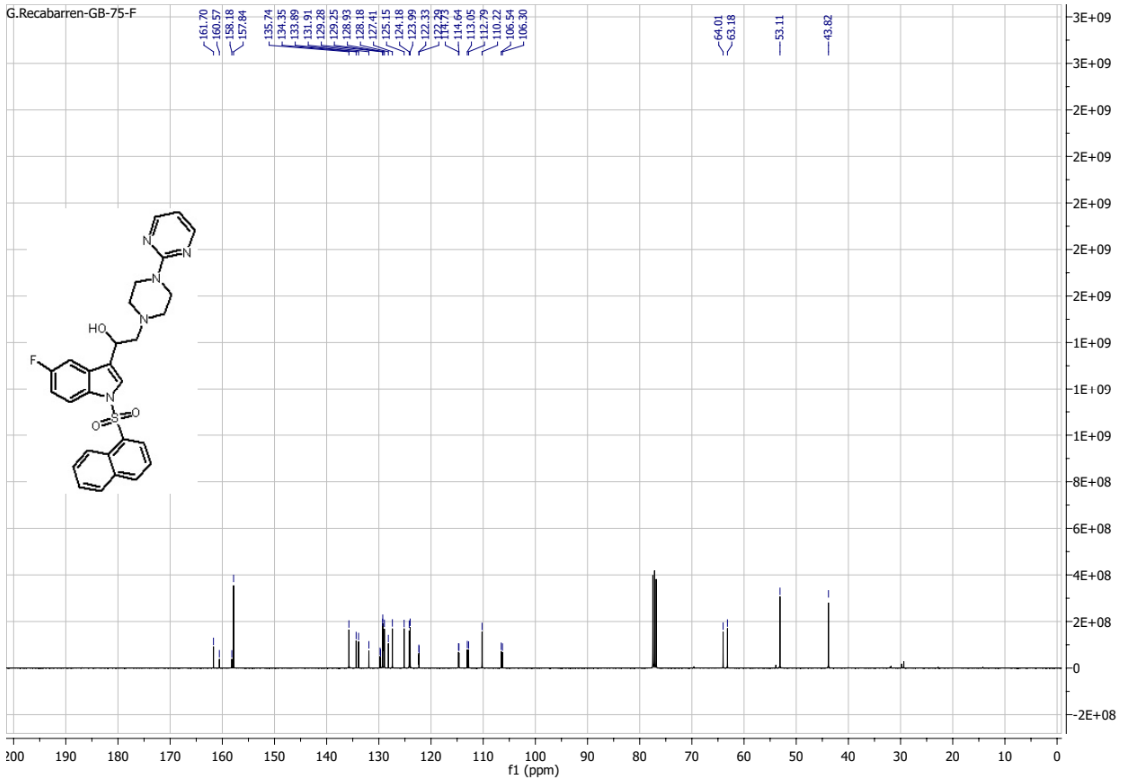

$^{19}$F-NMR (376.5 MHz, $CDCl_3$) for **4g**

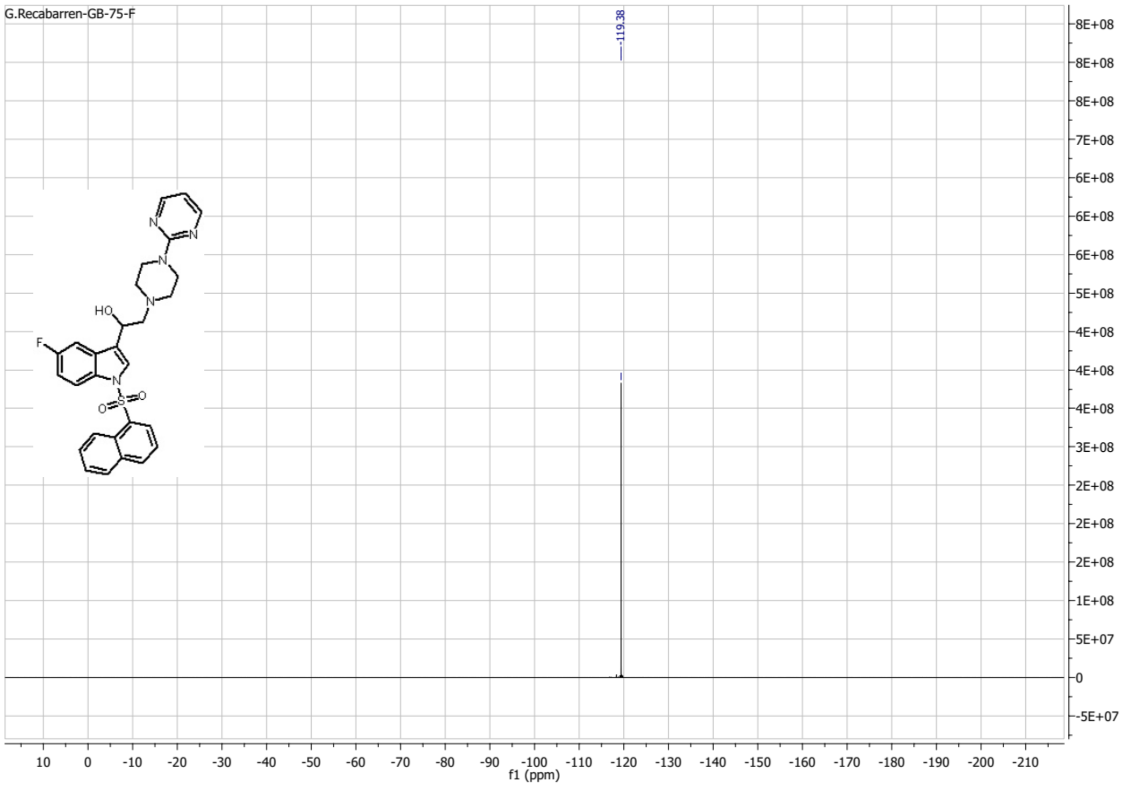

$^{1}$H-NMR (400.1 MHz, $CDCl_3$) for **4h**

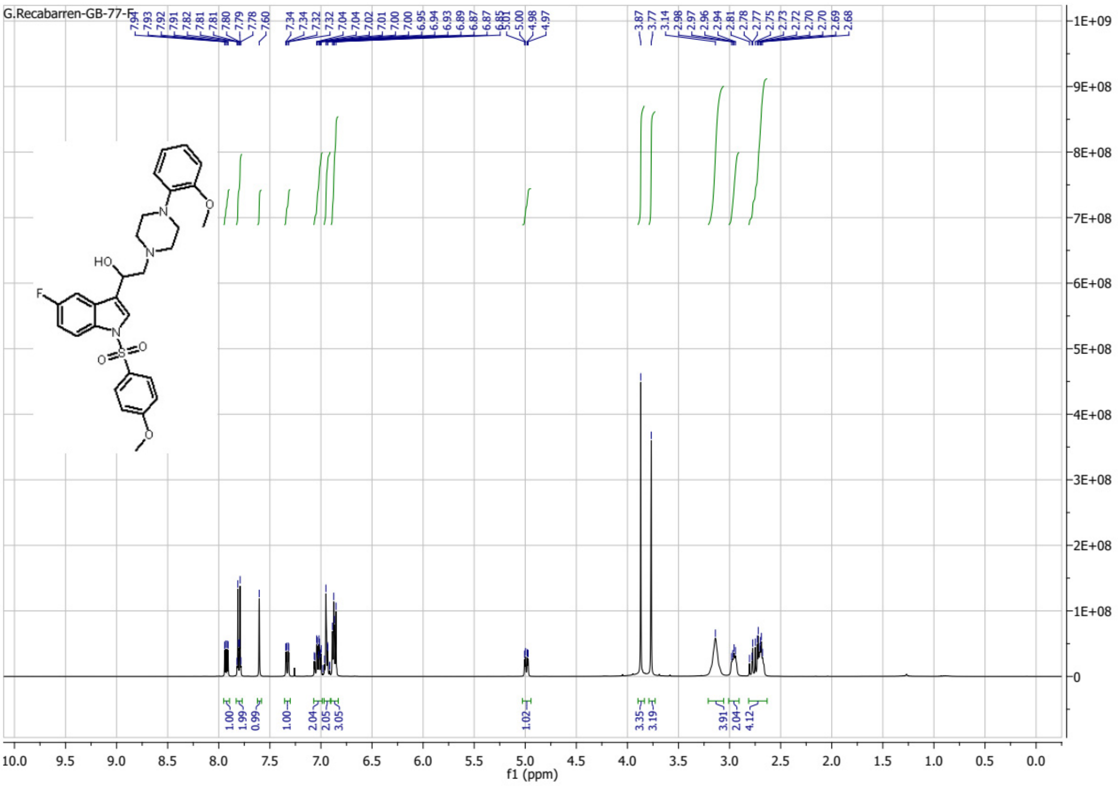


$^{13}$C-NMR (100.6 MHz, $CDCl_3$) for **4h**

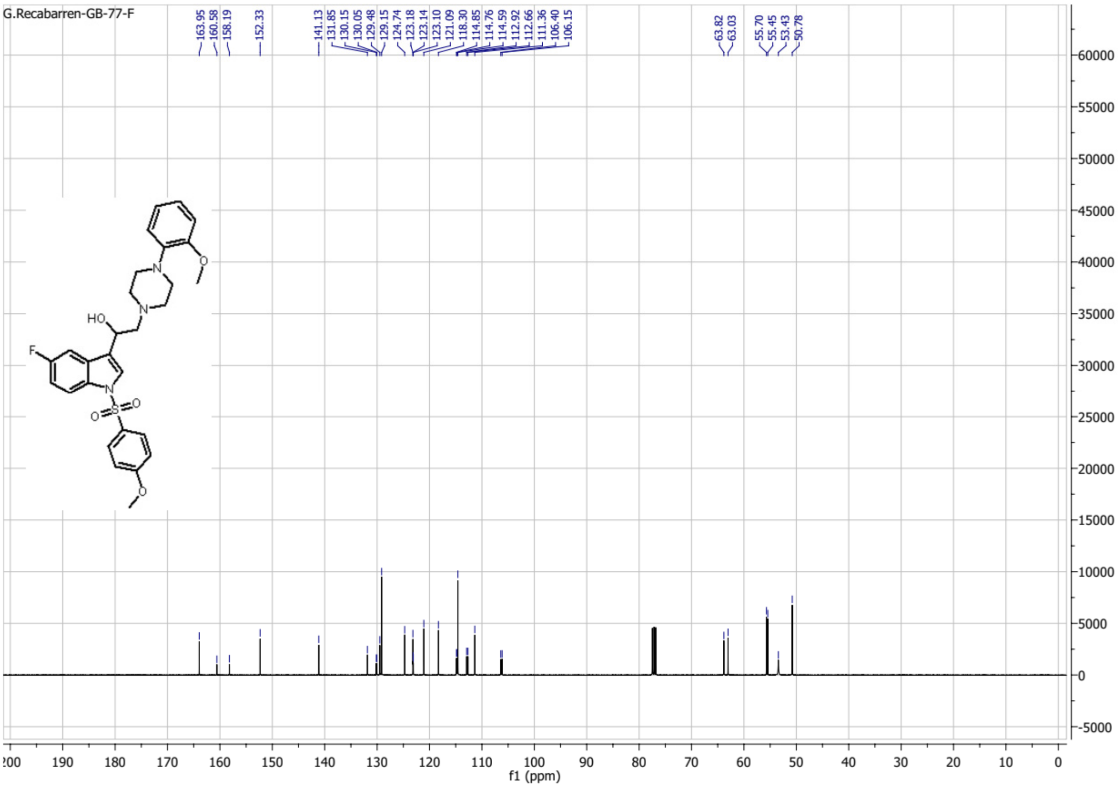

$^{19}$F-NMR (376.5 MHz, $CDCl_3$) for **4h**

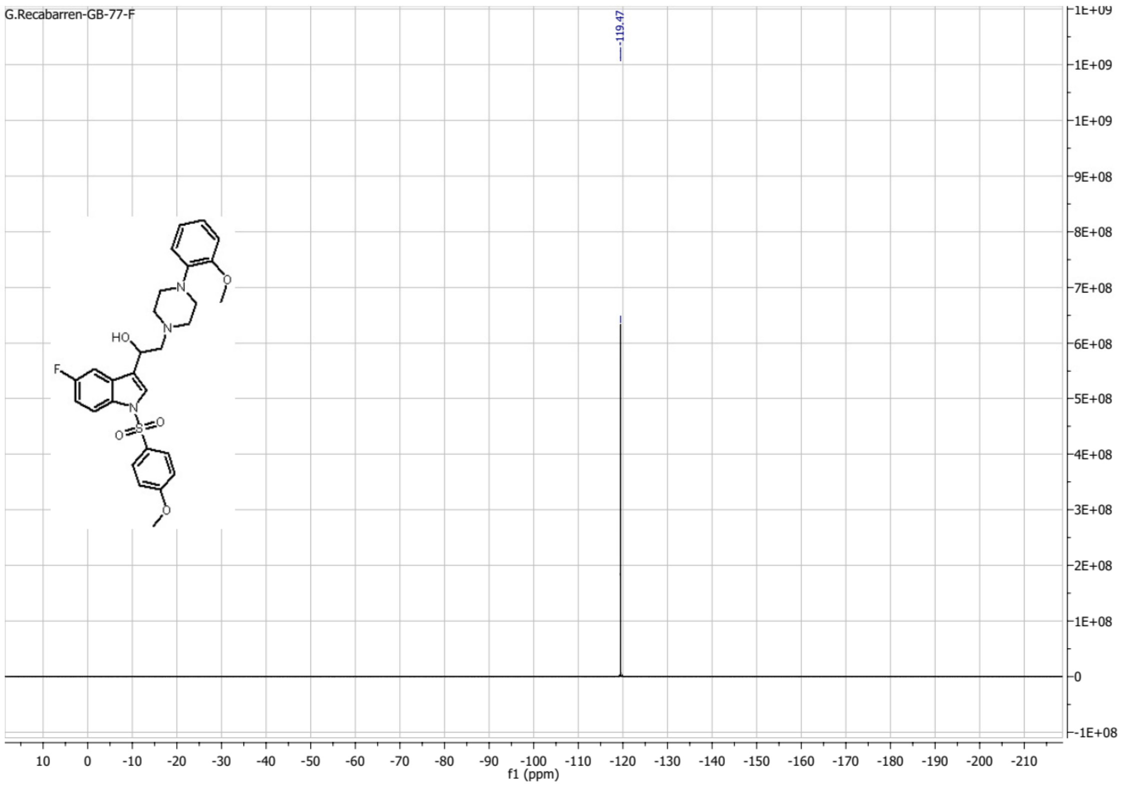

$^{1}H$-NMR (400.1 MHz, $CDCl_3$) for **4i**

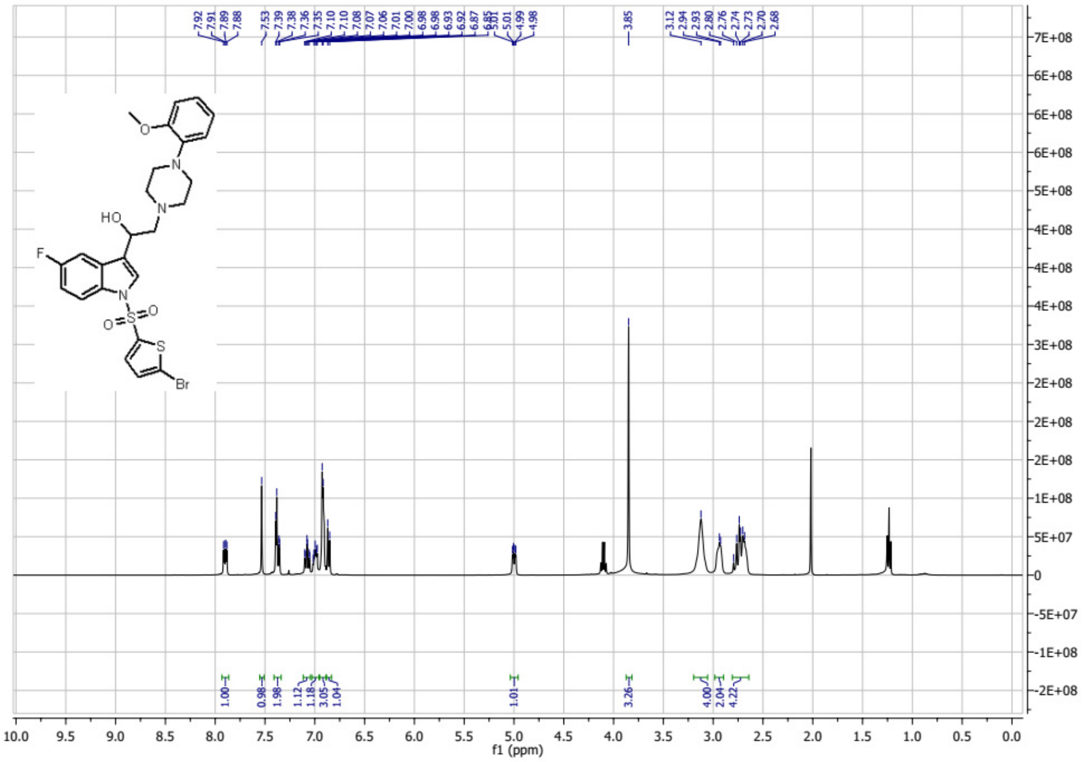

$^{13}C$-NMR (100.6 MHz, $CDCl_3$) for **4i**

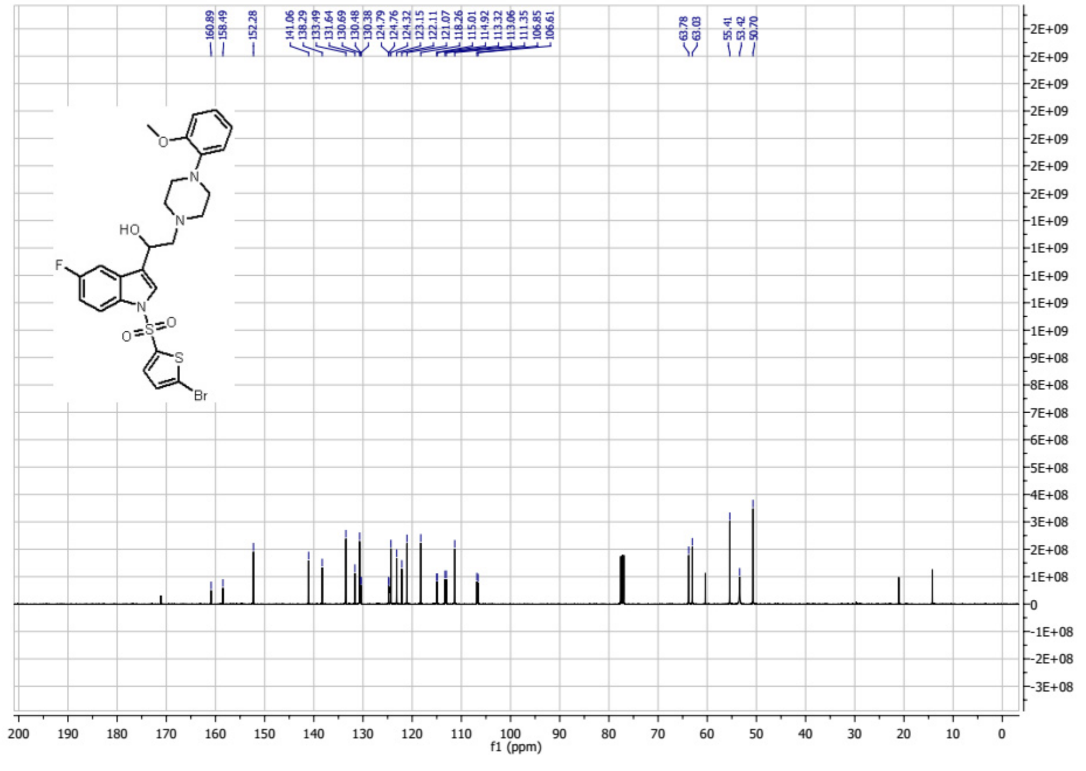

$^{1}$H-NMR (400.1 MHz, $CDCl_3$) for **4j**

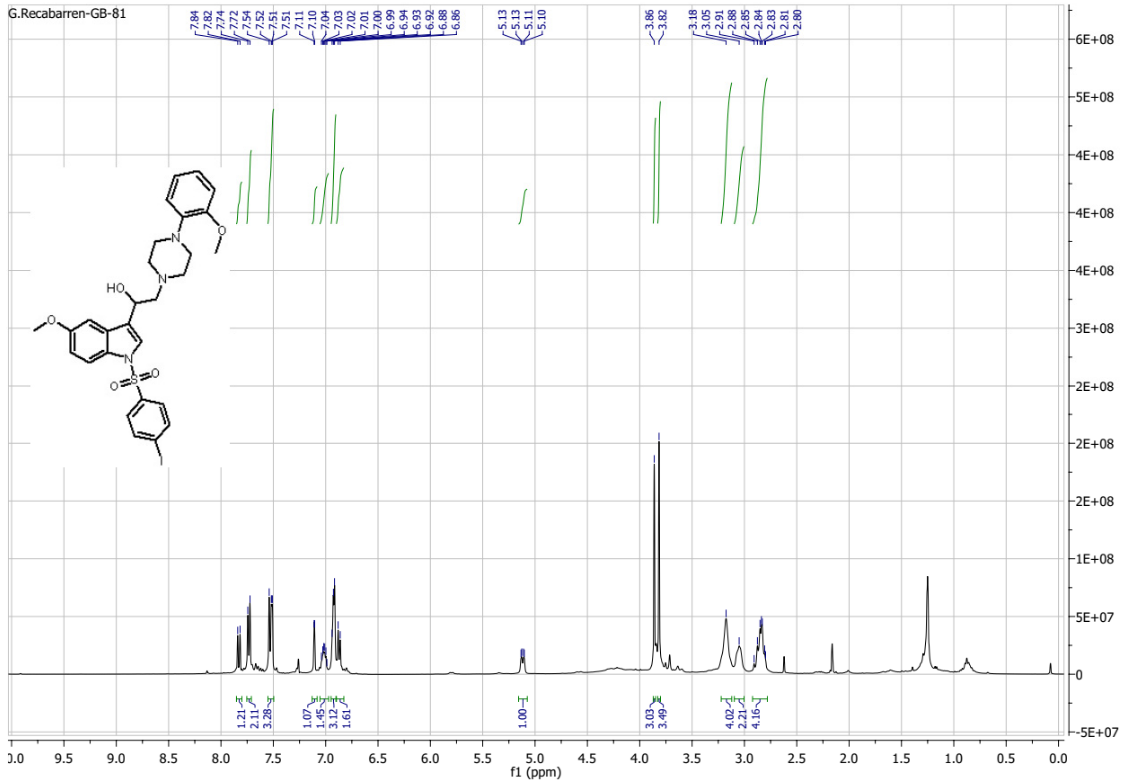

$^{13}$C-NMR (100.6 MHz, $CDCl_3$) for **4j**

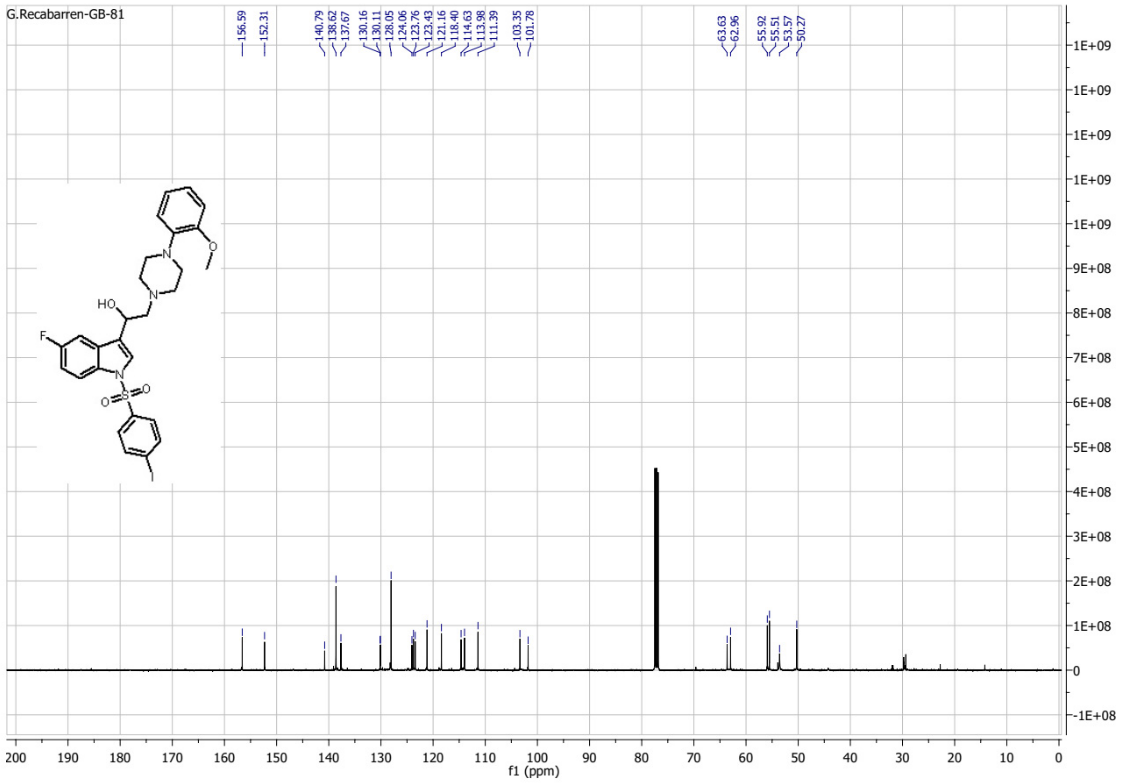

$^{1}$H-NMR (400.1 MHz, DMSO-d$_6$) for **4k**

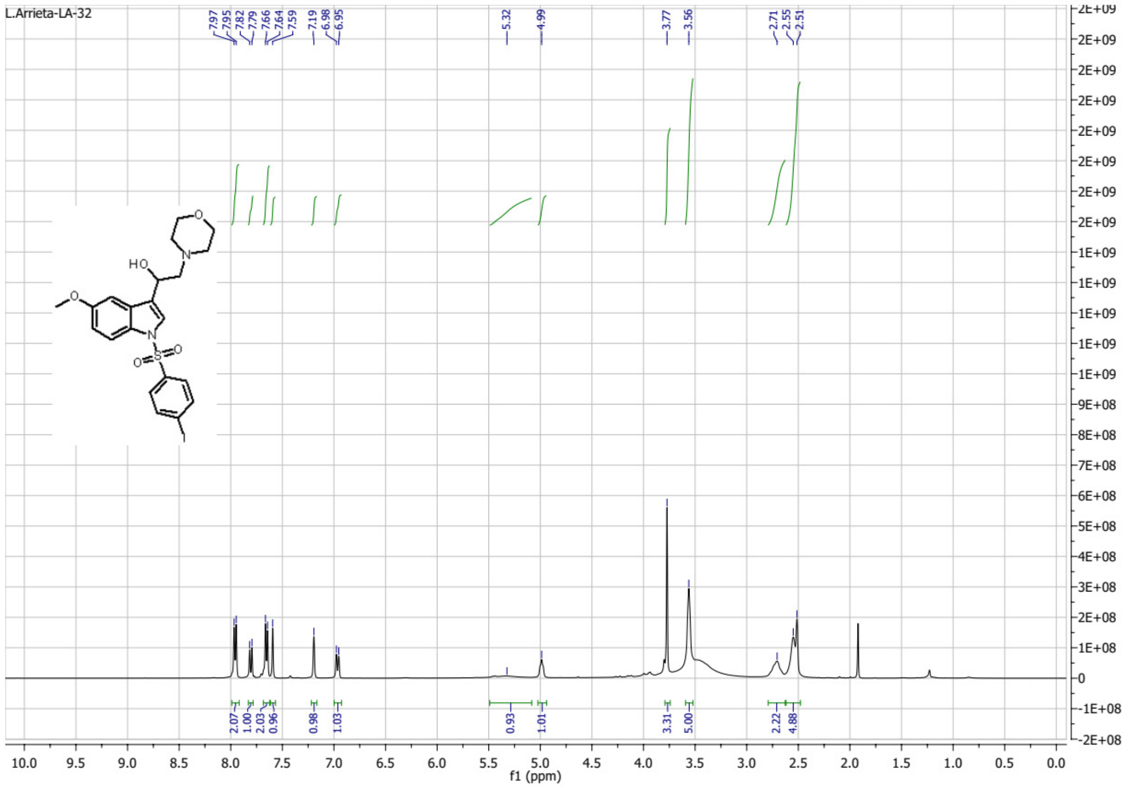


$^{13}$C-NMR (100.6 MHz, DMSO-d$_6$) for **4k**

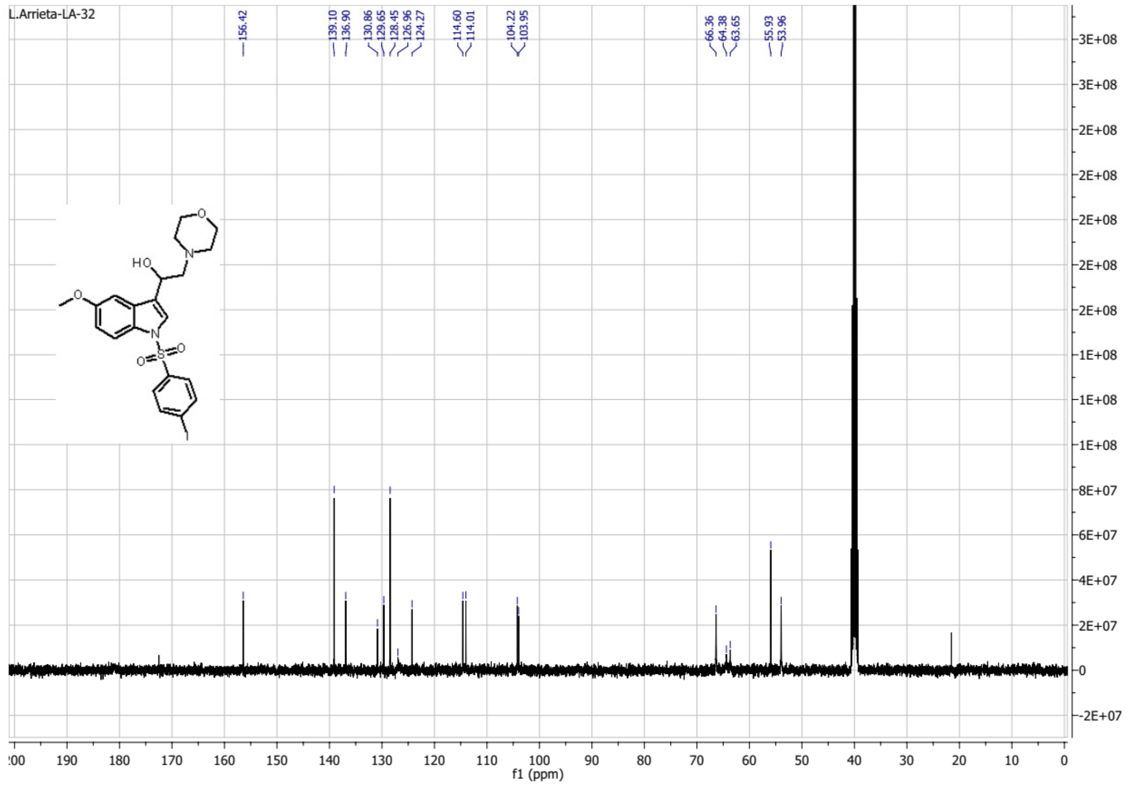

$^{1}$H-NMR (400.1 MHz, $CDCl_3$) for **4l**

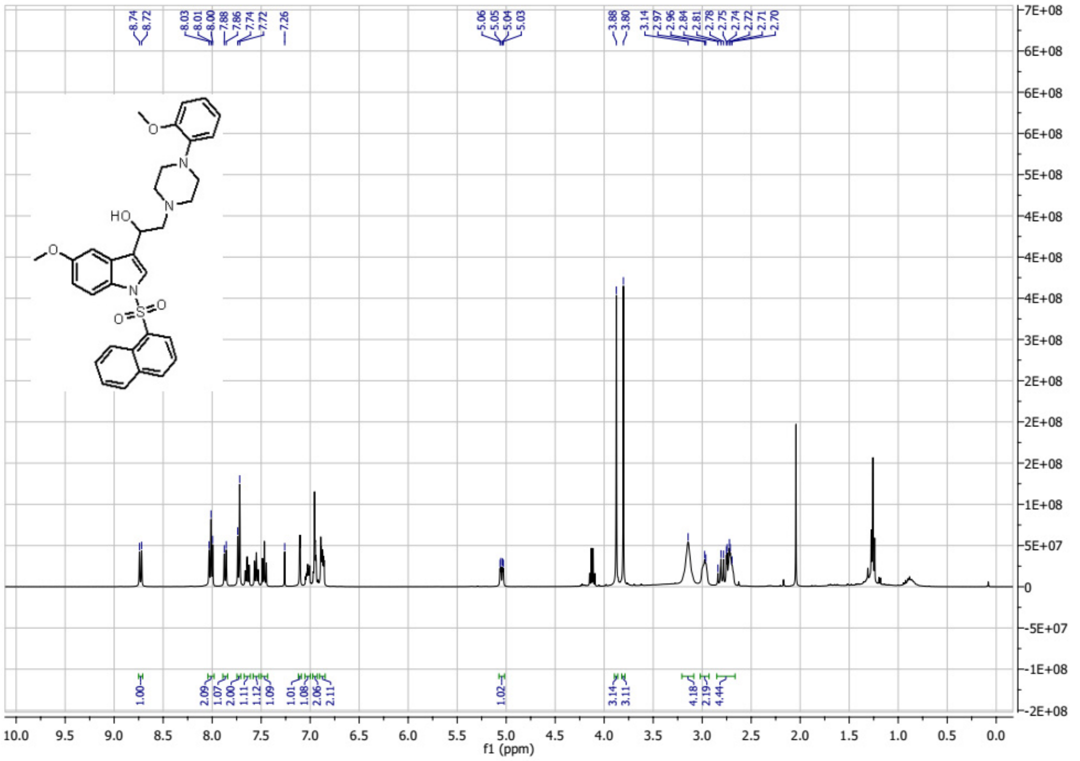


$^{13}$C-NMR (100.6 MHz, $CDCl_3$) for **4l**

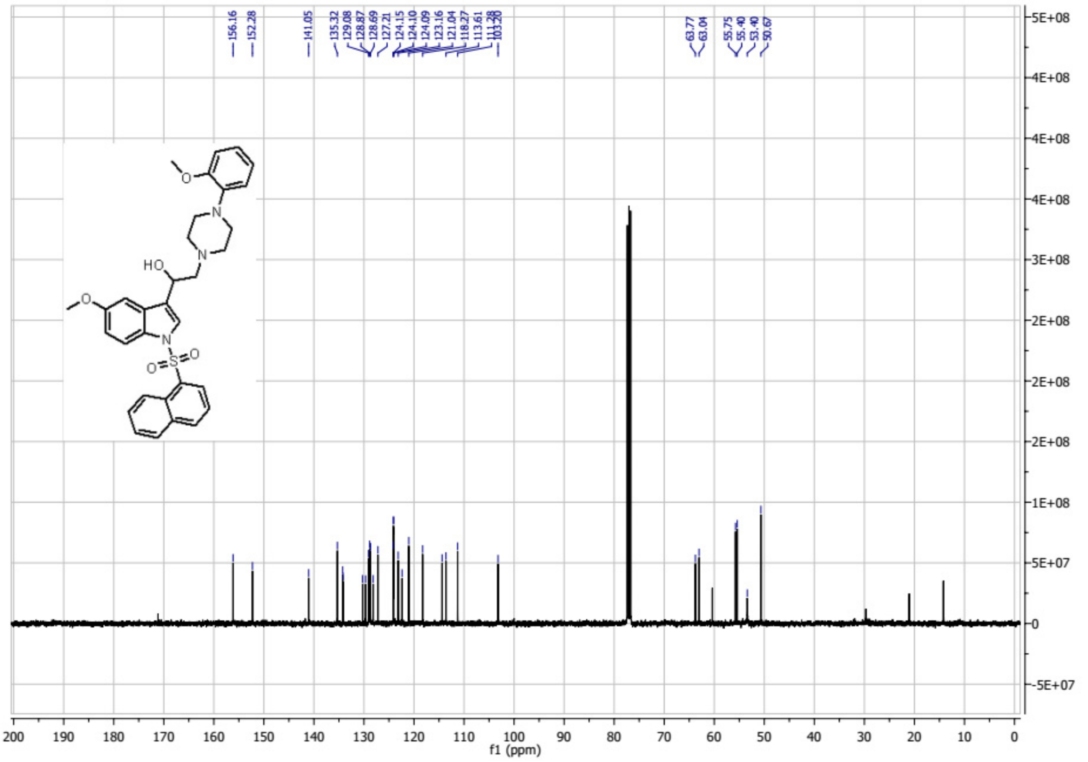

$^{1}$H-NMR (400.1 MHz, DMSO-d6) for **4m**

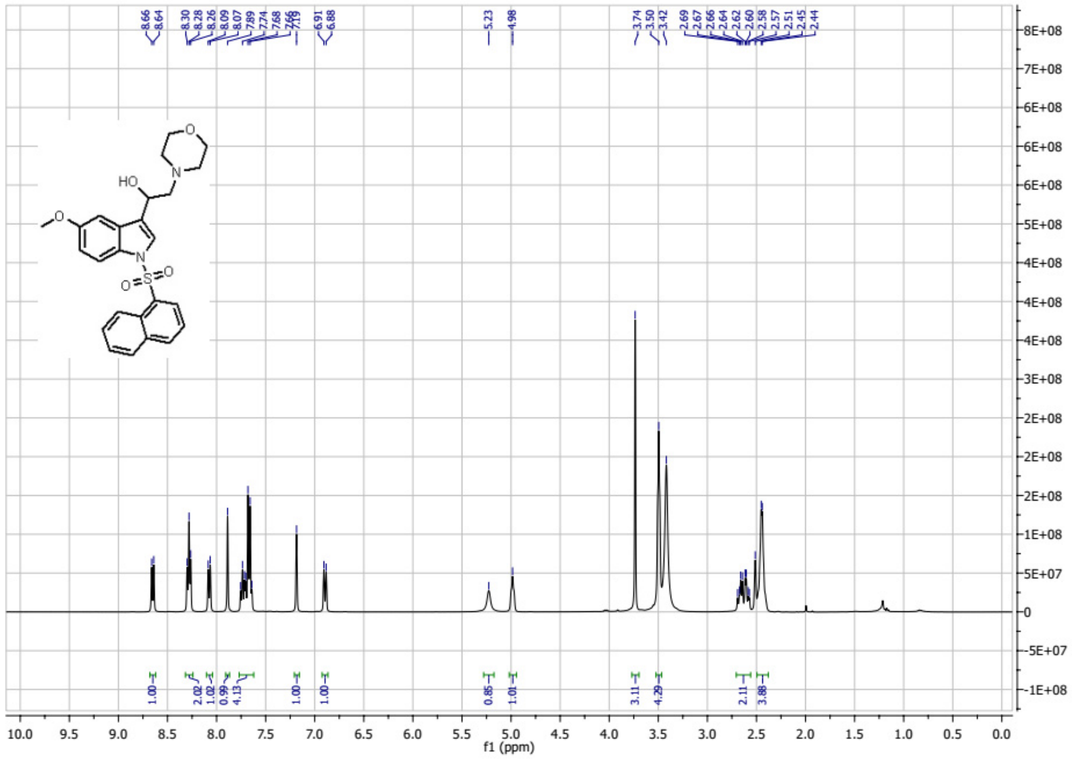

$^{13}$C-NMR (100.6 MHz, DMSO-d6) for **4m**

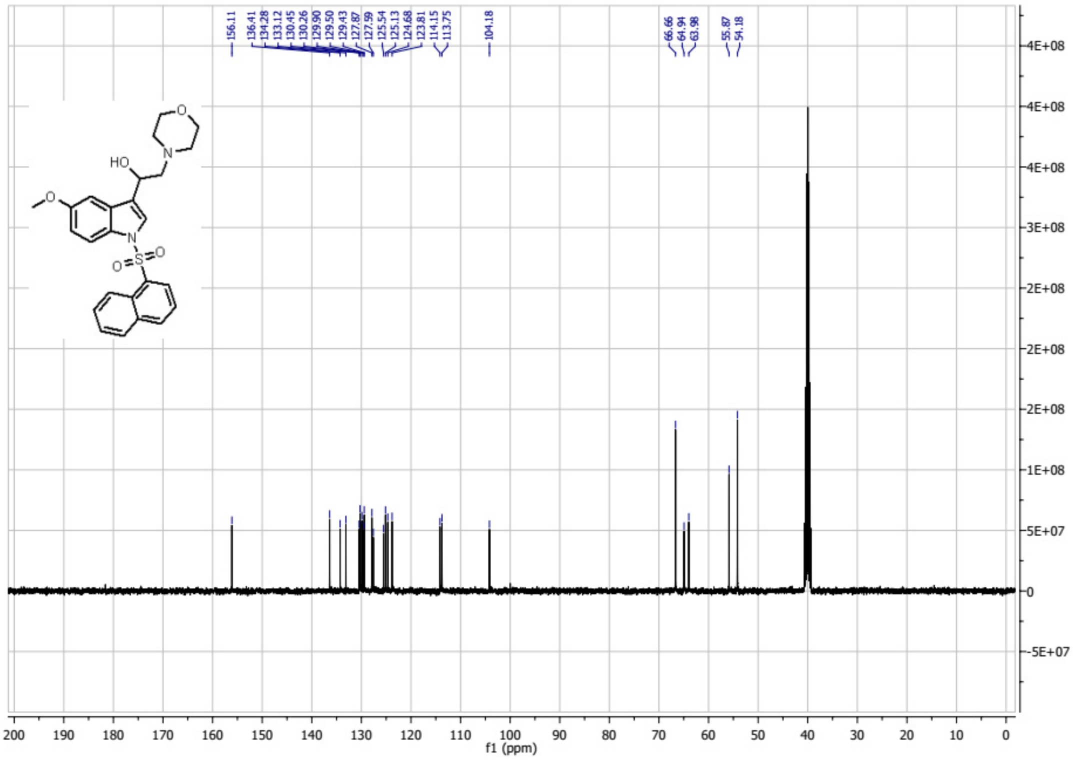

$^{1}$H-NMR (400.1 MHz, $CDCl_3$) for **4n**

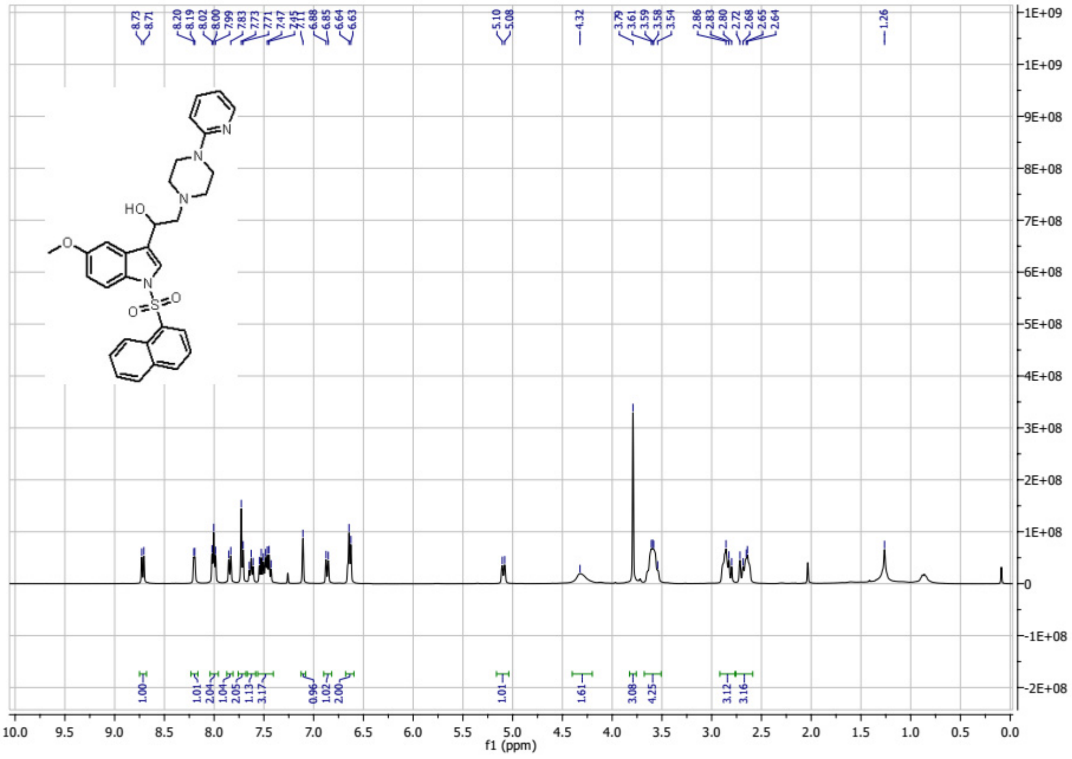

$^{13}$C-NMR (100.6 MHz, $CDCl_3$) for **4n**

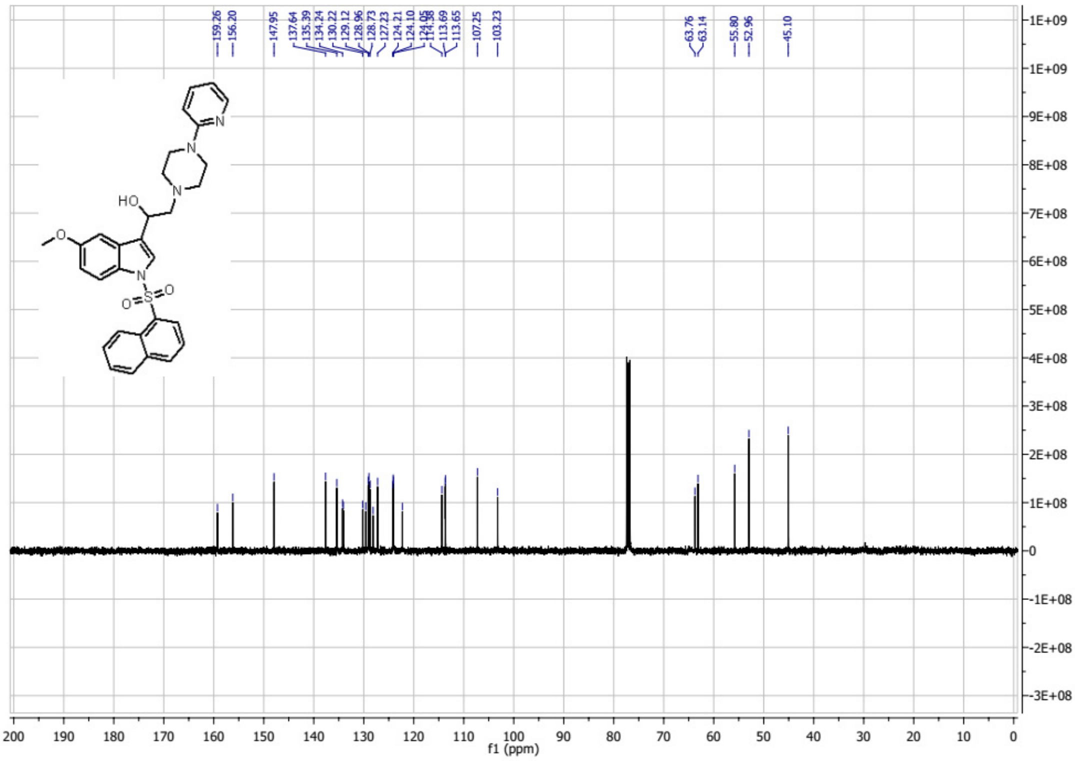

$^{1}$H-NMR (400.1 MHz, $CDCl_3$) for **4o**

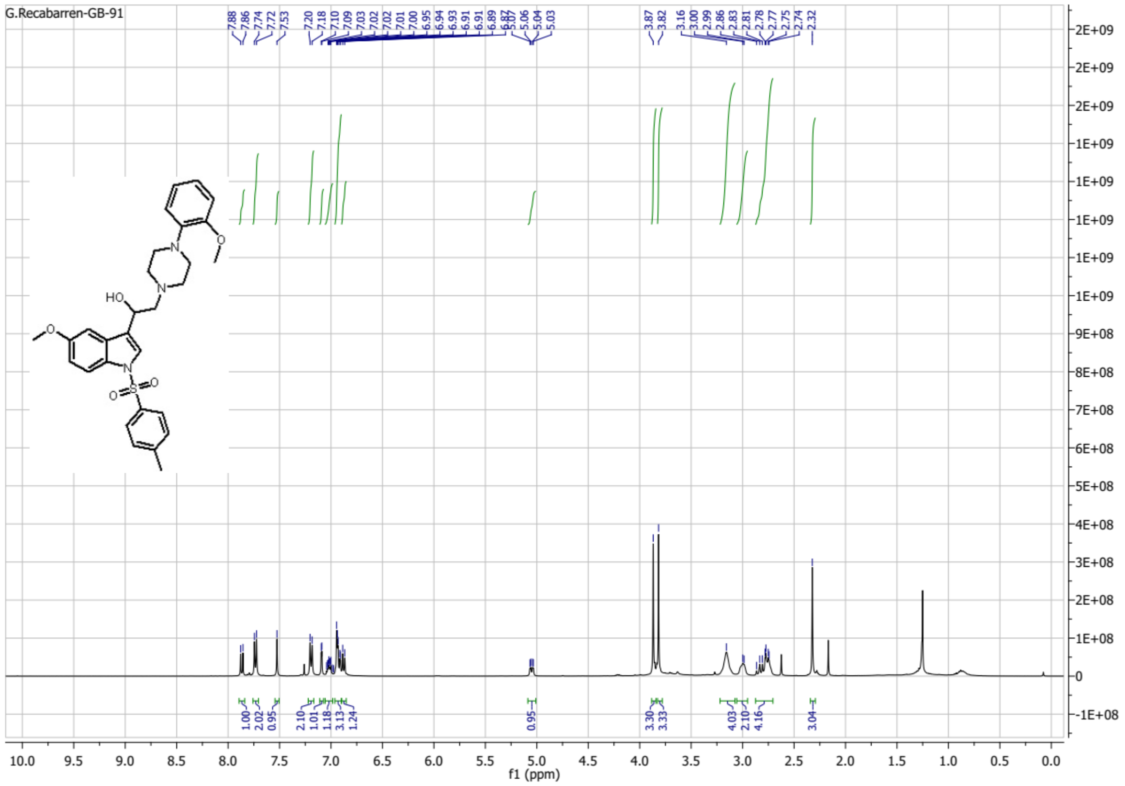

$^{13}$C-NMR (100.6 MHz, $CDCl_3$) for **4o**

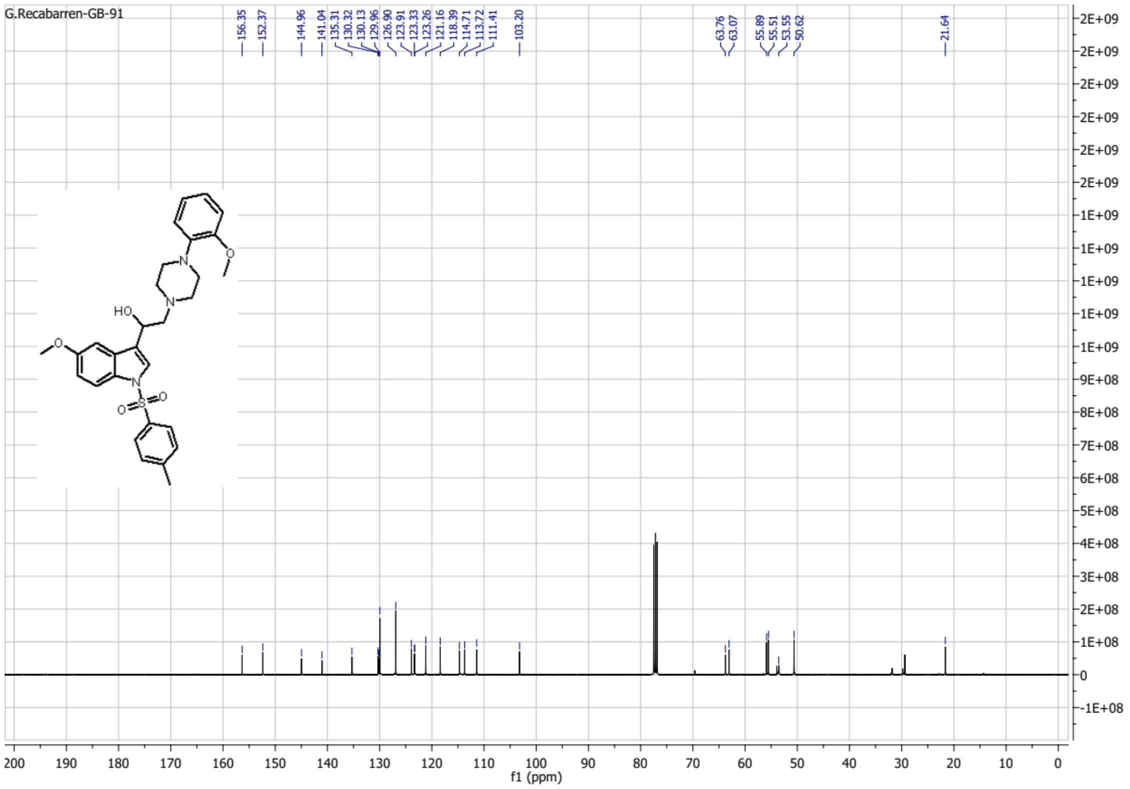

$^{1}$H-NMR (400.1 MHz, $CDCl_3$) for **4p**

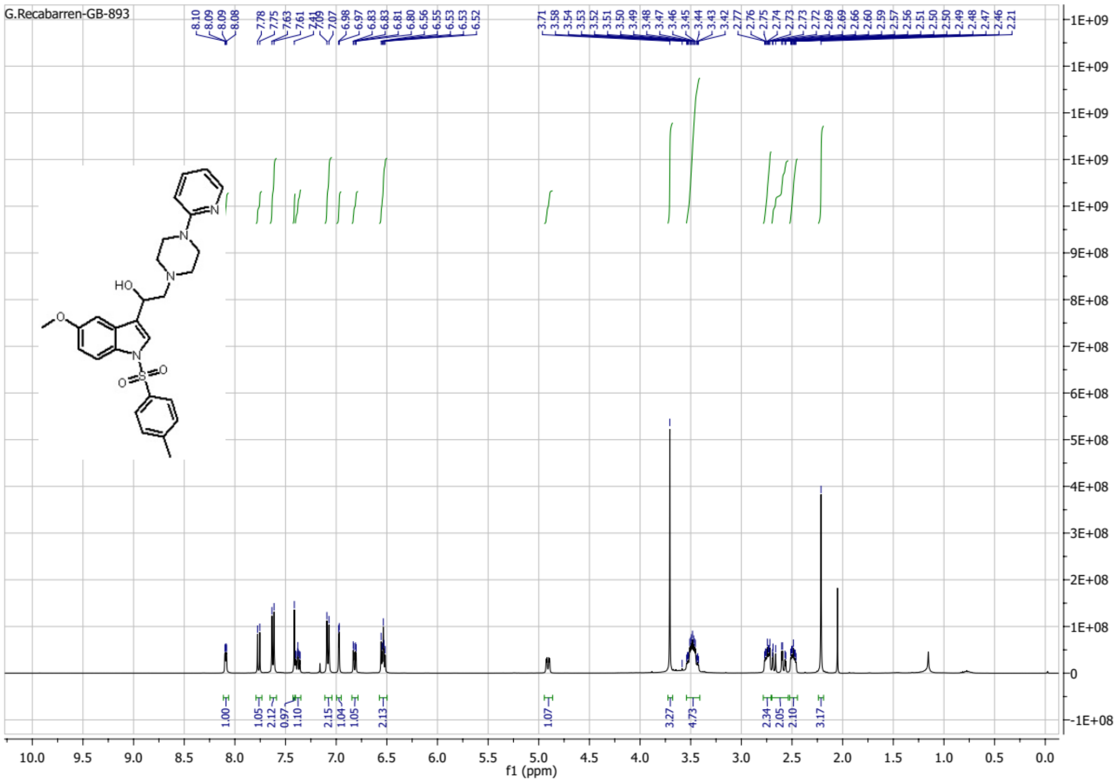

$^{13}$C-NMR (100.6 MHz, $CDCl_3$) for **4p**

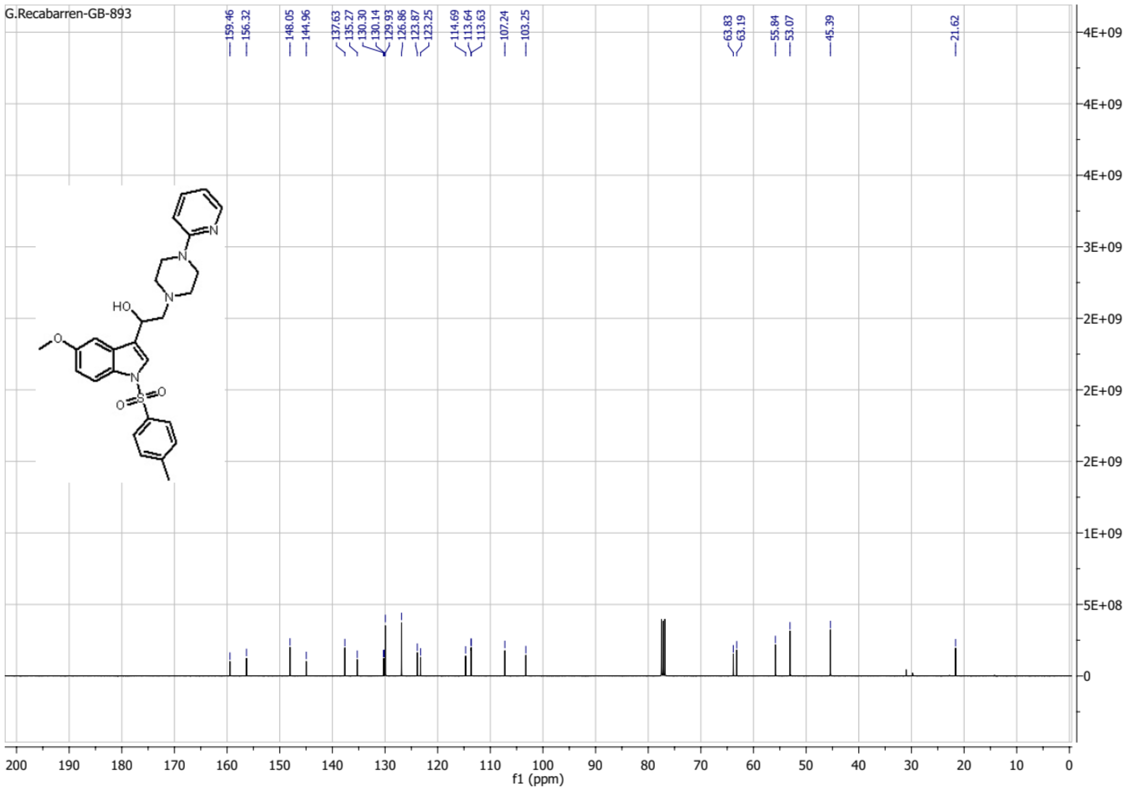

$^{1}$H-NMR (400.1 MHz, $CDCl_3$) for **4q**

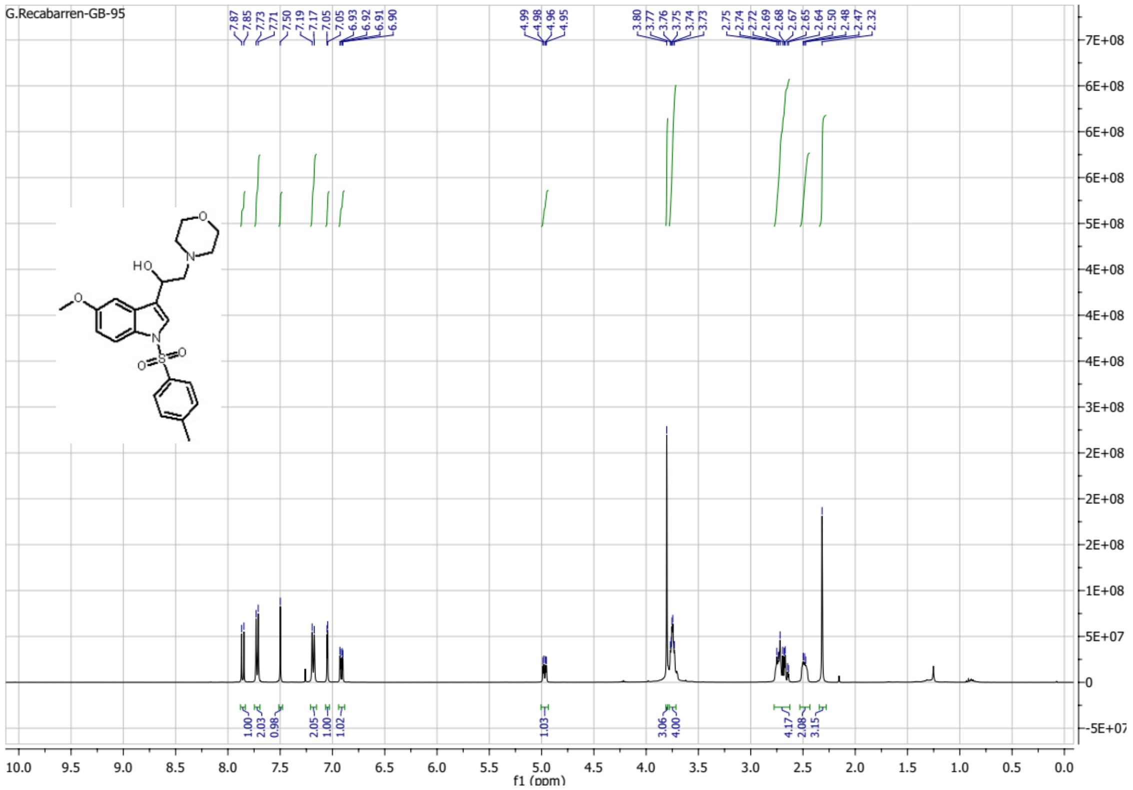


$^{13}$C-NMR (100.6 MHz, $CDCl_3$) for **4q**

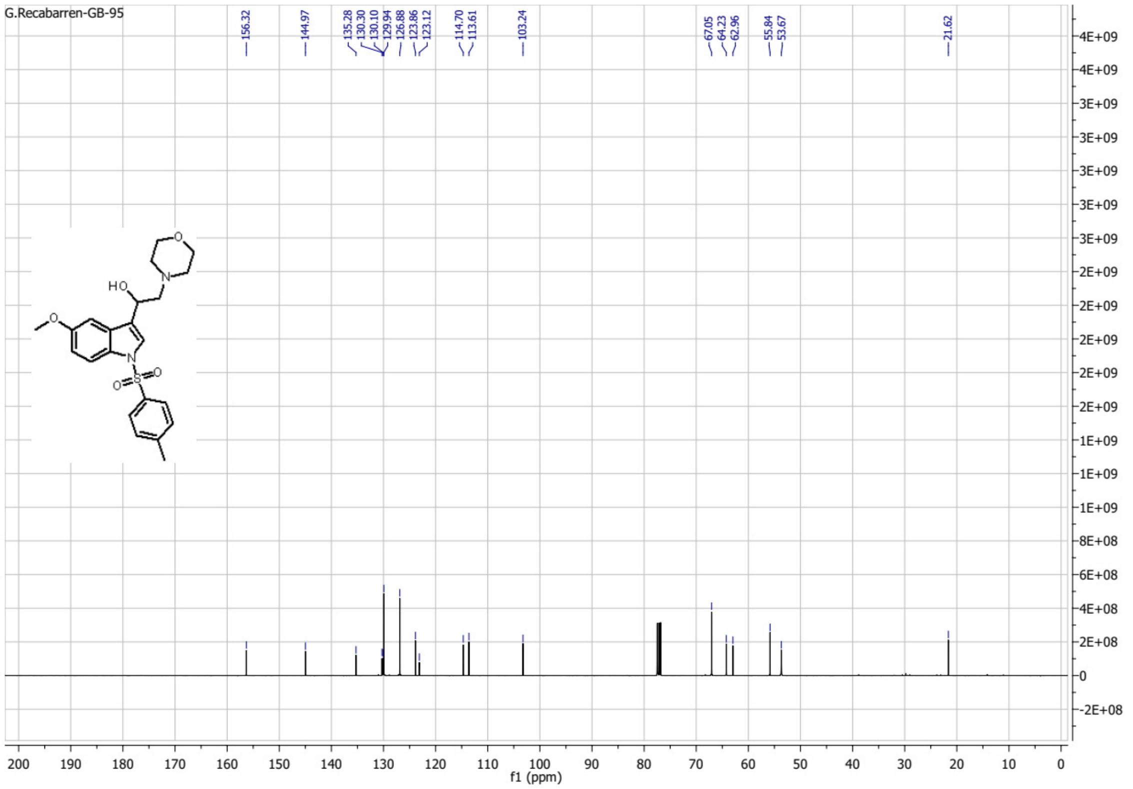

$^1$H-NMR (400.1 MHz, $CDCl_3$) for **4r**

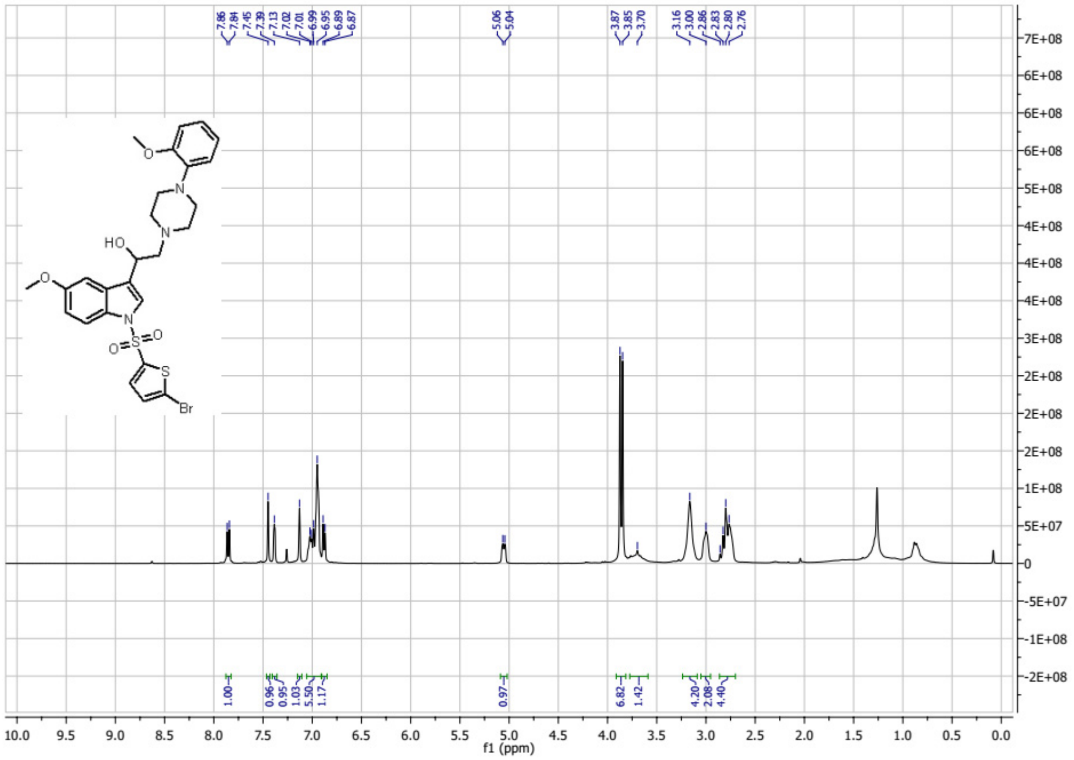


$^{13}$C-NMR (100.6 MHz, $CDCl_3$) for **4r**

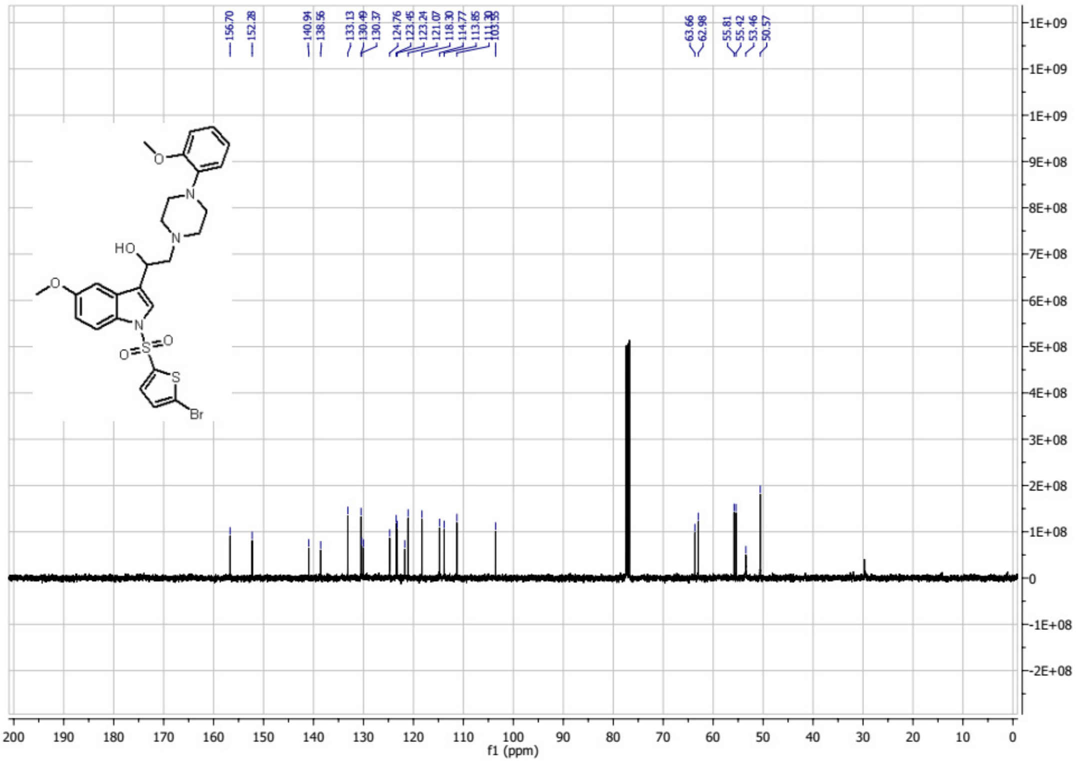

$^{1}H$-NMR (400.1 MHz, $CDCl_3$) for **4s**

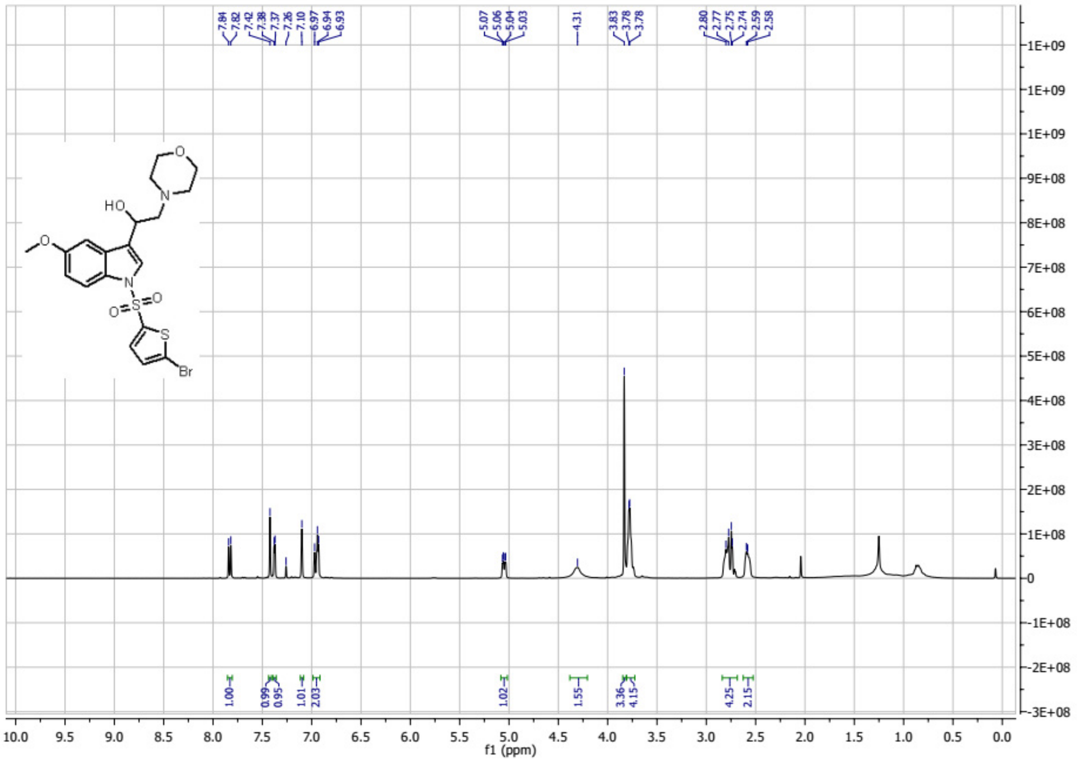

$^{13}C$-NMR (100.6 MHz, $CDCl_3$) for **4s**

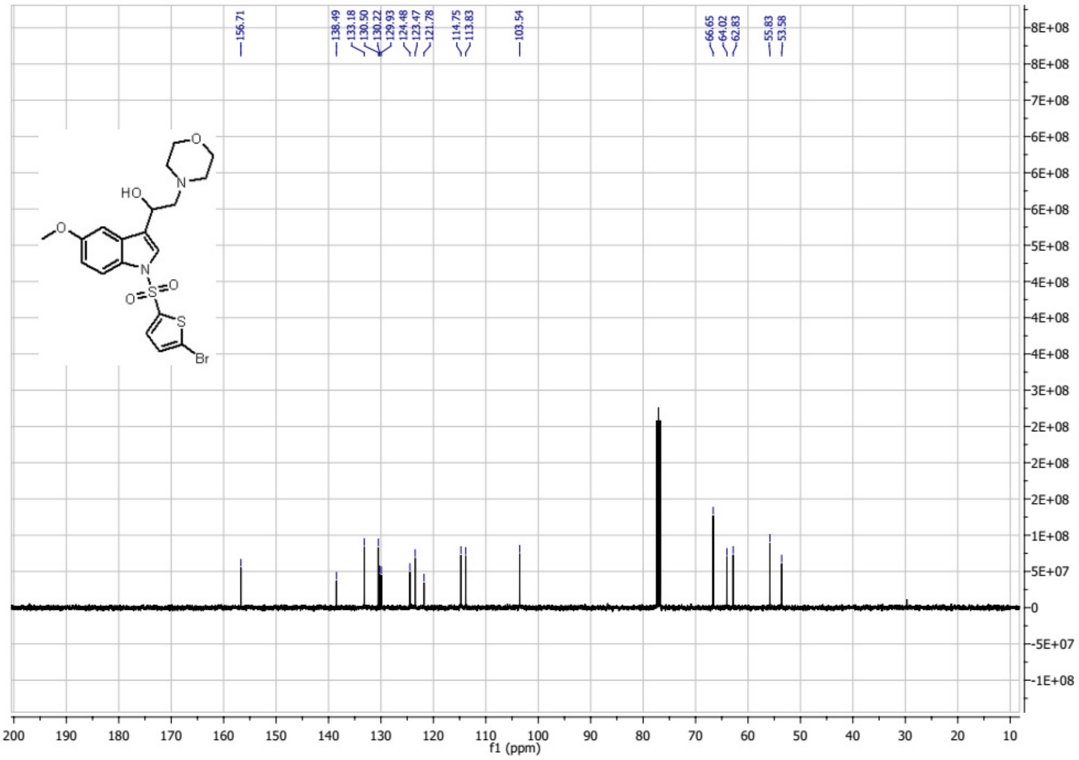

$^{1}$H-NMR (400.1 MHz, DMSO-d6) for **4t**

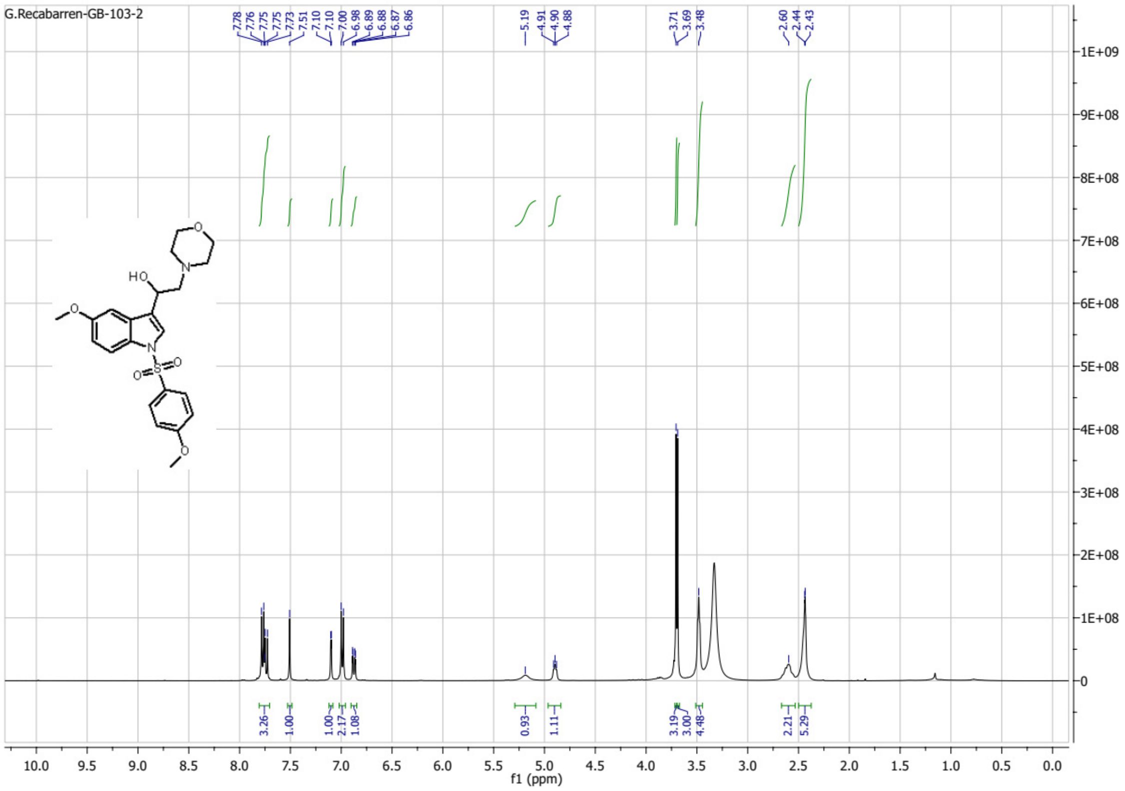


$^{13}$C-NMR (100.6 MHz, DMSO-d6) for **4t**

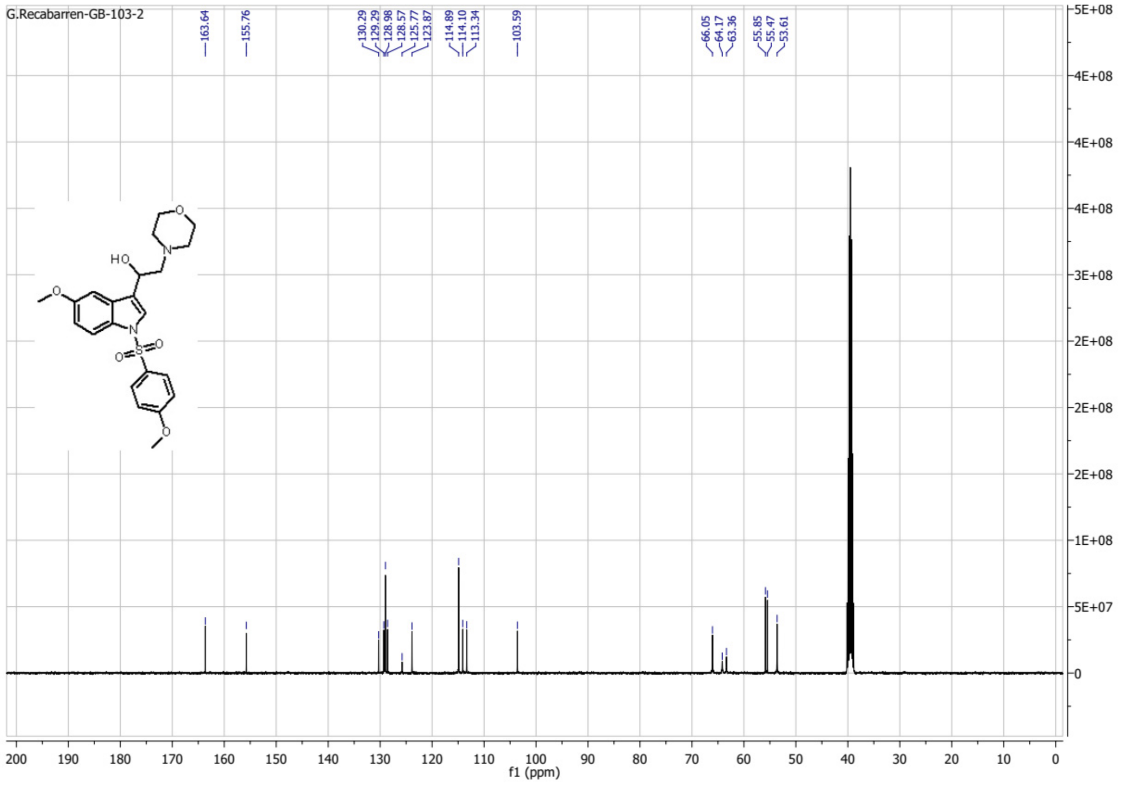

$^{1}$H-NMR (400.1 MHz, $CDCl_3$) for **4u**

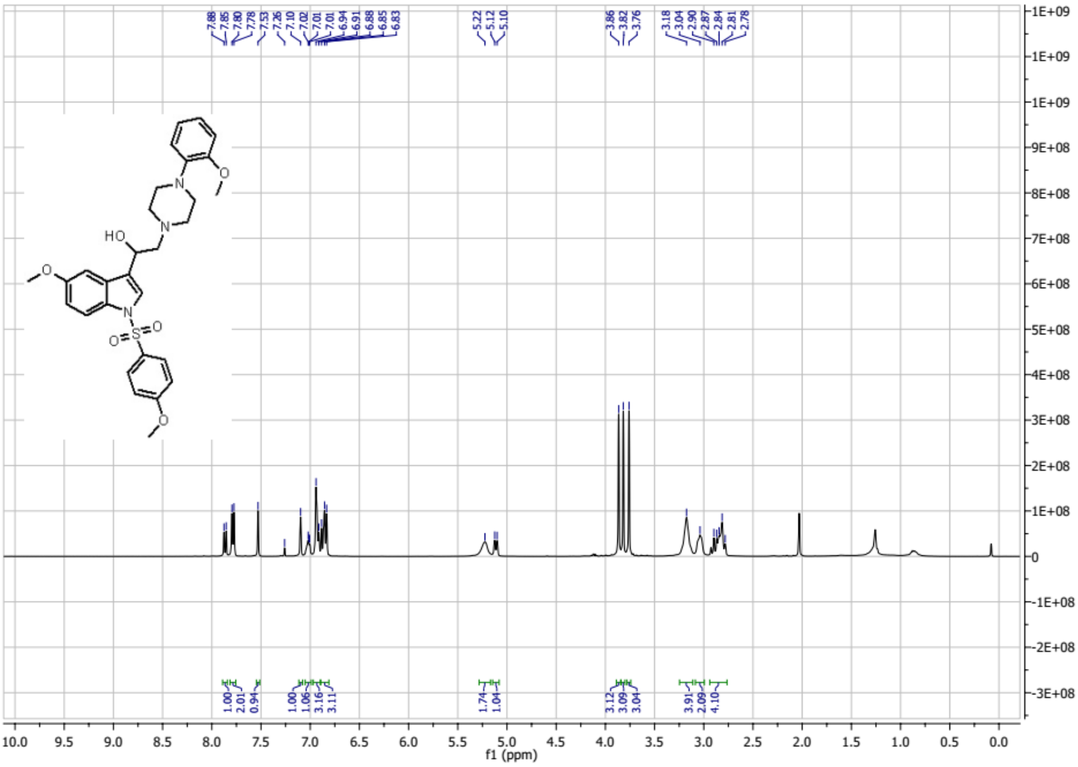

$^{13}$C-NMR (100.6 MHz, $CDCl_3$) for **4u**

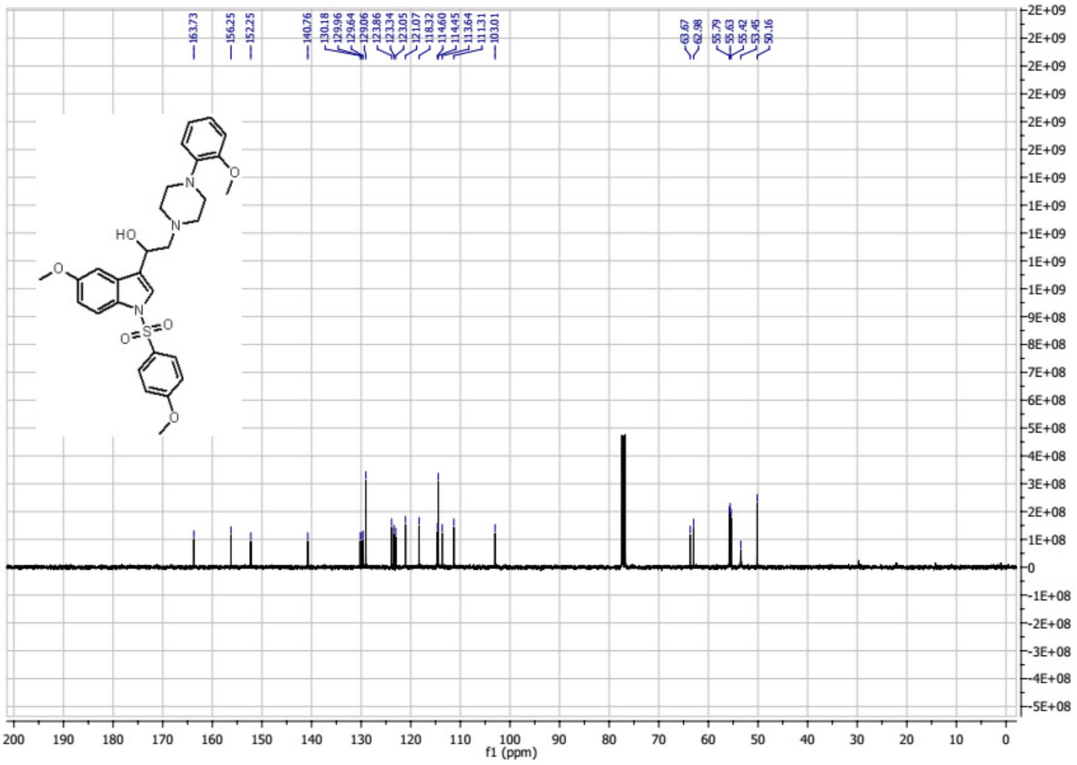

$^{1}$H-NMR (400.1 MHz, $CDCl_3$) for **4v**

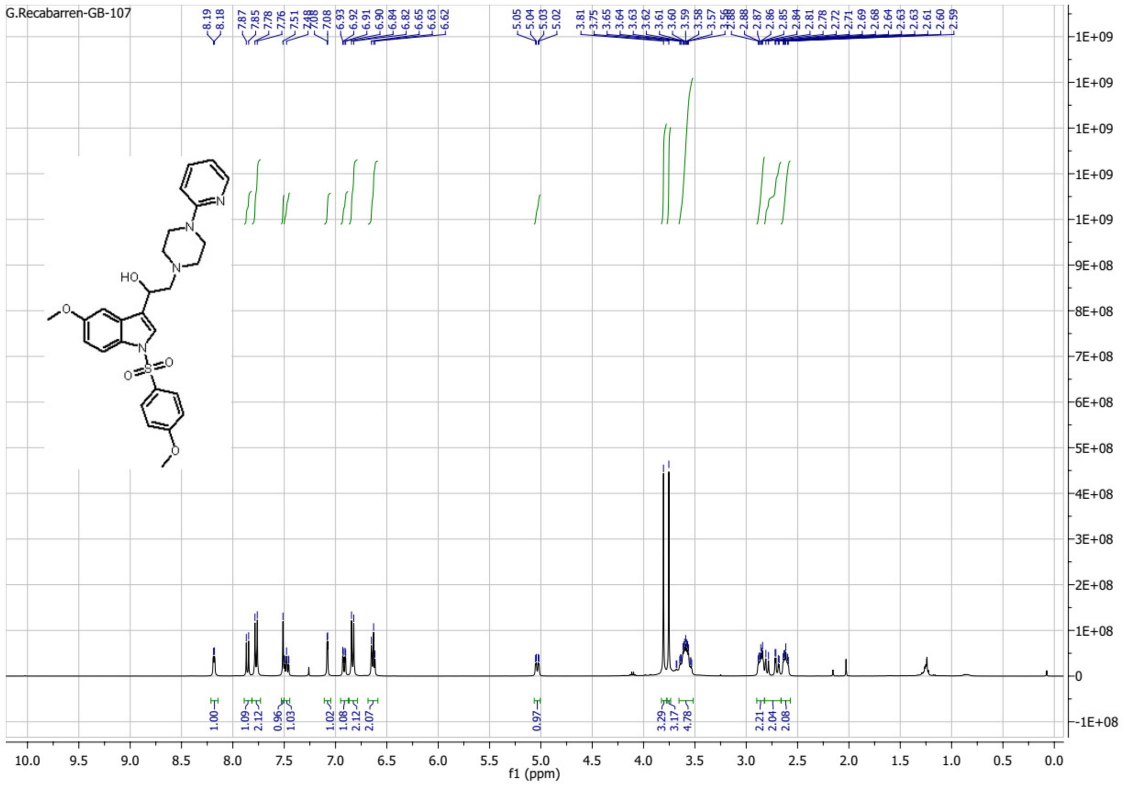

$^{13}$C-NMR (100.6 MHz, $CDCl_3$) for **4v**

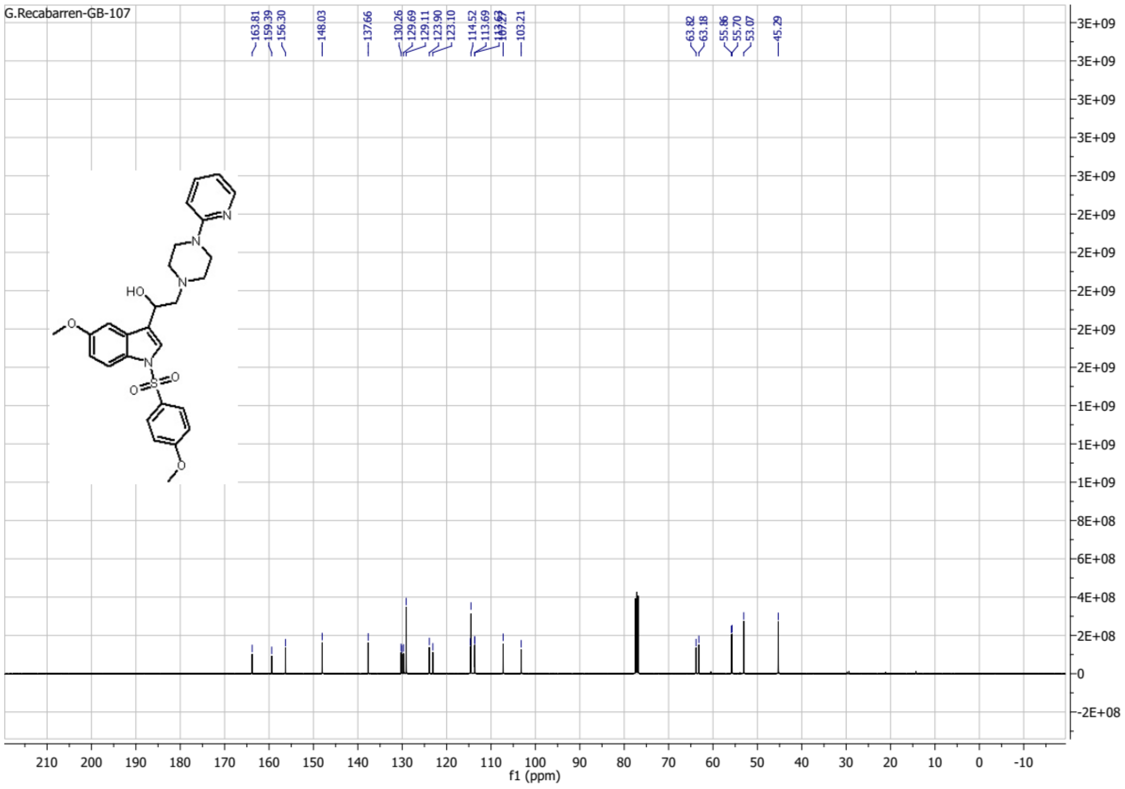

### 3. High resolution mass spectra of final compounds.

HRMS for **4a**

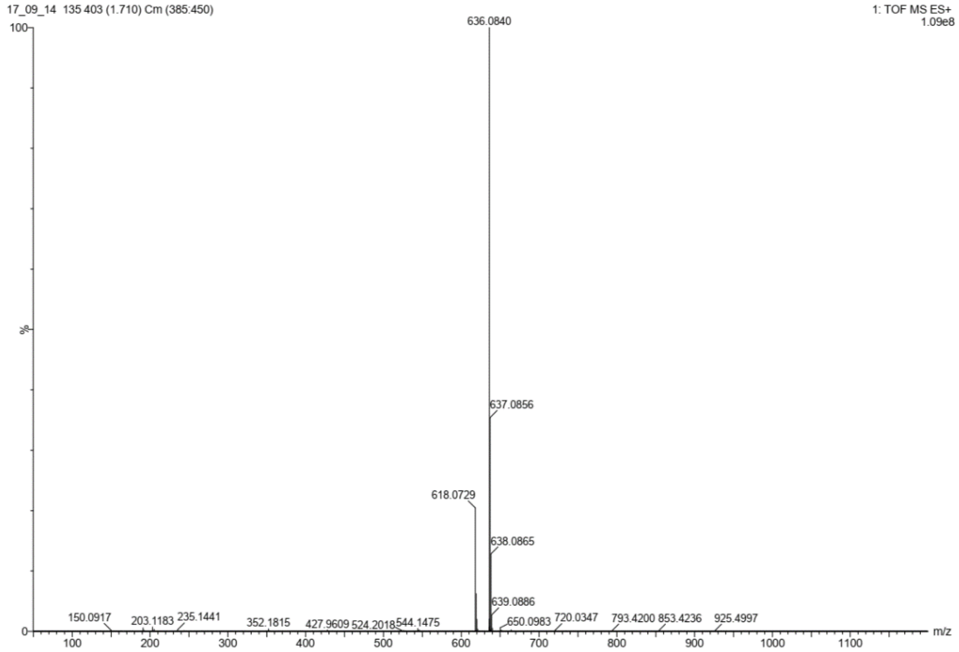


HRMS for **4b**

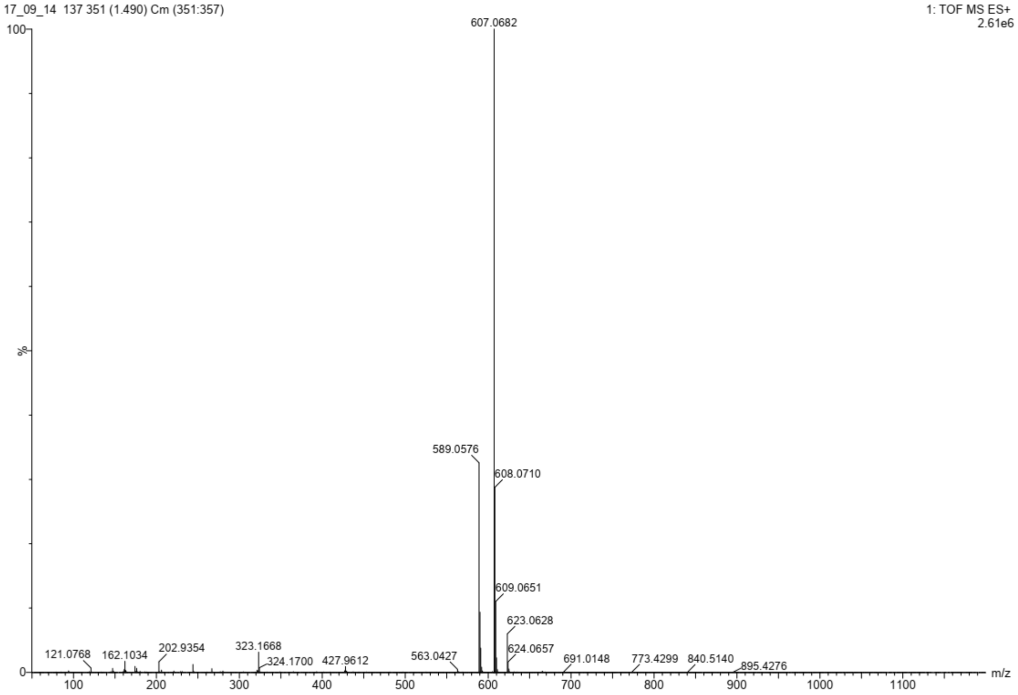

HRMS for **4c**

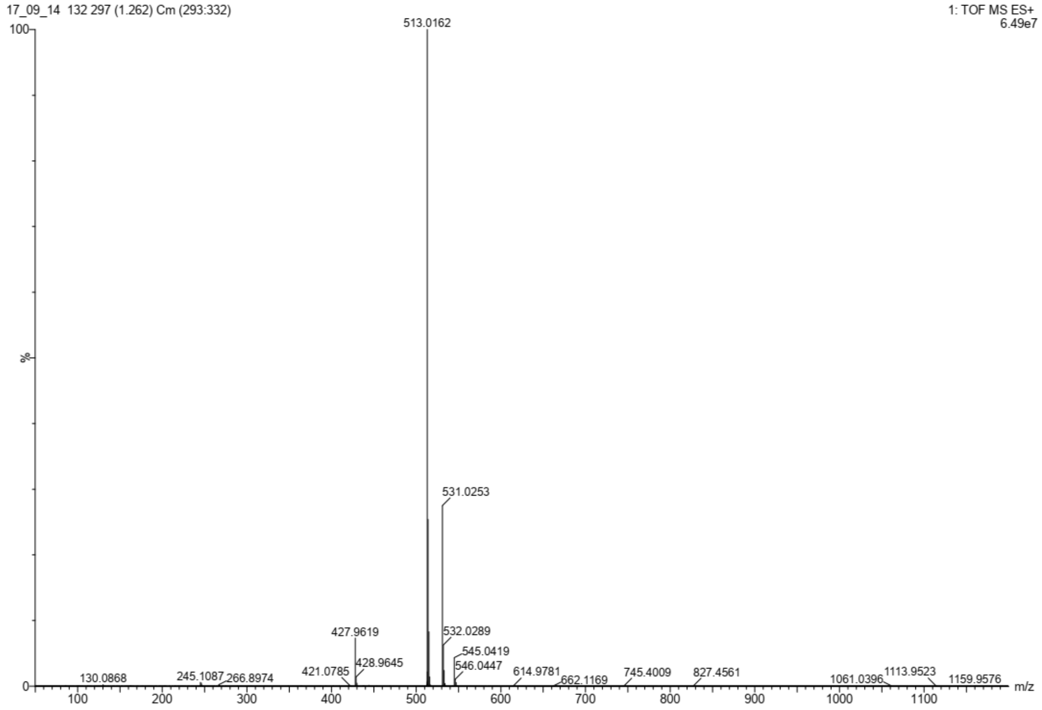


HRMS for **4d**

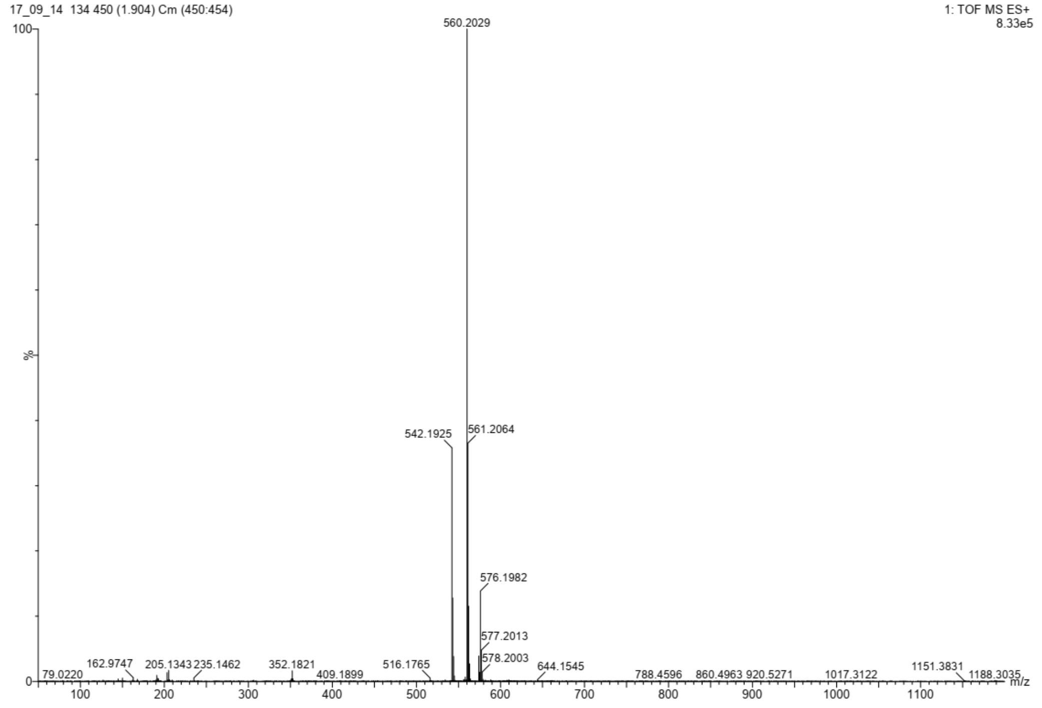

HRMS for **4e**

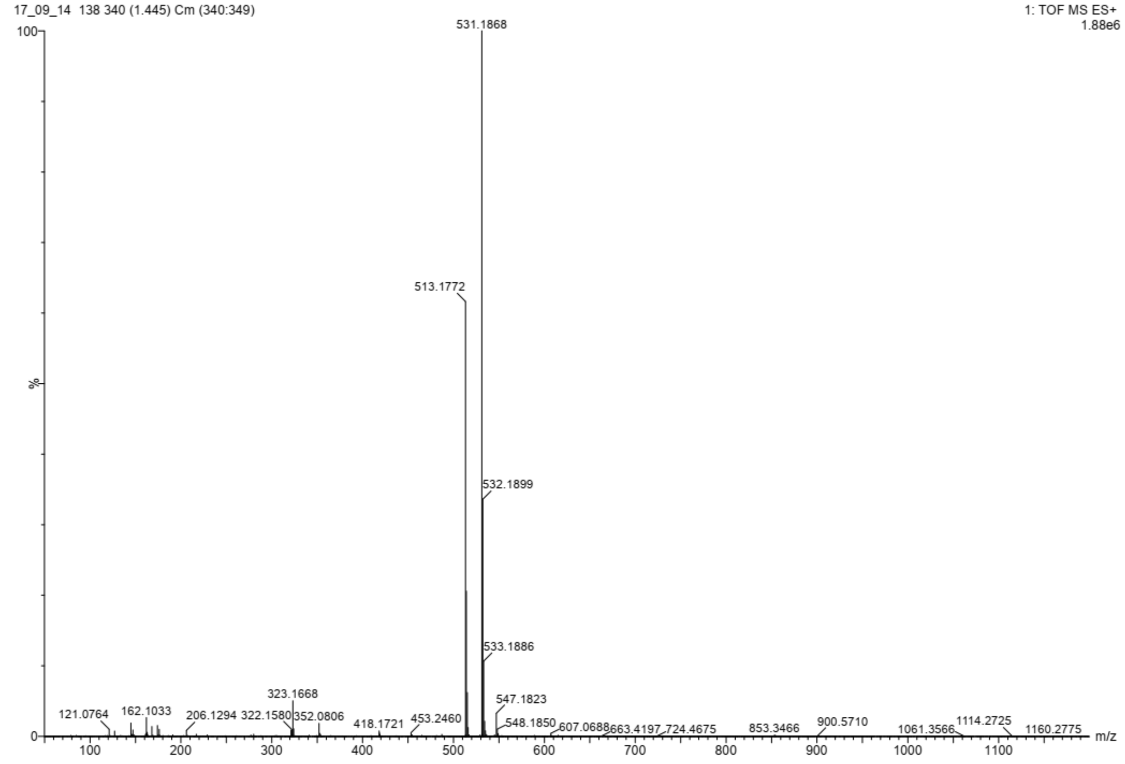


HRMS for **4f**

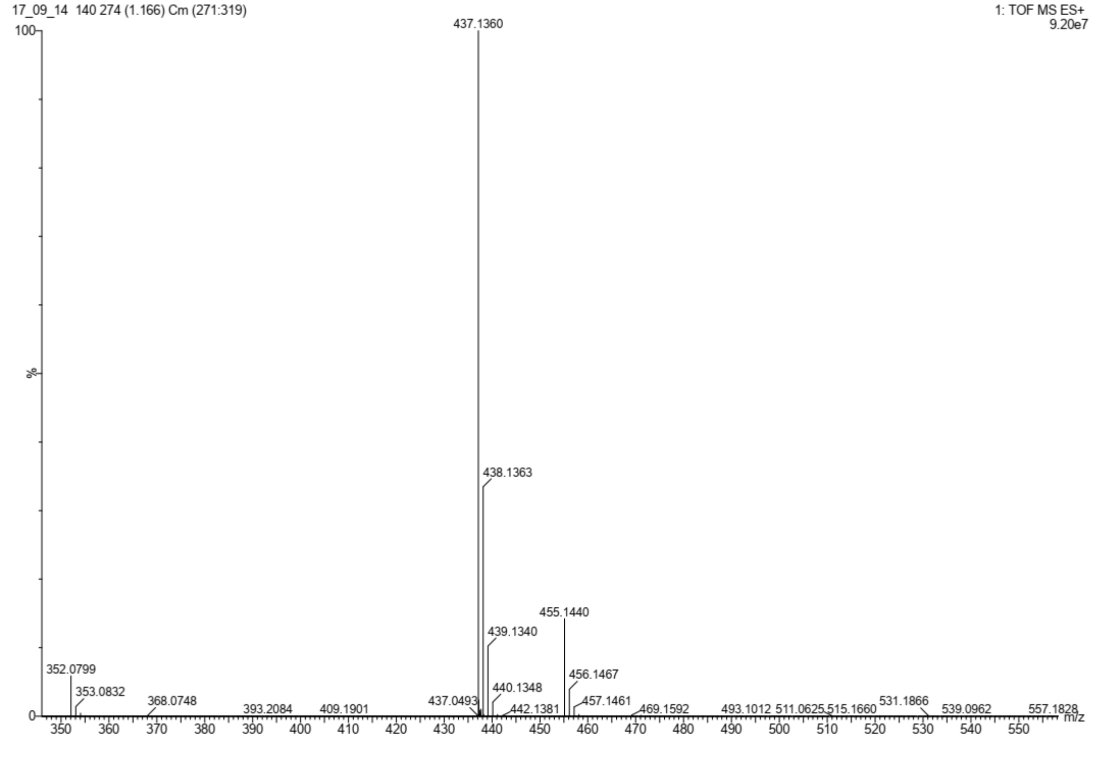

HRMS for **4g**

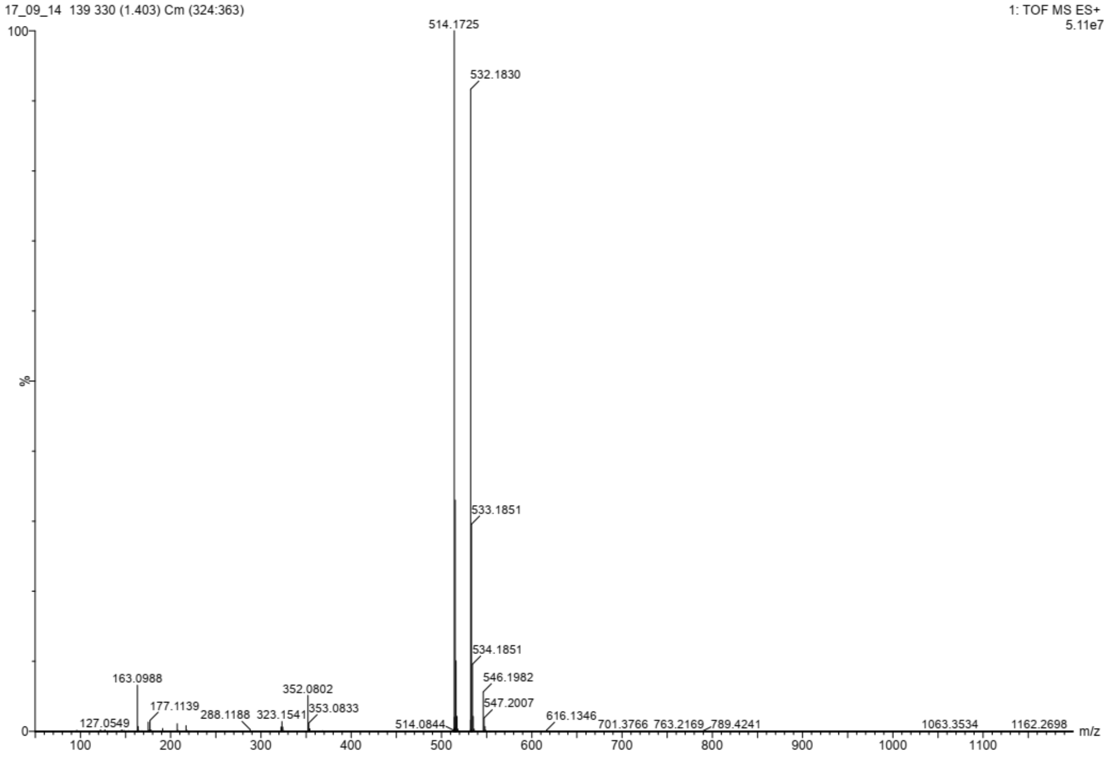


HRMS for **4h**

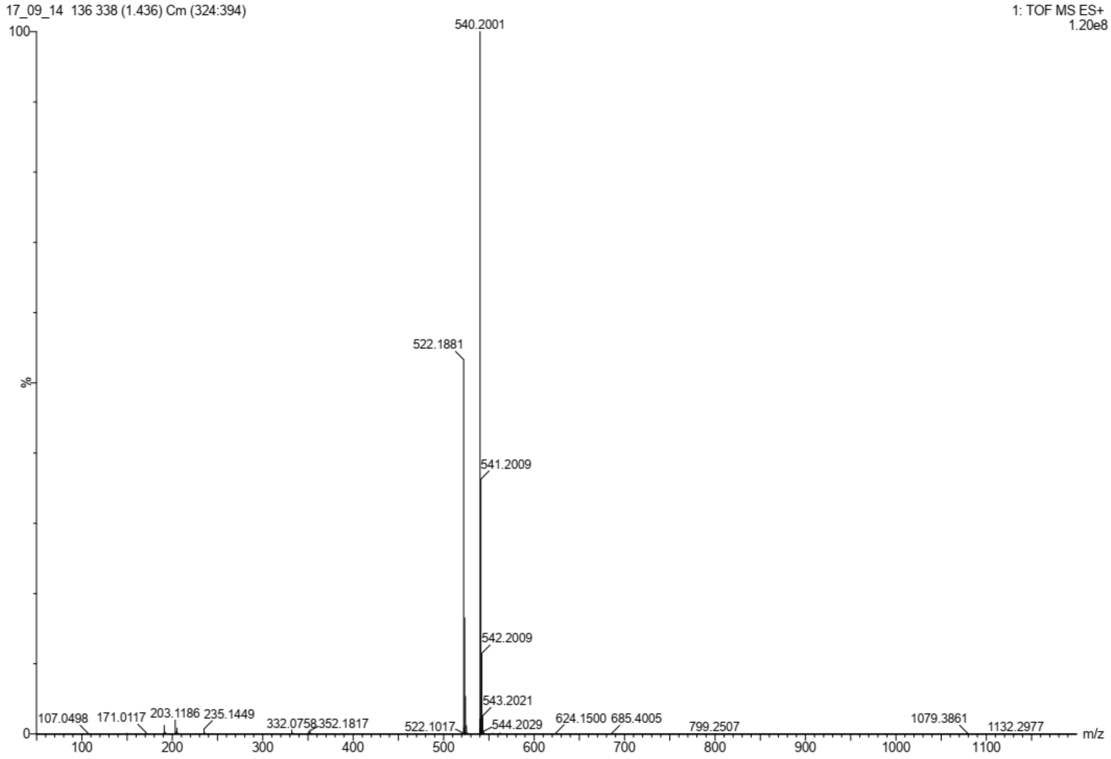

HRMS for **4i**

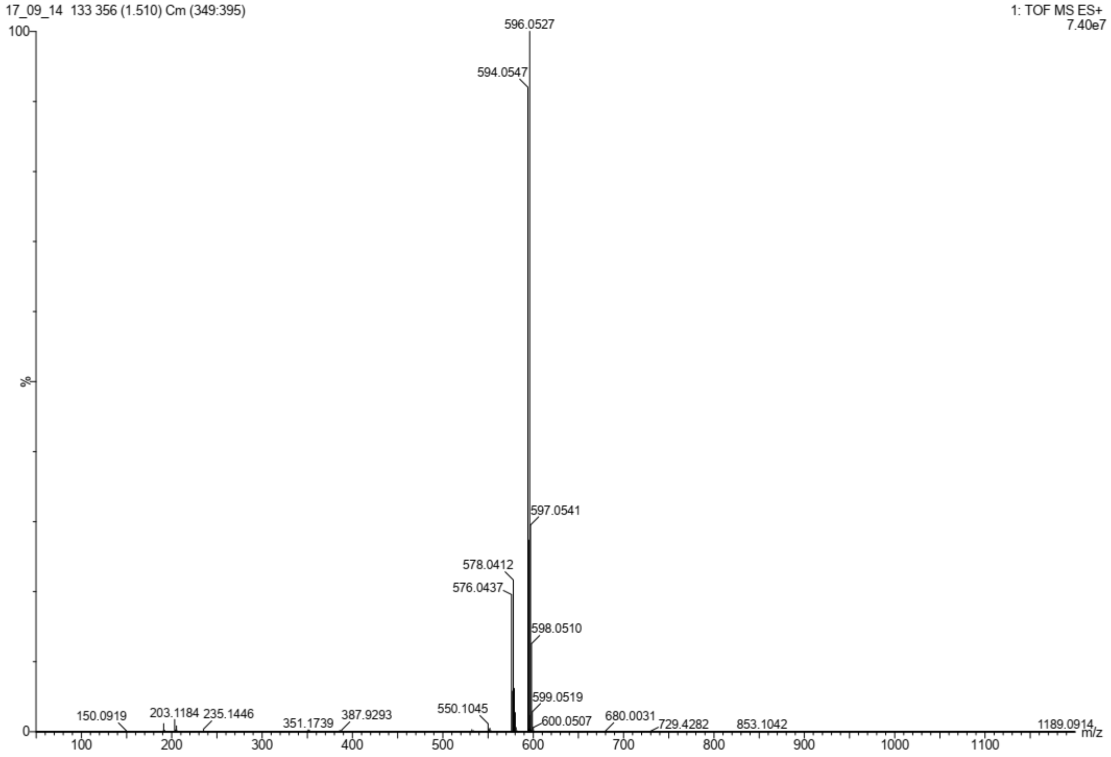


HRMS for **4j**

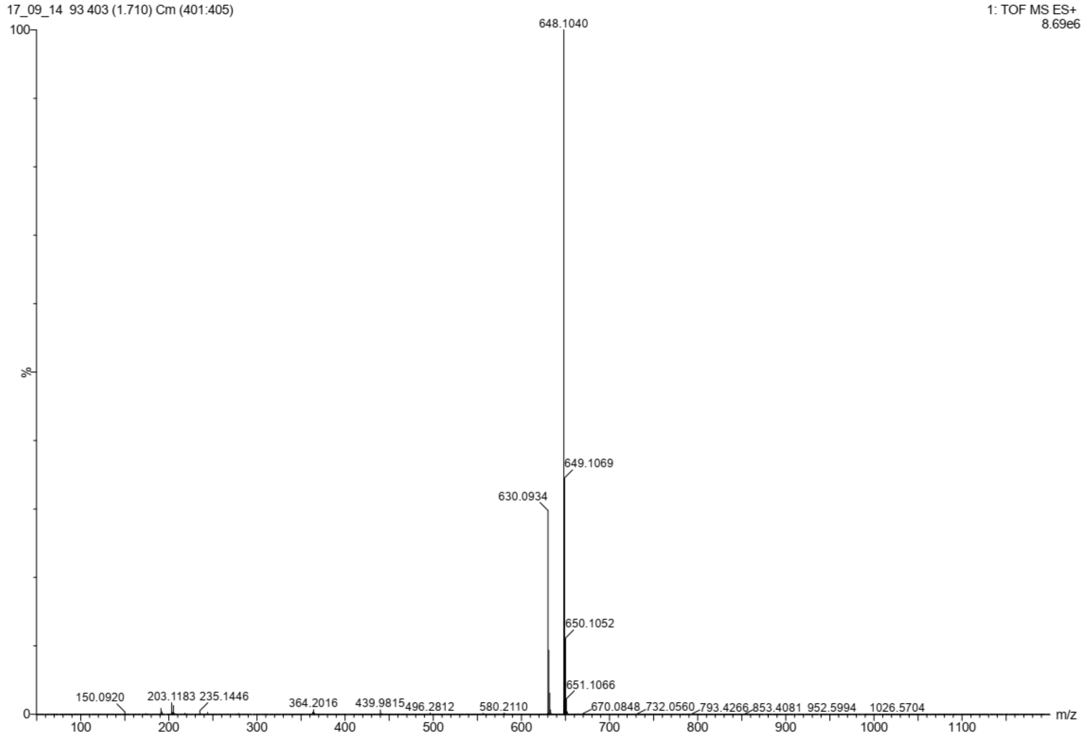

HRMS for **4k**

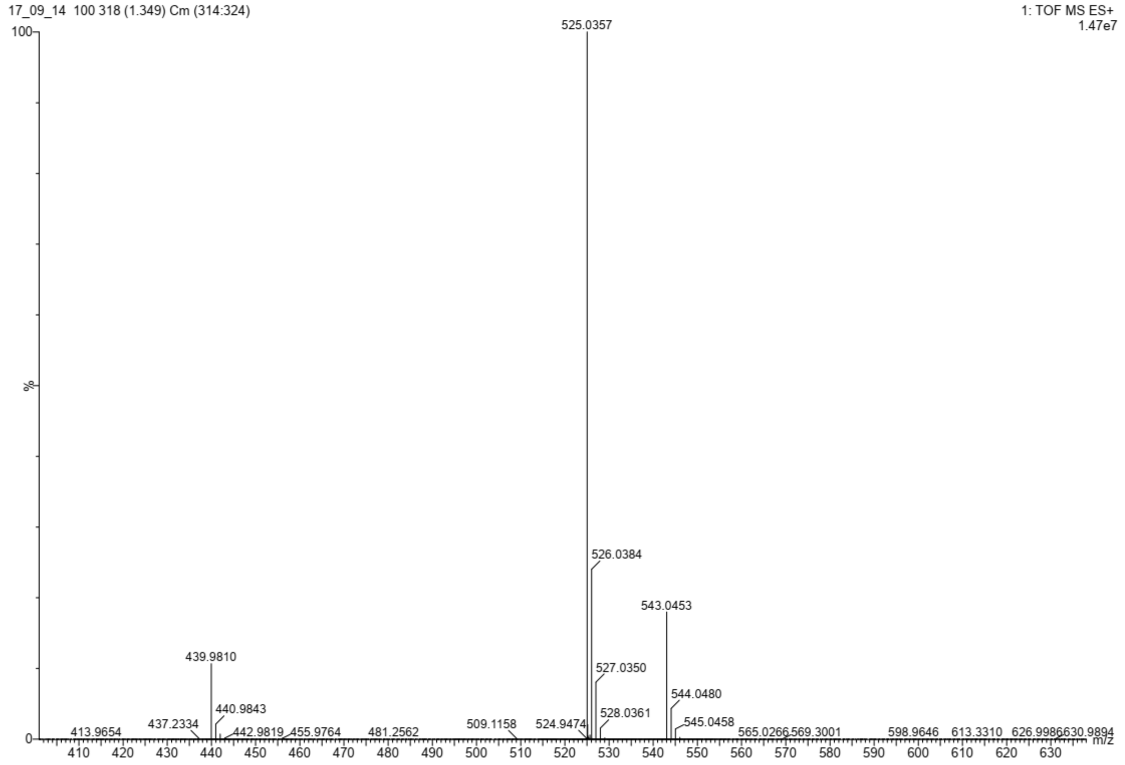


HRMS for **4l**

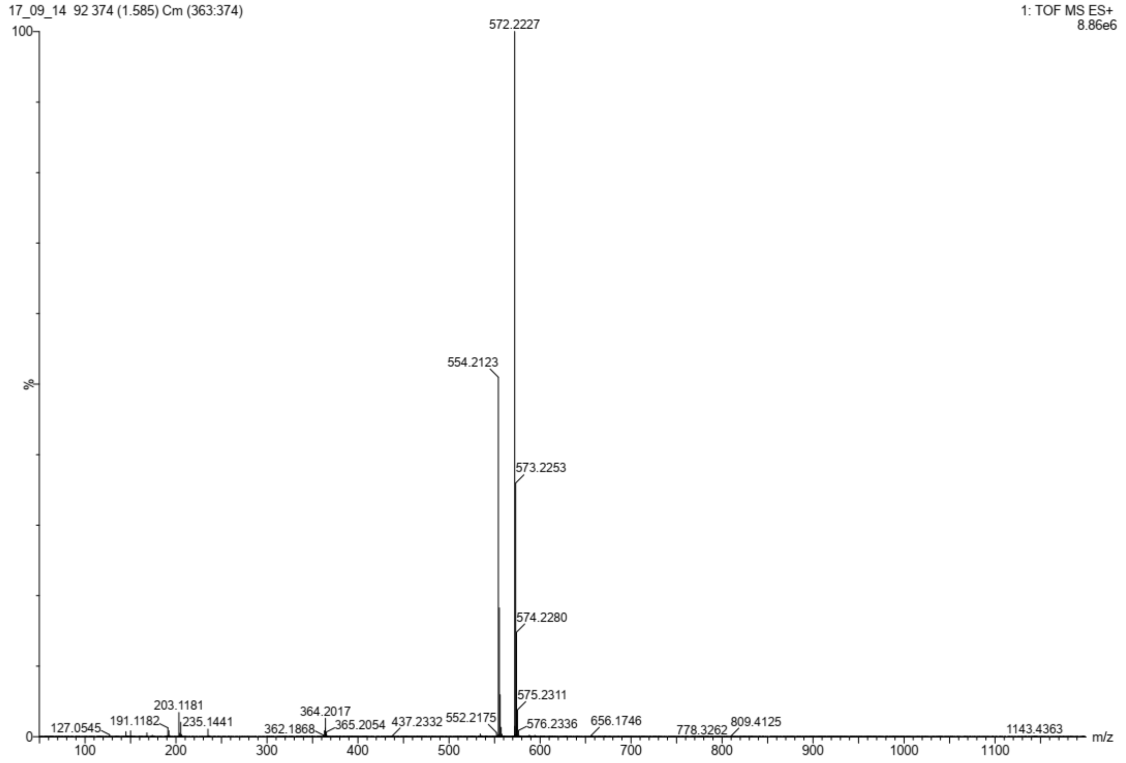

HRMS for **4m**

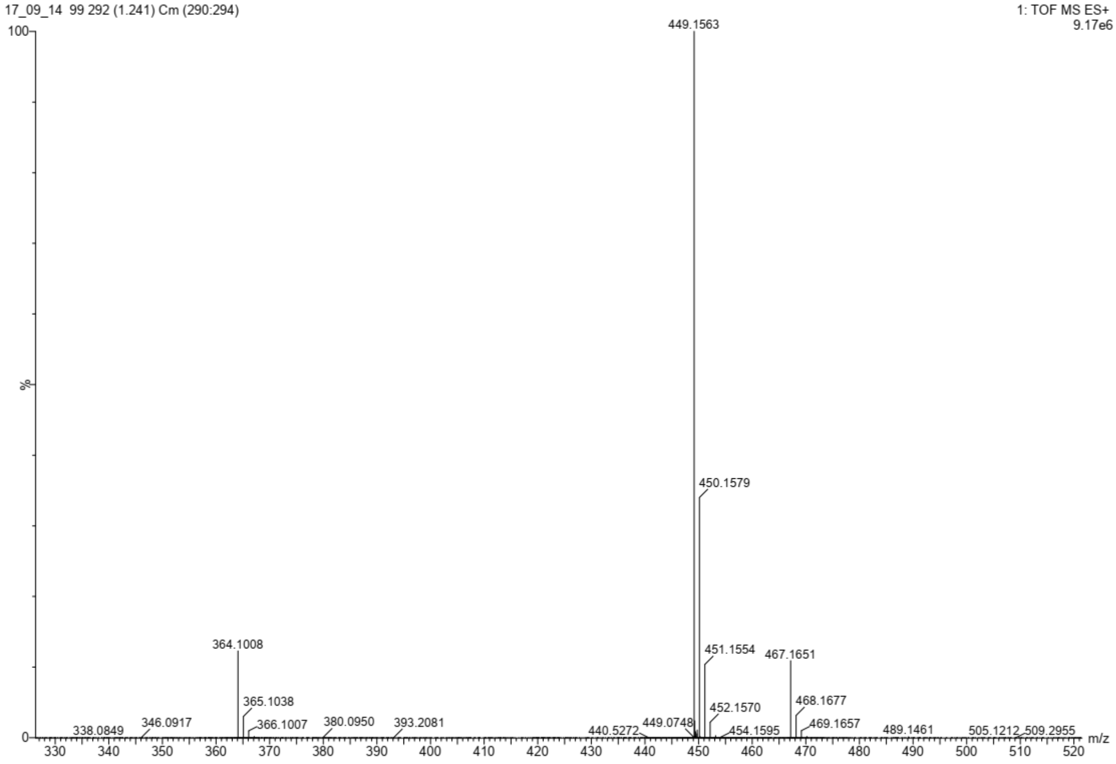


HRMS for **4n**

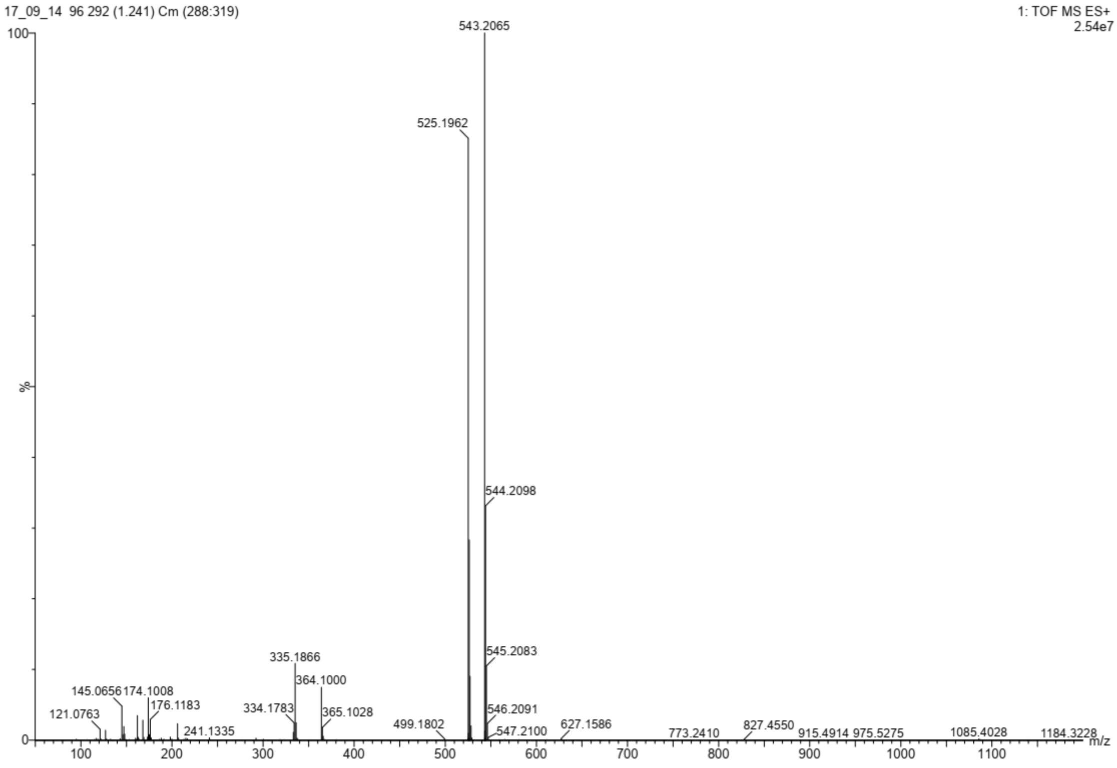

HRMS for **4o**

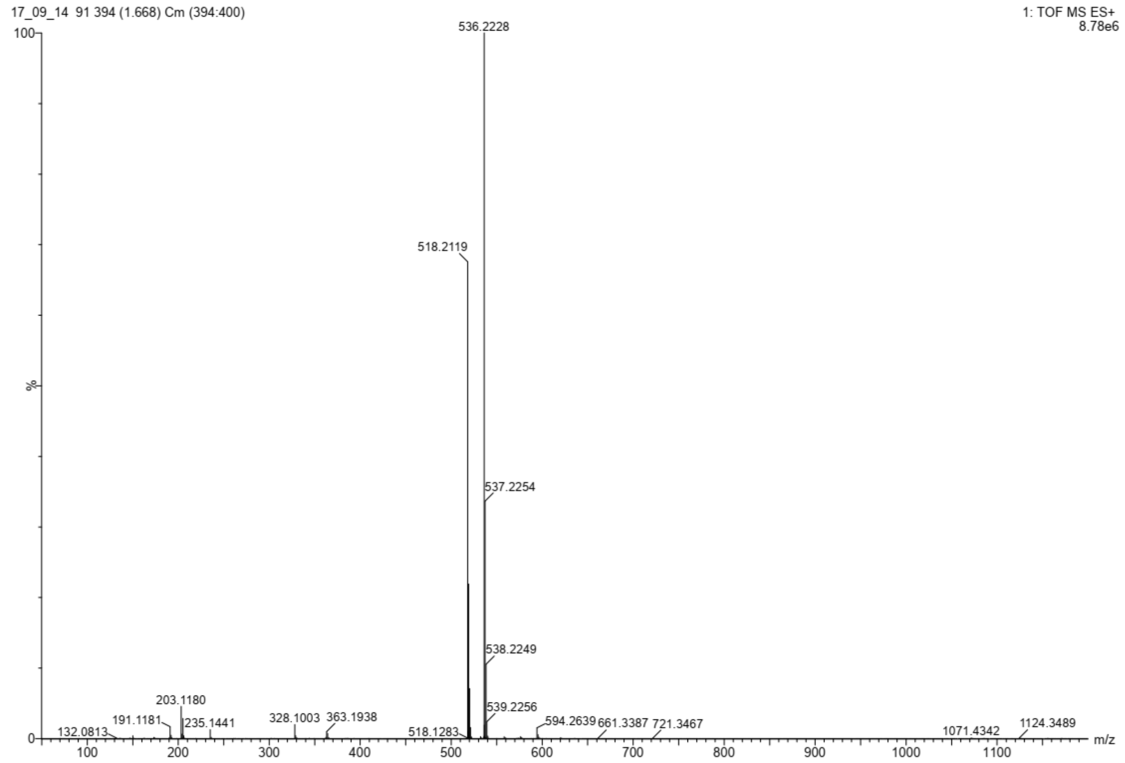


HRMS for **4p**

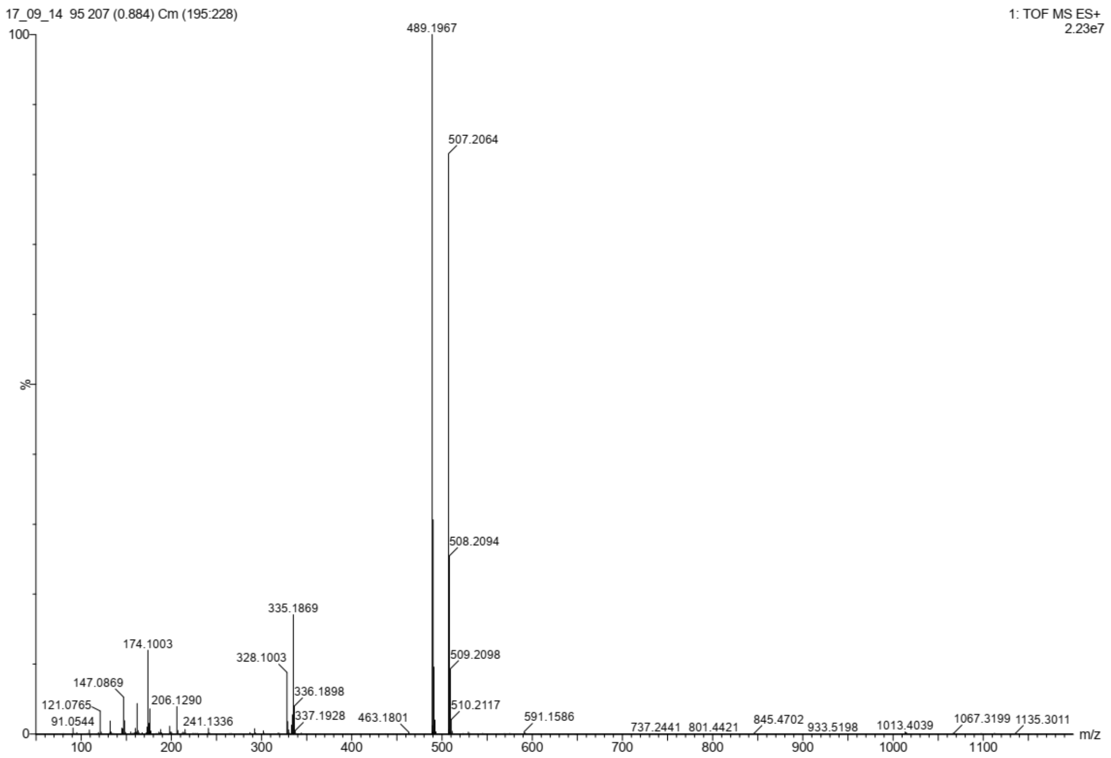

HRMS for **4q**

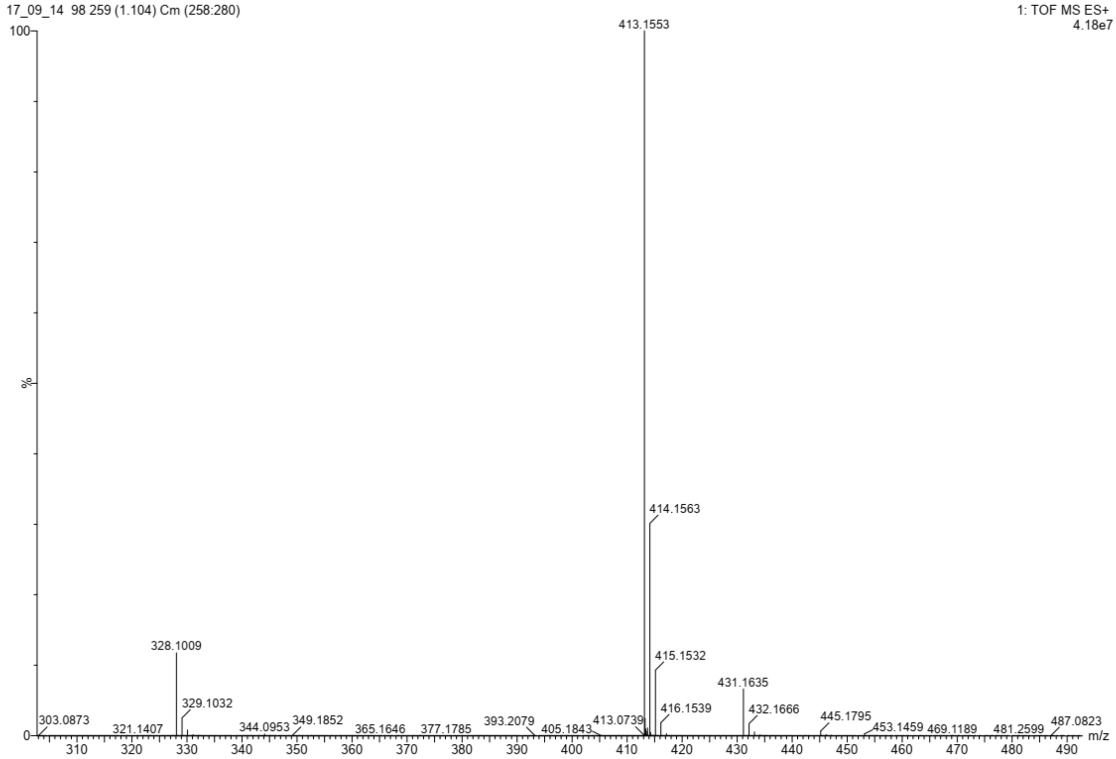


HRMS for **4r**

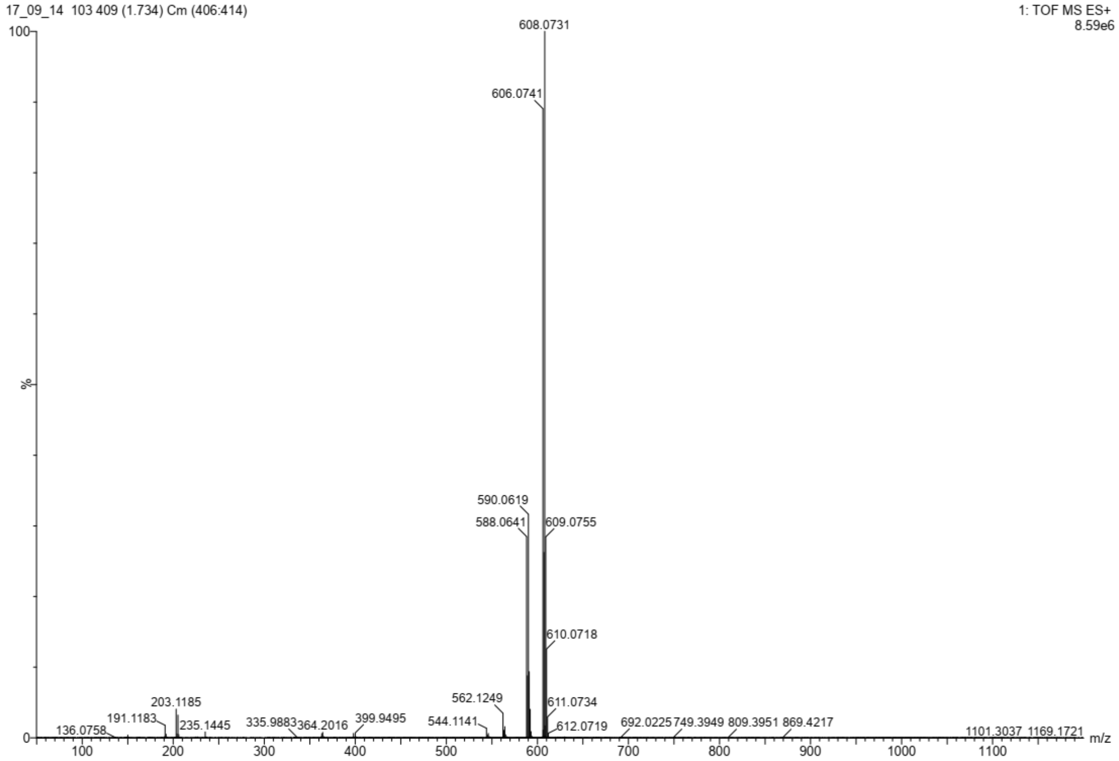

HRMS for **4s**

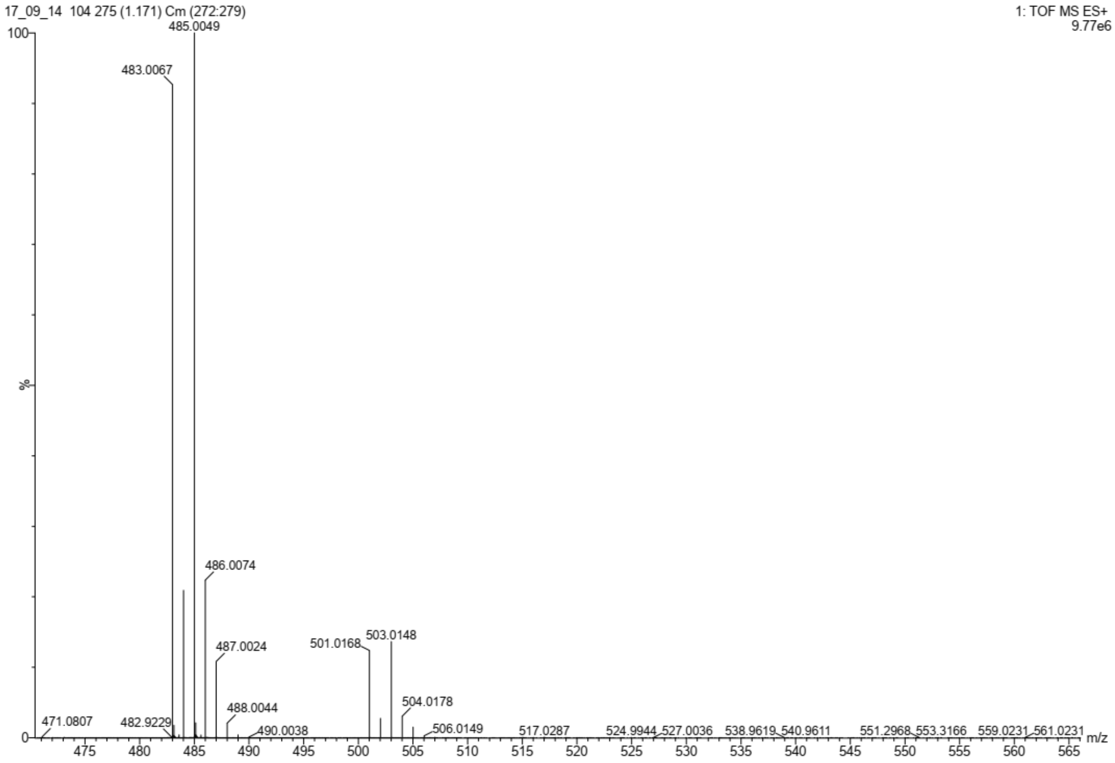


HRMS for **4t**

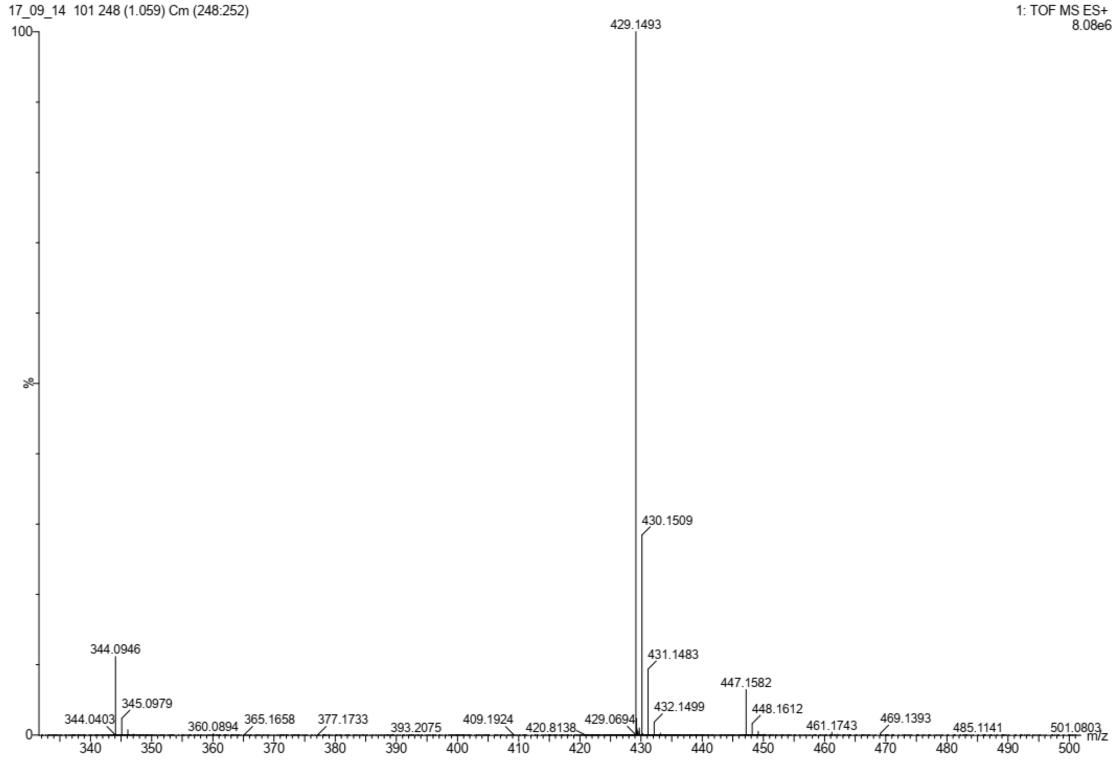

HRMS for **4u**

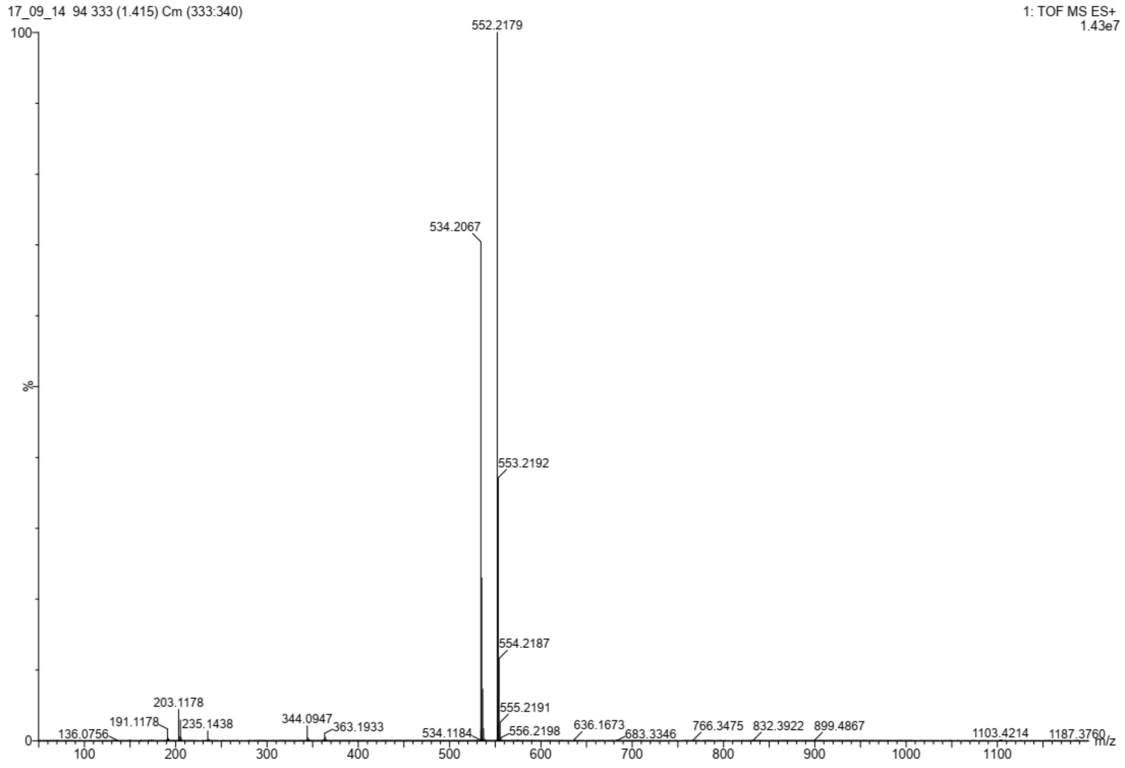


HRMS for **4v**

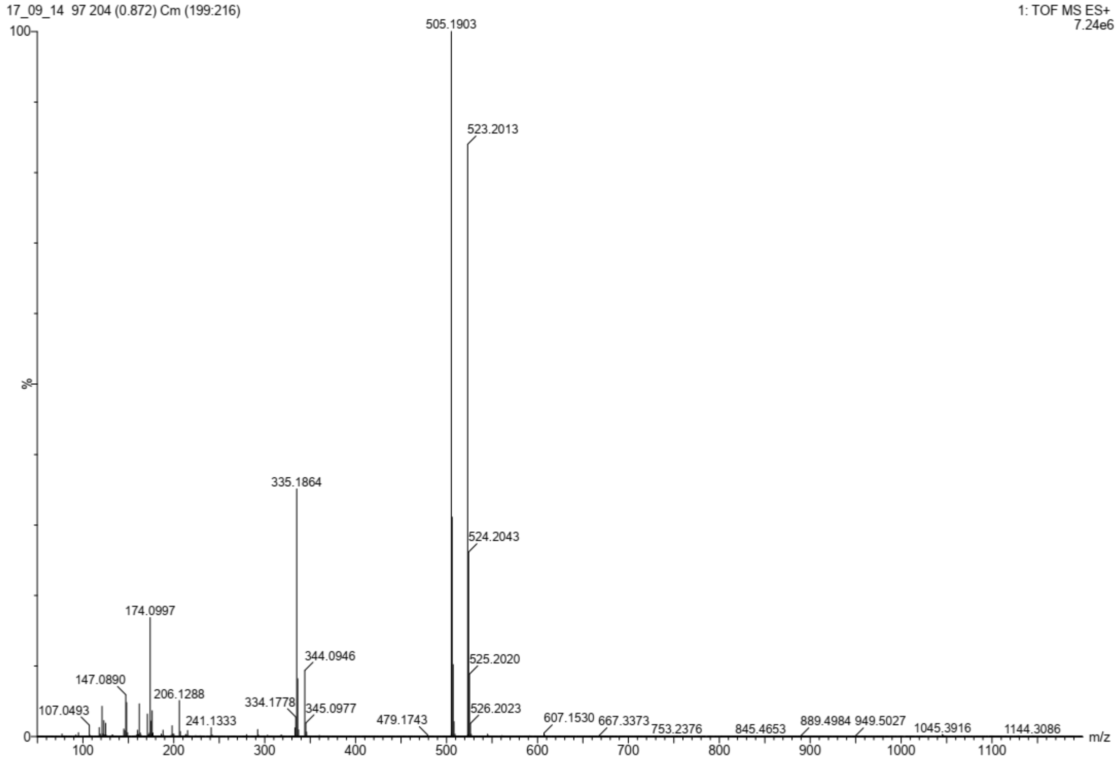

**4. IC$_{50}$ graphs for compounds 4a-4v and clozapine in 5-HT$_6$ radioligand binding assay.**

IC$_{50}$ graph for **4a**

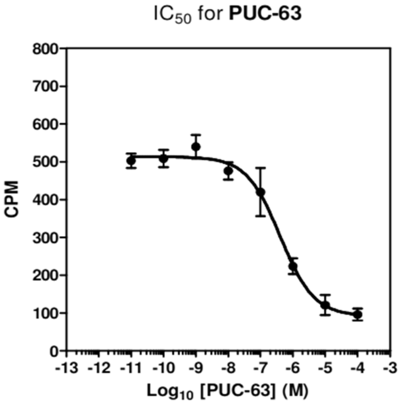


IC$_{50}$ graph for **4b**

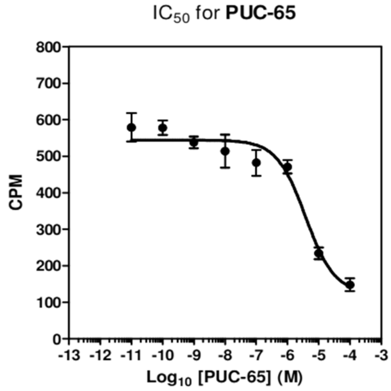


IC$_{50}$ graph for **4c**

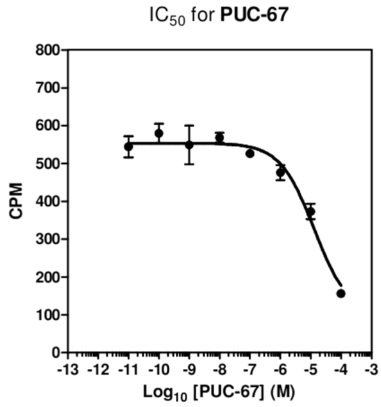


IC$_{50}$ graph for **4d**

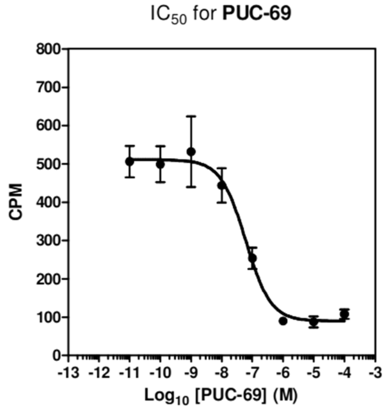


IC$_{50}$ graph for **4e**

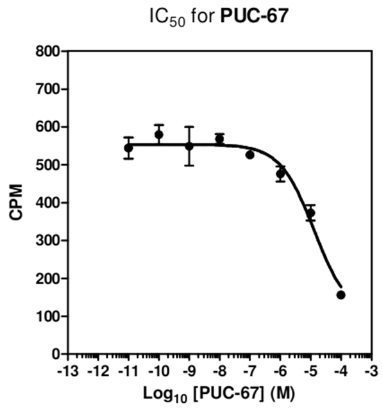


IC$_{50}$ graph for **4f**

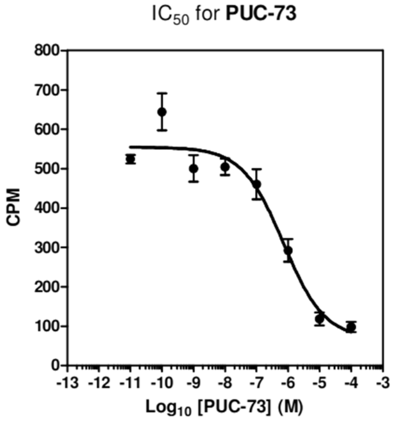

IC$_{50}$ graph for **4g**

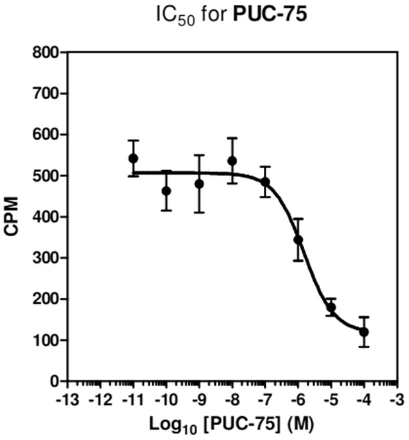


IC$_{50}$ graph for **4h**

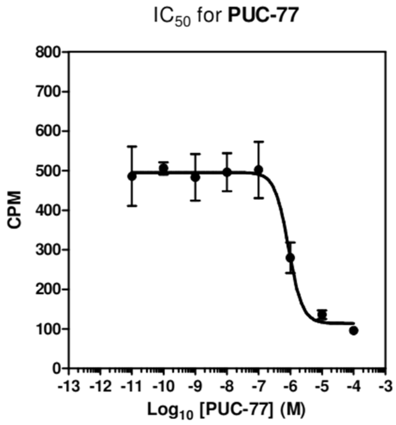


IC$_{50}$ graph for **4i**

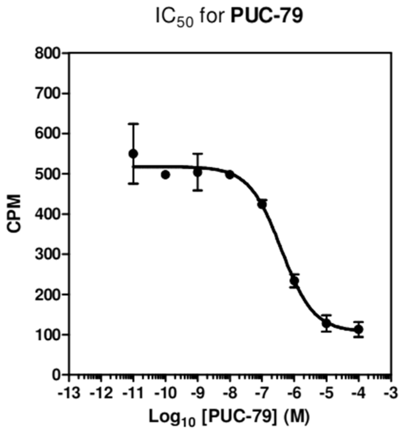


IC$_{50}$ graph for **4j**

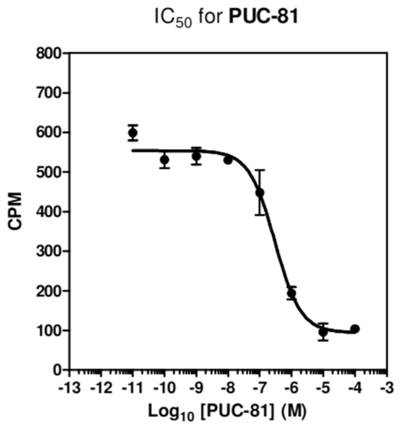


IC$_{50}$ graph for **4k**

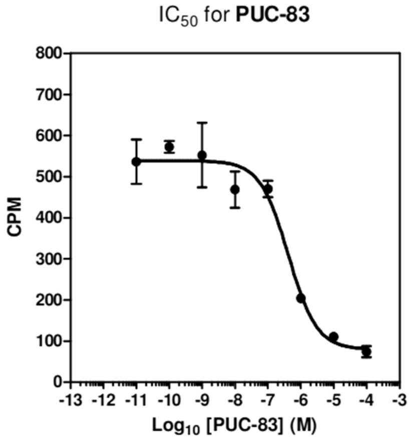


IC$_{50}$ graph for **4l**

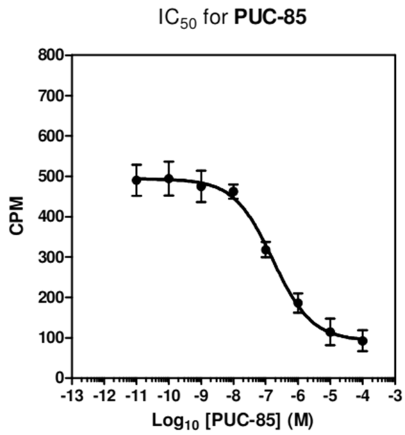

IC50 graph for **4m**

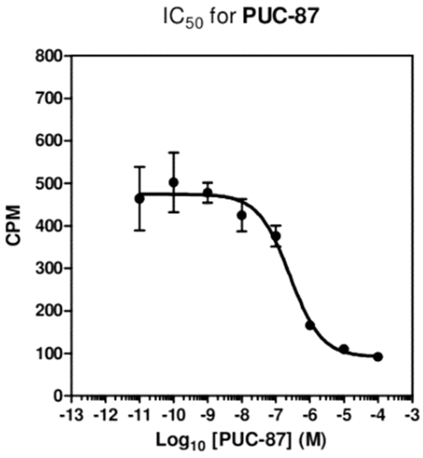


IC50 graph for **4n**

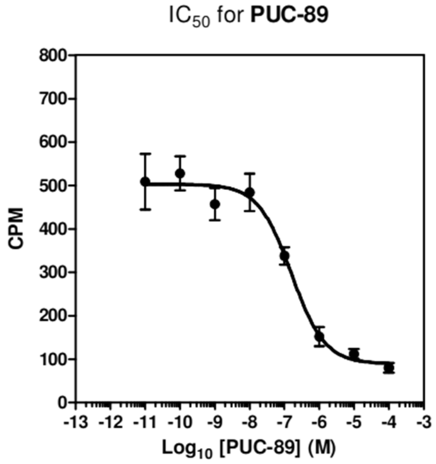


IC50 graph for **4o**

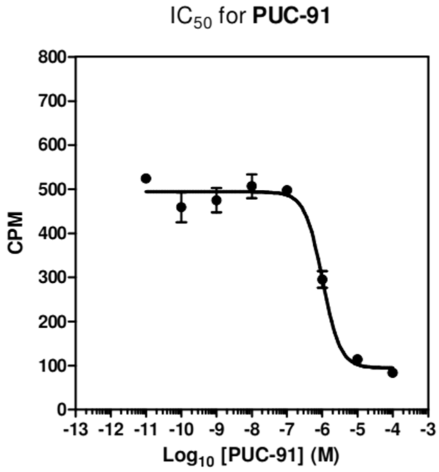


IC50 graph for **4p**

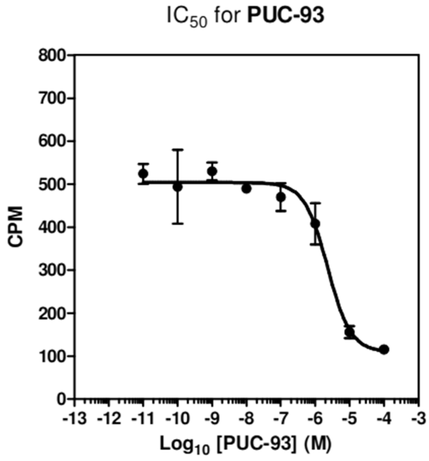


IC50 graph for **4q**

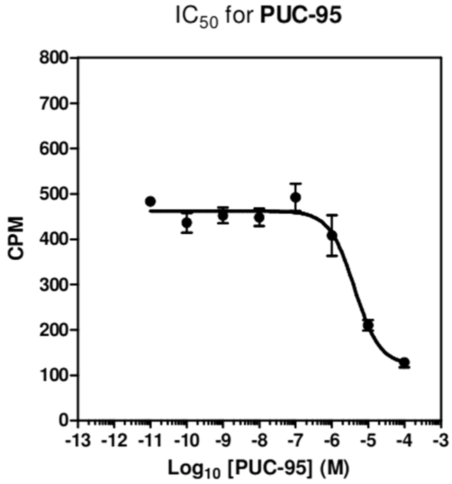


IC50 graph for **4r**

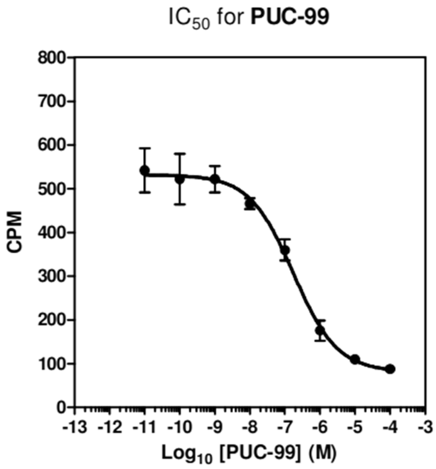

IC$_{50}$ graph for **4s**

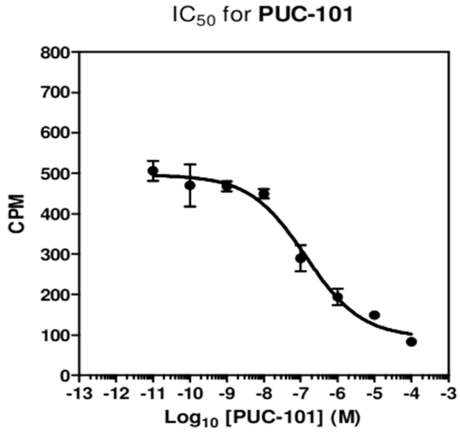


IC$_{50}$ graph for **4t**

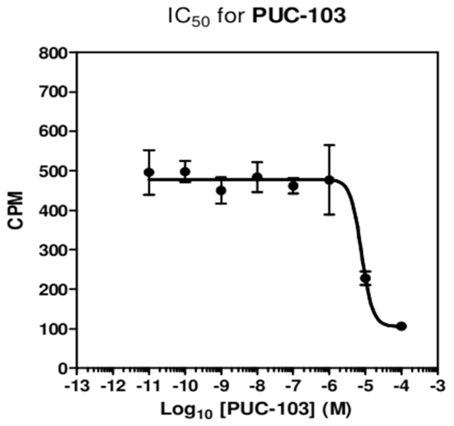


IC$_{50}$ graph for **4u**

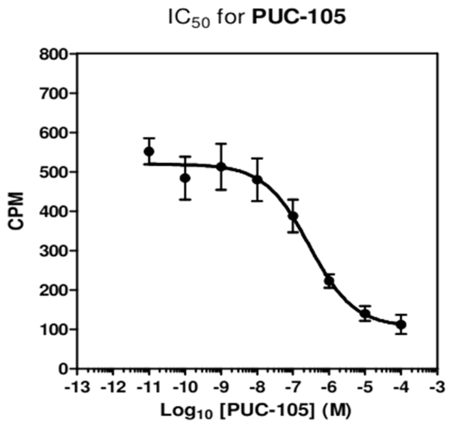


IC$_{50}$ graph for **4v**

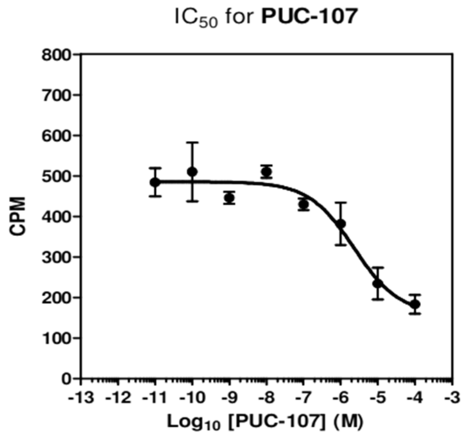


IC$_{50}$ graph for Clozapine

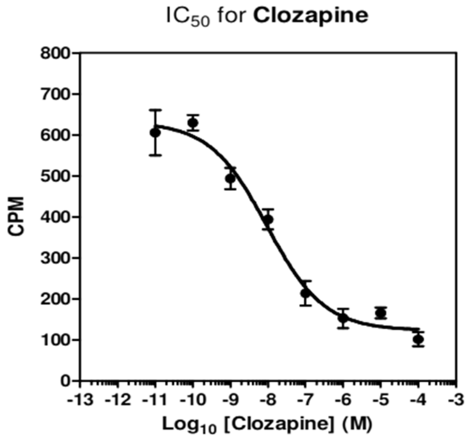

## 5. 3D-QSAR

**Table S1.** Statistical parameters and Field combinations for CoMFA and CoMSIA.

| Model | $q^2$ | N | SEP | SEE | $r^2$ | F | Field Contributions | | | | |
|---|---|---|---|---|---|---|---|---|---|---|---|
| | | | | | | | S | E | H | D | A |
| CoMFA-S | 0.665 | 3 | 0.410 | 0.224 | 0.900 | 81.432 | 1 | | | | |
| CoMFA-E | 0.64 | 2 | 0.418 | 0.222 | 0.898 | 123.484 | | 1 | | | |
| CoMFA-SE | 0.653 | 2 | 0.41 | 0.243 | 0.879 | 101.243 | 0.449 | 0.551 | | | |
| CoMSIA-S | 0.679 | 3 | 0.402 | 0.283 | 0.841 | 47.618 | 1 | | | | |
| CoMSIA-E | 0.643 | 4 | 0.431 | 0.179 | 0.939 | 99.431 | | 1 | | | |
| CoMSIA-H | 0.523 | 1 | 0.472 | 0.371 | 0.706 | 69.64 | | | 1 | | |
| CoMSIA-D | 0.384 | 7 | 0.603 | 0.213 | 0.923 | 39.279 | | | | 1 | |
| CoMSIA-A | 0.512 | 5 | 0.515 | 0.249 | 0.885 | 38.650 | | | | | 1 |
| CoMSIA-SE | 0.692 | 5 | 0.409 | 0.128 | 0.97 | 161.624 | 0.392 | 0.608 | | | |
| CoMSIA-SEH | 0.617 | 3 | 0.439 | 0.233 | 0.892 | 74.036 | 0.248 | 0.375 | 0.377 | | |
| CoMSIA-SEHD | 0.578 | 4 | 0.469 | 0.202 | 0.922 | 76.661 | 0.2 | 0.303 | 0.296 | 0.2 | |
| CoMSIA-SEHA | 0.596 | 3 | 0.45 | 0.254 | 0.871 | 61.03 | 0.191 | 0.293 | 0.304 | | 0.212 |
| CoMSIA-SED | 0.657 | 7 | 0.45 | 0.099 | 0.983 | 194.743 | 0.28 | 0.449 | | 0.271 | |
| CoMSIA-SEA | 0.65 | 5 | 0.442 | 0.147 | 0.96 | 120.809 | 0.28 | 0.426 | | | 0.293 |
| CoMSIA-SEDA | 0.613 | 5 | 0.458 | 0.167 | 0.949 | 92.828 | 0.219 | 0.338 | | 0.217 | 0.226 |
| CoMSIA-SH | 0.577 | 2 | 0.453 | 0.312 | 0.799 | 55.626 | 0.376 | | 0.624 | | |
| CoMSIA-SD | 0.558 | 5 | 0.49 | 0.193 | 0.931 | 67.834 | 0.542 | | | 0.458 | |
| CoMSIA-SA | 0.597 | 4 | 0.459 | 0.245 | 0.885 | 50.031 | 0.465 | | | | 0.535 |
| CoMSIA-SHD | 0.541 | 2 | 0.472 | 0.334 | 0.769 | 46.684 | 0.251 | | 0.402 | 0.347 | |
| CoMSIA-SHA | 0.562 | 3 | 0.469 | 0.273 | 0.852 | 51.754 | 0.271 | | 0.426 | | 0.303 |
| CoMSIA-SDA | 0.53 | 5 | 0.505 | 0.206 | 0.922 | 58.858 | 0.337 | | | 0.303 | 0.36 |
| CoMSIA-SHDA | 0.524 | 3 | 0.489 | 0.292 | 0.83 | 43.979 | 0.204 | | 0.321 | 0.248 | 0.227 |
| CoMSIA-EH | 0.575 | 4 | 0.471 | 0.163 | 0.949 | 120.591 | | 0.52 | 0.48 | | |
| CoMSIA-ED | 0.616 | 7 | 0.476 | 0.121 | 0.975 | 129.339 | | 0.63 | | 0.37 | |
| CoMSIA-EA | 0.605 | 5 | 0.463 | 0.168 | 0.948 | 91.456 | | 0.587 | | | 0.413 |
| CoMSIA-EHD | 0.549 | 2 | 0.468 | 0.3 | 0.814 | 61.186 | | 0.342 | 0.362 | 0.296 | |
| CoMSIA-EHA | 0.561 | 5 | 0.488 | 0.141 | 0.963 | 131.673 | | 0.374 | 0.358 | | 0.268 |
| CoMSIA-EDA | 0.588 | 7 | 0.493 | 0.12 | 0.976 | 131.854 | | 0.44 | | 0.276 | 0.283 |
| CoMSIA-EHDA | 0.531 | 5 | 0.504 | 0.153 | 0.957 | 111.34 | | 0.301 | 0.291 | 0.198 | 0.21 |
| CoMSIA-HD | 0.476 | 2 | 0.504 | 0.336 | 0.767 | 46.047 | | | 0.549 | 0.451 | |
| CoMSIA-HA | 0.504 | 2 | 0.49 | 0.333 | 0.771 | 47.145 | | | 0.588 | | 0.412 |
| CoMSIA-HDA | 0.474 | 2 | 0.505 | 0.349 | 0.749 | 41.769 | | | 0.392 | 0.336 | 0.272 |
| CoMSIA-DA | 0.453 | 7 | 0.568 | 0.18 | 0.945 | 56.843 | | | | 0.457 | 0.543 |
| CoMSIA-ALL | 0.566 | 3 | 0.467 | 0.27 | 0.855 | 52.887 | 0.155 | 0.238 | 0.246 | 0.19 | 0.171 |

[a] $q^2$ = the square of the LOO cross-validation (CV) coefficient; N = the optimum number of components; SEP = standard error of prediction; SEE is the standard error of estimation of non CV analysis; $r^2_{ncv}$ is the square of the non CV coefficient; F is the F-test value; S, E, H, D and A are the steric, electrostatic, hydrophobic, hydrogen-bond donor, and hydrogen-bond acceptor contributions respectively.

## External validation

The models were subjected to external validation criteria according to the proposed test by Golbraikh and Tropsha [1,2], which considers a QSAR model predictive, if some of the following conditions are satisfied:

$$q^2 > 0.5 \quad (2)$$

$$r^2 > 0.6 \quad (3)$$

$$\frac{|r^2 - r_0^2|}{r^2} < 0.1 \text{ or } \frac{|r^2 - r'^2_0|}{r^2} < 0.1 \quad (4)$$

$$0.85 \leq k \leq 1.15 \text{ or } 0.85 \leq k' \leq 1.15 \quad (5)$$

$$|r_0^2 - r'^2_0| < 0.3 \quad (6)$$

$$r_m^2 = r^2 \left(1 - \left|\sqrt{r^2 - r_0^2}\right|\right) \quad (7)$$

Where $r^2$ and $r_0^2$ are the squared correlation coefficients between the observed and predicted activities of the test set with and without the (0,0) intercept, respectively [1,3].

**Table S2.** Summary of external validation parameters for CoMFA and CoMSIA.

| Condition | Parameters | Threshold value | CoMFA | CoMSIA |
|---|---|---|---|---|
| 1 | $q^2$ | >0.5 | 0.653 | 0.692 |
| 2 | $r_{test}^2$ | >0.6 | 0.786 | 0.726 |
| 3a | $r_0^2$ | Close to value of $r_{test}^2$ | 0.999 | 0.999 |
| 3b | $r'^2_0$ | Close to value of $r_{test}^2$ | 0.999 | 0.999 |
| 4a | $k$ | $0.85 < k < 1.15$ | 0.995 | 1.004 |
| 4b | $k'$ | $0.85 < k' < 1.15$ | 1.004 | 0.995 |
| 5a | $|(r^2 - r_0^2)|/r^2$ | <0.1 | 0.271 | 0.376 |
| 5b | $|(r^2 - r'^2_0)|/r^2$ | <0.1 | 0.271 | 0.376 |
| 6 | $|r_0^2 - r'^2_0|$ | <0.3 | 0.000 | 0.000 |
| 7 | $r_m^2$ | >0.5 | 0.423 | 0.347 |

$q^2$ and $r^2_{test}$ are the same parameters as listed in table 1; $r_0^2$ and k are the correlation coefficient between the actual and predicted activities for test set and the respective slope of regression; and $r_0'^2$ and k′ are the correlation coefficient between the predicted and actual activities for test set and the respective slope of regression. $r^2_m$ is the $r^2$ metric.

**Table S3.** Experimental and predicted $pIC_{50}$ and residual values for analyzed compounds according to CoMFA and CoMSIA.

| Molecule | Exp. pKi(M) | CoMFA | | CoMSIA | |
|---|---|---|---|---|---|
| | | Predicted pKi (M) | Residual | Predicted pKi (M) | Residual |
| **4aR** | 6.409 | 6.459 | -0.05 | 6.500 | -0.09 |
| **4aS** | 6.409 | 6.534 | -0.13 | 6.548 | -0.14 |
| **4bR** | 5.440 | 5.762 | -0.32 | 5.546 | -0.11 |
| **4bS** | 5.440 | 5.668 | -0.23 | 5.442 | 0.00 |
| **4cR** | 4.902 | 4.960 | -0.06 | 5.046 | -0.14 |
| **4cS** | 4.902 | 5.089 | -0.19 | 4.928 | -0.03 |
| **4dR** | 7.234 | 7.115 | 0.12 | 7.381 | -0.15 |
| **4dS** | 7.234 | 6.699 | 0.54 | 6.921 | 0.31 |
| **4eR*** | 6.096 | 6.083 | 0.01 | 6.215 | -0.12 |
| **4eS** | 6.096 | 6.093 | 0.00 | 6.022 | 0.07 |
| **4fR** | 6.165 | 5.965 | 0.20 | 6.211 | -0.05 |
| **4fS** | 6.165 | 6.179 | -0.01 | 6.034 | 0.13 |
| **4gR*** | 5.840 | 5.912 | -0.07 | 6.008 | -0.17 |
| **4gS** | 5.840 | 6.157 | -0.32 | 5.718 | 0.12 |
| **4hR*** | 6.071 | 5.950 | 0.12 | 6.134 | -0.06 |
| **4hS*** | 6.071 | 5.861 | 0.21 | 6.192 | -0.12 |
| **4iR** | 6.419 | 6.674 | -0.25 | 6.517 | -0.10 |
| **4iS** | 6.419 | 6.567 | -0.15 | 6.561 | -0.14 |
| **4jR** | 6.539 | 6.471 | 0.07 | 6.694 | -0.15 |
| **4jS** | 6.539 | 6.602 | -0.06 | 6.551 | -0.01 |
| **4kR*** | 6.395 | 5.767 | 0.63 | 5.567 | 0.83 |
| **4kS** | 6.395 | 6.241 | 0.15 | 6.468 | -0.07 |
| **4lR*** | 6.796 | 6.779 | 0.02 | 6.965 | -0.17 |
| **4lS*** | 6.796 | 6.829 | -0.03 | 6.486 | 0.31 |
| **4mR** | 6.607 | 6.510 | 0.10 | 6.533 | 0.07 |
| **4mS*** | 6.607 | 6.270 | 0.34 | 6.575 | 0.03 |
| **4nR** | 6.815 | 6.273 | 0.54 | 6.565 | 0.25 |
| **4nS** | 6.815 | 6.973 | -0.16 | 6.793 | 0.02 |
| **4oR*** | 6.012 | 6.083 | -0.07 | 6.327 | -0.32 |
| **4oS** | 6.012 | 6.275 | -0.26 | 6.044 | -0.03 |
| **4pR*** | 5.659 | 5.920 | -0.26 | 5.780 | -0.12 |
| **4pS*** | 5.659 | 5.998 | -0.34 | 5.693 | -0.03 |
| **4qR** | 5.395 | 5.040 | 0.36 | 5.310 | 0.09 |
| **4qS** | 5.395 | 5.534 | -0.14 | 5.415 | -0.02 |
| **4rR** | 6.775 | 6.655 | 0.12 | 6.835 | -0.06 |
| **4rS** | 6.775 | 6.922 | -0.15 | 6.659 | 0.12 |
| **4sR** | 6.886 | 5.322 | 1.56 | 5.449 | 1.44 |
| **4sS** | 6.886 | 6.579 | 0.31 | 6.805 | 0.08 |
| **4tR** | 5.125 | 4.840 | 0.29 | 5.034 | 0.09 |
| **4tS** | 5.125 | 5.155 | -0.03 | 5.088 | 0.04 |
| **4uR*** | 6.567 | 6.333 | 0.23 | 6.637 | -0.07 |

| | | | | | |
|---|---|---|---|---|---|
| **4uS*** | 6.567 | 6.456 | 0.11 | 6.083 | 0.48 |
| **4vR** | 5.660 | 5.863 | -0.20 | 5.602 | 0.06 |
| **4vS** | 5.660 | 5.896 | -0.24 | 5.730 | -0.07 |

* test set compounds.

## Applicability domain calculation

The AD was evaluated based on the simple standardization method reported by Roy et al. [4]. First, each descriptor "i" for each compound "k" is standardized ($S_{ik}$). Every compound must have a maximum value $[S_i]_{max(k)} \leq 3$. In the case that $[S_i]_{max(k)} > 3$ and its minimum value $[s_i]_{min(k)} < 3$, then the $S_{new(k)}$ parameter must be calculated and has to fulfill the condition: $S_{new(k)} = \bar{S}_k + 1.28 * \sigma_{S_k}$, where $\bar{S}_k$ is the mean of $Sik$ values for compound $k$ and $\sigma_{S_k}$ is the standard deviation for such values. The software is available free of charge on the authors' website: http://dtclab.webs.com/software-tools and http://teqip.jdvu.ac.in/QSAR_Tools/.

## 6. References


1. Golbraikh, A.; Tropsha, A. Beware of q2! *Journal of Molecular Graphics and Modelling* **2002**, *20*, 269-276, doi:https://doi.org/10.1016/S1093-3263(01)00123-1.
2. Tropsha, A. Best Practices for QSAR Model Development, Validation, and Exploitation. *Molecular Informatics* **2010**, *29*, 476-488, doi:https://doi.org/10.1002/minf.201000061.
3. Roy, K.; Chakraborty, P.; Mitra, I.; Ojha, P.K.; Kar, S.; Das, R.N. Some case studies on application of "rm2" metrics for judging quality of quantitative structure–activity relationship predictions: Emphasis on scaling of response data. *Journal of Computational Chemistry* **2013**, *34*, 1071-1082, doi:https://doi.org/10.1002/jcc.23231.
4. Roy, K.; Kar, S.; Ambure, P. On a simple approach for determining applicability domain of QSAR models. *Chemometrics and Intelligent Laboratory Systems* **2015**, *145*, 22-29, doi:https://doi.org/10.1016/j.chemolab.2015.04.013.
